# Brain PDGFR*β* ^+^ Cells Exhibit Diverse Reactive Phenotypes After Stroke Without Requiring KLF4

**DOI:** 10.1002/glia.70232

**Published:** 2026-10-31

**Authors:** Daniel Manrique‐Castano, Ayman ElAli

**Affiliations:** ^1^ Neuroscience Axis, Research Center of CHU de Québec‐Université Laval Quebec City Quebec Canada; ^2^ Department of Psychiatry and Neuroscience, Faculty of Medicine Université Laval Quebec City Quebec Canada

**Keywords:** ischemic stroke, KLF4, PDGFR*β* ^+^ cells, point pattern analysis (PPA), tissue scar, topological data analysis (TDA)

## Abstract

The pathobiology of ischemic stroke is associated with the activation of brain platelet‐derived growth factor receptor (PDGFR)*β* ^+^ perivascular cells. While Krüppel‐like factor (KLF)4 is a well‐established mediator of peripheral PDGFR*β* ^+^ cell activation, its role in brain PDGFR*β* ^+^ cells remains elusive. Herein, we aimed to characterize the spatiotemporal reactivity of brain PDGFR*β* ^+^ cells, while assessing KLF4 contribution upon experimental ischemic stroke using transgenic mice that enable tracking PDGFR*β* ^+^ cells or conditionally depleting KLF4 in PDGFR*β* ^+^ cells. Moving beyond the qualitative descriptors, we sought to develop point pattern analysis (PPA) and topological data analysis (TDA) workflows to generate robust and reusable mathematical models to assess spatial cell responses and tissue scar topological changes. First, we report the emergence of phenotypically distinct reactive parenchymal PDGFR*β* ^+^ cells, which cannot be causally and exclusively linked, as it is currently proposed, to local cell proliferation or adjacent vascular detachment. Furthermore, we show that KLF4 is barely expressed in brain PDGFR*β* ^+^ cells under normal conditions and that it is only marginally induced in reactive PDGFR*β* ^+^ cells at the injured brain. Notably, the conditional attenuation of KLF4 expression specifically in reactive PDGFR*β* ^+^ cells does not alter spatial cellular distribution nor scar organization. This suggests that, in contrast to peripheral PDGFR*β* ^+^ cells upon ischemic insults, KLF4 is not a main regulator of the reactivity of brain cells in response to ischemic stroke. Ultimately, our findings supported by open‐source spatiotemporal models indicate that the reactivity of brain PDGFR*β* ^+^ perivascular cells is highly heterogeneous and fundamentally distinct from peripheral PDGFR*β* ^+^ cells.

## Graphical abstract

- Brain PDGFR*β* ^+^ cells become reactive and adopt diverse phenotypes after stroke.
- Reactive PDGFR*β* ^+^ cells exhibit predictable spatial distribution relative to glial scar.
- KLF4 is not required to mediate the reactivity of brain PDGFR*β* ^+^ cells after stroke.

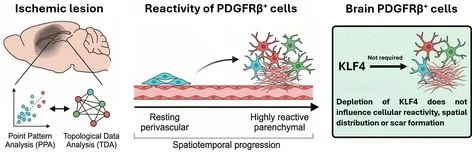

## 1 Introduction

Ischemic stroke triggers a highly orchestrated multicellular response, culminating in the formation of a neatly demarcated glial scar surrounding a fibrotic core (Adams and Gallo 2018; Fernandez‐Klett et al. 2013; R. Zhang et al. 2018). At the neurovascular interface, platelet‐derived growth factor receptor (PDGFR)*β* ^+^ perivascular cells are among the first responders to various pathological cues (Fernandez‐Klett et al. 2013; Rustenhoven et al. 2017; Duan et al. 2018). The current knowledge suggests that upon ischemic stroke PDGFR*β* ^+^ perivascular cells become reactive and rapidly detach from the adjacent vasculature, undergo massive local proliferation, and migrate towards the infarct core to orchestrate the fibrotic reaction, while providing spatial cues required for the organization of the glial scar (Dias et al. 2021; Shibahara et al. 2023). The precise spatiotemporal mechanisms governing brain PDGFR*β* ^+^ perivascular cell activation, phenotypic transition, and large‐scale reorganization over the course of brain injury progression after ischemic stroke remain incompletely understood.

In peripheral organs, zinc‐finger transcription factor Krüppel‐like factor (KLF)4 serves as an essential driver of PDGFR*β* ^+^ cell reactivity in response to hypoxic, ischemic, and fibrotic insults. This entails regulation of PDGFR*β* ^+^ cell reprogramming and de‐differentiation, as well as the release of extracellular matrix (ECM) proteins (Chandran et al. 2021; Ghaleb and Yang 2017; Shankman et al. 2015). KLF4‐mediated regulatory processes are assumed to be conserved across all PDGFR*β* ^+^ cell populations. However, recent evidence indicates that unlike PDGFR*β* ^+^ cells in peripheral organs, which originate from the mesoderm, brain PDGFR*β* ^+^ perivascular cells originate from the neuroectoderm (Korn et al. 2002). Yet, it remains unknown whether KLF4 regulatory functions are similarly involved in modulating the reactivity of brain PDGFR*β* ^+^ perivascular cells after ischemic stroke.

Herein, we elucidate the spatiotemporal dynamics of reactive brain PDGFR*β* ^+^ cells and define the specific contribution of KLF4 in experimental ischemic stroke. Using genetic tools in mice to track PDGFR*β* ^+^ cells (PDGFR*β* ^tdTomato^ mice) or conditionally downregulate KLF4 expression in PDGFR*β* ^+^ cells (PDGFR*β* ^KLF4‐KO^ mice), we moved beyond the conventional qualitative descriptive assessments of spatial cell responses within the injured brain. This was achieved by implementing novel automated workflows incorporating point pattern analysis (PPA) and topological data analysis (TDA) to establish robust, model‐based quantitative parameters of cell reorganization over the course of brain injury progression. Our spatial models reveal the emergence of reactive parenchymal PDGFR*β* ^+^ cells that are not causally linked to local cell proliferation. Notably, we demonstrate that the conditional downregulation of KLF4 in PDGFR*β* ^+^ cells does not affect the reactivity of cells nor the spatiotemporal architecture of glial and fibrotic scars. Collectively, our study, while providing new open‐source tools to assess cell spatial reorganization, demonstrates that reactive brain PDGFR*β* ^+^ cells are highly heterogeneous, implicating distinct regulatory mechanisms in comparison with peripheral perivascular cells upon injuries.

## 2 Materials and Methods

All raw data and analysis pipelines are publicly available. This article comprises a Zenodo repository hosting raw widefield and confocal microscopy images (Manrique‐Castano and ElAli 2024c) and an Open Science Framework (OSF) repository (Manrique‐Castano and ElAli 2024a) hosting a wide variety of resources, including low‐resolution images (.png), raw and processed datasets (.csv), point patterns (.RDS), point clouds (.npy), statistical models (.RDS), and statistical model summary tables (.tex/html). The OSF repository also comprises the pipelines used for image pre‐processing and analysis derived from FIJI (.ijm) (Schindelin et al. 2012), CellProfiler (.cpproj) (Stirling et al. 2021), Ilastik (.ilp) (Berg et al. 2019), and QuPath (.groovy) (Bankhead et al. 2017). To ensure full reproducibility and reuse, we host annotated Quarto (QN) (Allaire et al. 2022) and Jupyter (JN) notebooks encompassing data processing and statistical modeling in a versioned GitHub repository (Manrique‐Castano and ElAli 2024b). Additionally, we provide supplementary methods detailing the implemented analysis pipelines as well as Supporting Information of analyzed data and different used models presented as tables (.qmd) available in a repository in GitHub. Note that the main article and the supplementary methods have direct links to specific Zenodo, OSF, or GitHub resources. While some resources are rendered in their respective platform, others need to be processed in the corresponding software. Our experimental design and analysis workflow are summarized in Figure 1.

**FIGURE 1:**
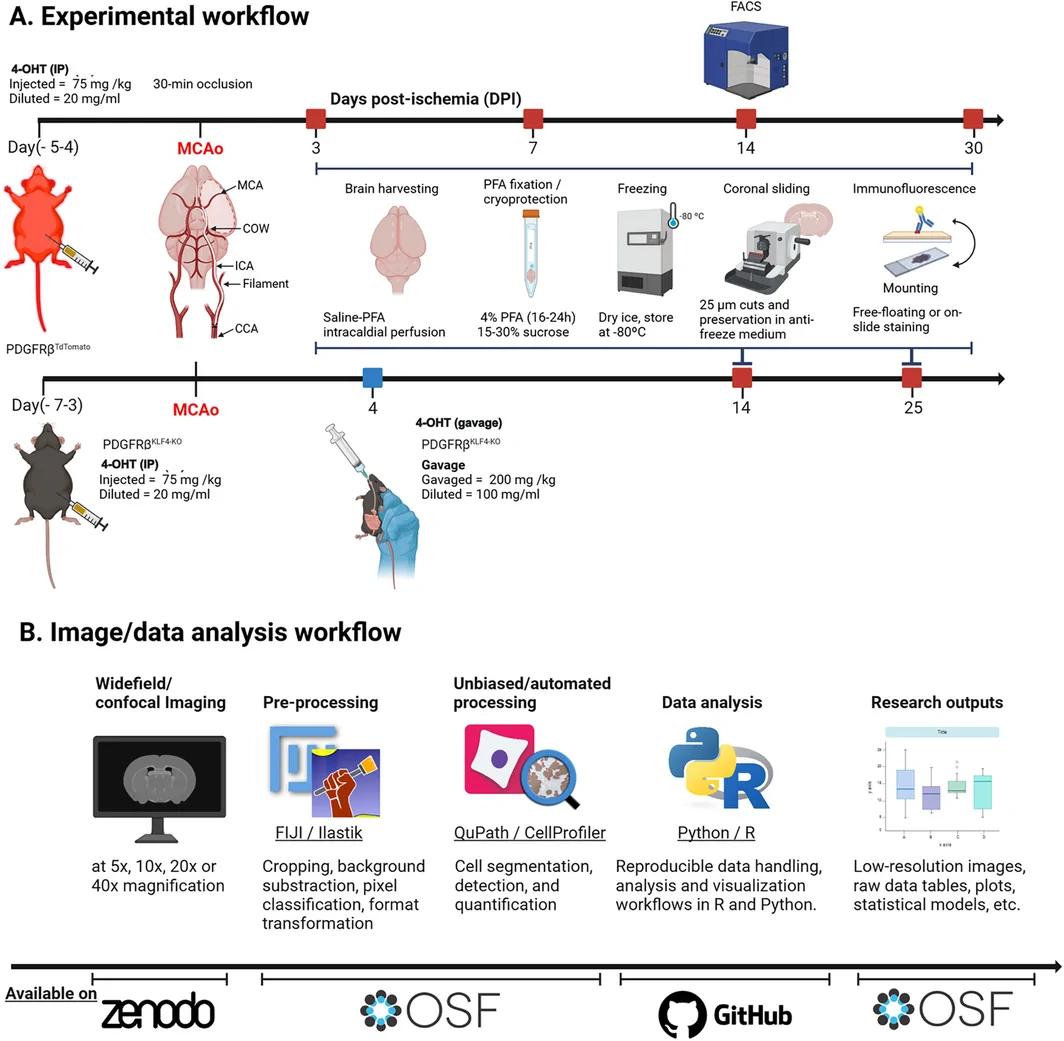
Experimental design and analysis workflows. (A) tdTomato was induced in PDGFR*β*⁺ cells via 4‐hydroxytamoxifen (4‐OHT) intraperitoneal (IP) administration in B6.Cg‐*Gt(ROSA)26Sor* ^tm14(CAGtdTomato)Hze^/J; B6.Cg‐Pdgfrb^tm1.1(cre/ERT2)Csln^/J mice [PDGFR*β* ^tdTomato^ reporter mice]. KLF4 was specifically depleted in PDGFR*β*⁺ cells either via tamoxifen IP (initiated at day 7 prior to ischemic stroke onset) or gavage (initiated at day 4 after ischemic stroke onset) administration in B6.129S6‐Klf4^tm1khk^/Mmmh; B6.Cg‐Pdgfrb^tm1.1(cre/ERT2)Csln^/J mice [PDGFR*β* ^KLF4‐KO^ mice]. Both genotypes, along with corresponding control or sham animals, underwent a 30‐min middle cerebral artery occlusion (MCAo) using a nylon‐coated monofilament (CCA: common carotid artery; ICA: internal carotid artery; COW: Circle of Willis; MCA: middle cerebral artery). Brains were harvested from PDGFR*β* ^tdTomato^ mice at 3, 7, 14, and 30 days post‐ischemic stroke (DPI) and processed for immunofluorescence (on‐slide or free‐floating) or for fluorescence‐activated cell sorting (FACS) at 14 DPI. For the PDGFR*β* ^KLF4‐KO^ animals, brains were harvested at 14 DPI for post‐ischemic stroke onset KLF4 depletion and at 25 DPI for pre‐ischemic stroke onset KLF4 depletion. Consistent protocols were applied for immunofluorescence across genotypes. (B) A schematic illustration of the image and data analysis workflow using open‐source software. Upon brain section imaging, preprocessing was performed using FIJI and/or Ilastik, followed by unbiased and automated cell detection with CellProfiler or QuPath. The results were analyzed using R or Python code. All raw images, data, and research outputs are available in our Zenodo (Manrique‐Castano and ElAli 2024c), OSF (ElAli and Manrique‐Castano 2024), or GitHub (Manrique‐Castaño and Interactions 2024) repositories, as depicted. The figure was created using BioRender https://BioRender.com/v11z645. High‐resolution figure: https://osf.io/jxb4z/files/y7csx.

### 2.1 Animals

Three to five months old C57BL6/J male mice were obtained from The Jackson Laboratory (Bar Harbor, ME, USA). Age‐matching transgenic mice heterozygous for B6.Cg‐PDGFR*β* ^tm1.1(cre/ERT2)Csln^/J (PDGFR*β* ^CreERT2^; #030201, The Jackson Laboratory) were crossbred with homozygous mice B6.Cg‐Gt(Rosa)26Sor^tm9(CAG‐tdTomato)Hze^ (Ai9; #007909, The Jackson Laboratory) to generate PDGFR*β* ^tdTomato^ reporter mice, or homozygous for B6.129S6‐Klf4^tm1khk^/Mmmh (KLF4^Flox^; RRID:MMRRC_029877‐MU, Mutant Mouse Resource & Research Centers (MMRRC), MO, USA) (PMID: 12015290; PMID: 15825076) to generate PDGFR*β* ^KLF4‐KO^ mice. In PDGFR*β* ^CreERT2^:Ai9 mice a *loxP*‐flanked STOP cassette, which prevents the transcription of a CAG promoter‐driven tdTomato, is inserted into Gt(ROSA)26Sor locus. Cre‐recombinase was induced via administration of 4‐hydroxytamoxifen (4‐OHT) (80 mg/kg in corn oil; Intraperitoneal; 1× daily for 2 days) prior to ischemic stroke to excise the flanked *loxP* sites, allowing tdTomato specific expression in PDGFR*β* ^+^ cells. In PDGFR*β* ^CreERT2^:KLF4^Flox^ mice, exons 2 and 3 of the *Klf4* gene was flanked with *loxP* sites in introns 1 and 3. In some experiments, Cre‐recombinase was induced via administration of 4‐OHT (200 mg/kg in corn oil; Oral gavage; 1× daily for 3 consecutive days) after ischemic stroke (post‐onset), and in other experiments via administration of 4‐OHT (80 mg/kg in corn oil; Intraperitoneal; 1× daily for 5 consecutive days) prior to ischemic stroke (pre‐onset), to excise the flanked *loxP* sites, leading to KLF4 specific downregulation in PDGFR*β* ^+^ cells (Figure 1A). All mouse genotypes were confirmed by PCR using DNA isolated from ear punches. The following probes were used; For PDGFR*β* ^CreERT2^: Common: 5′‐CAC AAC TGA AGT AAG TTC CAC C‐3′; Wild type reverse: 5′‐GTC GAT GTG ACG GTGT TTC GA‐3′; Mutant reverse: 5′‐CGT TCT TGG ACT ACC TGT ACA‐3′. For Ai9: oIMR9020: Wild type forward: 5′‐AAG GGA GCT GCA GTG GAG TA‐3′; oIMR9021: Wild type reverse: 5′‐CCG AAA ATC TGT GGG AAG TC‐3′; oIMR9103: Mutant reverse: 5′‐GGC ATT AAA GCA GCG TAT CC‐3′; oIMR9105: Mutant forward: 5′‐CTG TTC CTG TAC GGC ATG G‐3′. For KLF4^Flox^: Exon 1: 5′‐CTG GGC CCC CAC ATT AAT GAG‐3′; Exon 2: 5′‐CGC TGA CAG CCA TGT CAG ACT‐3′ (PMID: 12015290; PMID: 15825076). All animal procedures and handling were performed according to the Canadian Council on Animal Care guidelines, as implemented by *Comité de Protection des Animaux de l'Université Laval‐3* (CPAUL‐3; Protocol # 20‐470). Animal studies were reported according to ARRIVE 2.0 guidelines.

### 2.2 Experimental Ischemic Stroke

Mice were subjected to focal ischemic stroke via the transient middle cerebral artery occlusion (MCAo) using the intraluminal filament technique (Manrique‐Castano et al. 2024). Mice were anesthetized with 3% isoflurane for induction and 1.5% for maintenance (95% O_2_, 2 L/min), and body temperature was maintained between 36°C and 37°C using a feedback‐controlled heating system (Harvard Apparatus, QC, Canada). A midline neck incision was performed, and the left common carotid artery (CCA) was exposed under a surgical microscope and ligated. A 7–0 silicon‐coated nylon monofilament (MCAO suture, Doccol Corporation, Cat# 7022910PK5Re) was inserted through the internal carotid artery (ICA) until the origin of MCA. The monofilament was left in place for 30 min and then withdrawn to mediate reperfusion (Figure 1A). This model generates a focal injury in the striatum and overlaying cortex as is associated with the formation of neatly demarcated astroglial and fibrotic scar. Brains were harvested at 3, 7, 14, and 30 days post‐ischemic stroke (DPI) for further analysis (Figure 1A). C57BL6/J, PDGFR*β* ^CreERT2^, KLF4^Flox^, and Ai9 sham‐operated animals with similar 4‐OHT regimen were generated and used whenever required as controls.

### 2.3 Brain Sectioning and Immunolabeling

Mice were euthanized via transcardial perfusion with ice‐cold phosphate‐buffered saline (PBS) followed by 4% paraformaldehyde (PFA). Brains were post‐fixed at 4°C by immersion in 4% paraformaldehyde (PFA) for 16 to 24 h. Cryoprotection was achieved through sequential immersion in 15% and then 30% sucrose in PBS and were subsequently frozen using dry ice and stored at −80°C until sectioning using a freezing microtome. We generated 25 μm‐thick coronal sections and conducted staining either on‐slide or free‐floating, depending on the experimental requirements. We share the detailed staining protocols and corresponding antibodies/reagents in the “Protocols” component of the OSF repository.

### 2.4 Fluorescent in Situ Hybridization (FISH)

Fluorescence in situ hybridization (FISH) was performed to assess PDGFR*β* mRNA expression using the RNAscope Fluorescent Multiplex Reagent Kit (ACDBIO, Cat #323100), strictly following the manufacturer's protocol. Free‐floating brain sections were mounted onto the Superfrost Plus slide and kept at −20°C to dry for 1 h before being baked for 30 min at 60°C. The sections were fixed for 15 min in 4% PFA and washed with 0.1 M PBS. Sections were next dehydrated using ethanol and incubated in hydrogen peroxidase (H_2_O_2_) for 10 min. Following retrieval, sections were immersed in a 100% ethanol bath, a hydrophobic barrier was drawn, and 5 drops of Protease III cocktail were added to completely cover the sections for 40 min at room temperature (RT). Following a series of washes with 0.1 M PBS, 4 drops of mouse RNAscope Probe‐*PDGFRβ*‐C1 (411381) were applied to entirely cover sections, which were immediately placed in the humidity control tray of the HybEZ Oven for 2 h at 40°C. Sections were gently removed, liquid excess was removed, and were next rinsed with 1× wash buffer. For hybridization, 4 drops of Amp‐1 were added to entirely cover the sections, and were incubated in the HybEZ Oven for 30 min at 40°C. This process was repeated with Amp‐2 for 30 min and Amp‐3 for 15 min. PDGFR*β* mRNA transcripts were detected using Opal 690 (1/700), and 4 drops of DAPI (provided in the kit) were added to sections and kept for 30 s, followed by a series of washes with 1× wash buffer. Slides were next mounted with 110 μL of Fluoromount‐G anti‐fade medium, carefully cover‐slipped, and placed at 4°C in the dark until analysis. Epifluorescence images were acquired using an Axio Observer microscope equipped with a module for optical sectioning (Apotome.2) and Axiocam 503 monochrome camera, and processed using ZEN Imaging Software (Carl Zeiss Canada, ON, Canada).

### 2.5 Brain Section Imaging

Brain sections were imaged using either widefield Zeiss Axio Observer or laser scan confocal Zeiss (LSM800) microscope with adequate objectives. Due to strong recombination activity, tdTomato signal leaked into the green channel (488 nm) when using widefield microscopy. Consequently, we exclusively used laser scan confocal microscopy to image the green channel, employing appropriate excitation wavelengths and detector ranges (see corresponding *readme* files). We share widefield images in (.tif or .czi) formats to encourage reuse and validation of our image sets. Technical metadata, including imaging parameters and *readme* files, are incorporated in the (.zip) files. The “Images_Low‐Resolution” component of the OSF repository contains complete sets of low‐resolution images used for illustration. Brain sections were consistently imaged at the level of MCA territory (bregma 0.44 to −0.06 mm) (Figure 1A) to acquire images of whole‐brain, ipsilateral, or regions of interest (ROIs) at different magnifications, as specified in supplementary methods and results sections. To facilitate cell detection, we used tailored batch‐processing FIJI (ImageJ v1.54f) scripts (.ijm) to handle and enhance images (Schindelin et al. 2012). The scripts are available in each .zip file at the Zenodo repositories and in the “Images_ Processing” component of the OSF repository.

### 2.6 Unbiased Cell Detection, Quantification, and Colocalization

Unbiased and automated cell detection, quantification, and colocalization analysis was applied using QuPath (v 0.4.4) (Bankhead et al. 2017) and CellProfiler (v.4.2.4) (Schindelin et al. 2012; Stirling et al. 2021). When necessary, machine learning‐based pixel classification was employed to obtain segmentation maps using Ilastik (Berg et al. 2019). We provide detailed descriptions for each image set in the supplementary methods and share the pipeline files/scripts in “Images_Processing” component of the OSF repository for validation and reproducibility of our results.

### 2.7 Morphological Analysis of PDGFR*β* ^+^ Cells and Principal Component Analysis

Z‐stack images of PDGFR*β* ^tdTtomato^/CD31‐ and CD13‐immunolabeled sections were acquired using a laser scan confocal microscope at 40× (see immunolabeling protocol) to analyze the morphology and spatial location of PDGFR*β* ^tdTomato^ cells in defined ROIs, specifically, the injured cortex, the injured striatum, and the healthy cortical peri‐lesional regions (see QN; Supplementary Method 10). Using FIJI, z‐projection images were generated and manually segmented into one or more well‐identified cells per ROI. Next, Python libraries skimage (van der Walt et al. 2014) and scipy (Virtanen et al. 2020) were used to enhance, threshold, and generate masks of individual cells. The various objects were imported to CellProfiler to perform a user‐defined classification into (i) amoeboid, (ii) perivascular, (iii) reticuloparenchymal, (iv) reticulite morphological categories. The classification results were subsequently imported into R to conduct principal component analysis (PCA) using the FactoMineR and factoextra packages (see Supplementary Method 10).

### 2.8 Fluorescence‐Activated Cell Sorting (FACS)

Brain PDGFR*β* ^tdTomato^ cells from PDGFR*β* ^tdTomato^ reporter mice were isolated via FACS at 14 DPI using a modified protocol of adult brain dissociation kit (Miltenyi Biotec, cat # 130‐107‐677), as detailed in (Protocol; see Supplementary Method 8). Data were visualized and analyzed using open‐source Bioconductor packages including flowCore (Hahne et al. 2009) and ggcyto (Van et al. 2018). Quality checks were performed using the flowAI package (Monaco et al. 2016). The original “.fcs files”, along with compensation controls and quality checks files, are available in “Datasets/FACS” component of the OSF repository. FACS files were processed using quarto notebook running R version 4.4, while the modeling with the brms package was conducted in R 4.1.2.

### 2.9 Data Analysis

Daya was analyzed using open‐source notebooks and scripts in Quarto (QN) (R language), and Jupyter (Python language) both within R‐studio IDE environment. The analysis strategy involved, (i) generating Bayesian posterior distributions via Markov chain Monte Carlo (MCMC) methods (Kruschke and Liddell 2018) using the brms package (Bürkner 2017); (ii) performing PPA with the spatstat package (Baddeley and Turner 2005); (iii) conducting TDA (Bhaskar et al. 2021) using Ghudi (The GUDHI Project 2023) and Ripser (Bauer 2021); and (iv) applying bootstrapping techniques (Chernik 2007) using SciPy (Virtanen et al. 2020). The versions of packages and libraries are indicated in the sessionInfo of each notebook. We performed scientific inference based on parameter and coefficient values and their uncertainty. Annotated QNs and JNs are shared in our GitHub repository. These resources contain raw data handling, exploratory data analysis, statistical modeling, model comparison and diagnostics, and analysis and visualization of results. Below, we specify common aspects of our data analysis approaches.

The primary approach for data analysis involves MCMC‐based Bayesian modeling using the brms package (Bürkner 2017). We explored the parameter space and derived stable posterior distributions using the following standard arguments: seed = 8807, chains = 4, cores = 4, warmup = 2500, iter = 5000, control = list(adapt_delta = 0.99, max_treedepth = 15). In each QN, we specify priors, model formulas, family distributions, and handling of research outputs. We share the fitted statistical models as (.rds) (R) objects in the “Datasets/StatisticalModels_brms” OSF repository component, and result tables as (.tex or .html) files in the “Tables” component. Model summaries are available in supplementary methods in GitHub. We report the mean parameter, or coefficient estimates with their 95% credible intervals (CI) (Hespanhol et al. 2019). The scale of the coefficients may vary (link function ≠ identity) depending on the family distribution used to fit the model. For model diagnostics, we plotted the model predictions against data using the (*pp_check*) function from (brms), and performed model evaluation/selection based on Watanabe‐Akaike Information Criterion (WAIC) or Leave‐one‐out (Loo) cross‐validation using the loo package (Vehtari et al. 2017).

For visualization of Bayesian models, we use ggplot (Wickham 2016) and ggdist (Kay 2024) packages, or the *conditional_effects* function from brms. We share the source code in each QN and the individual plots in the “Communication/Plots” component of the OSF repository. Broadly, the results are presented using *stat_halfeye* from ggdist to depict probability densities or as point estimates from *conditional_effects* with 95% CI represented as whiskers. When pertinent, we calculated pairwise contrasts of interest using the emmeans package (Searle et al. 1980). These comparisons are presented as half‐eye probability densities and point interval ranges, together with a Region of Practical Equivalence (ROPE) depicting the posterior density that falls within the error variance (Makowski et al. 2019).

### 2.10 Point Pattern Analysis (PPA)

We employed the spatstat package (Baddeley and Turner 2005) and its associated dependencies to analyze the spatial arrangement of cells through PPA. Using functions within spatstat, we created point patterns (ppp), calculated density kernels (density), estimated relative distributions (rhohat), and fitted multiple point process models (mppm). The point patterns as (.rds) hyperframes files are shared in the “Datasets/PointPatterns” component of the OSF repository. We used the base plotting system in R to visualize the graphical results and tables from PPA. For density kernels, the topo .colors (256) color scale was applied tailored to each data set (refer to QNs and figure legends). We plotted the relative distributions as the pooled (per group) mean (displayed in magenta) with two‐sigma confidence intervals represented by a gray‐shaded region. Additionally, we depicted the pooled mean intensity as a *cyan‐dashed line*, along with the corresponding lower (*black‐*solid line) and upper (*green‐dotted line*) limits of the two‐sigma confidence intervals. We note that the scales of the plots were adjusted or constrained to facilitate visualization (see QNs and figure legends). Since the mppm spatial models utilize a Poisson distribution, the summary tables in the QNs are presented on the log scale.

### 2.11 Topological Data Analysis (TDA)

We used the Ghudi and Ripser Python libraries to extract topological features from cell‐derived point clouds, which we share as (.npy) files in the “Datasets/PointClouds” component of the OSF repository. To create Vietoris‐Rips complexes, we utilized gd.RipsComplex with specific filtration values (see JNs; Supplementary Method 13). We share sets of static and animated Vietoris‐Rips complexes in the “Communication/Plots” OSF component. The dimension‐0 and dimension‐1 persistent homology (Otter et al. 2017) were then calculated using ripser, for which the corresponding persistence diagrams are shared as .png plots or .npy objects. Using these persistent diagrams, we computed Betti curves and compared the topological features of PDGFR*β* ^+^ cells among different time points within the specific ROIs. To obtain point estimates and 95% confidence intervals, we bootstrapped (1000 iterations) (Chernik 2007) the median bottleneck (bottleneck_distance) and Wasserstein (wasserstein_distance) distances. These results are presented as heat maps displaying median distances along with their corresponding 95% CI.

## 3 Results

### 3.1 Astrocytes and PDGFR*β* ^+^ Cells Respond Differently to Ischemic Stroke

To establish quantitative parameters at baseline of the macroscopic tissue architecture for our spatial models, we first imaged the complete ischemic hemisphere to analyze the ratio of brain shrinkage (atrophy) in mice subjected to experimental stroke (Figure 2A; see Supplementary Method 1). Our analysis estimates that from 7 to ~20 DPI, shrinkage proceeds at a rate of 0.34 mm^2^ per day [7 DPI = 14 mm^2^ ± 0.4; 30 DPI = 7.6 mm^2^ ± 0.4] (QN; Figures 2B and S1D,E; Table S1A,B). This represents a *~*46% reduction in hemispheric volume, which accounts for a substantial loss of the cortical mass and the gradual impairment of the cytoarchitectural division between the cortex and striatum at the level of corpus callosum. Within this highly dynamic microenvironment, we assessed the reactivity of PDGFR*β* ^+^ cells alongside GFAP^+^ astrocytes in PDGFR*β* ^tdTomato^ reporter mice by quantifying changes in integrated density (IntDen) (see Supplementary Method 2) over the progression course of brain injury (Figures 2C and S1A). Given the computational limitations in deriving absolute counts of highly ramified and overlapping cells within dense and compacted structures, such as scars (further detailed in the discussion), IntDen is used herein as a composite metric reflecting both local cell accumulation as well as reactivity.

**FIGURE 2:**
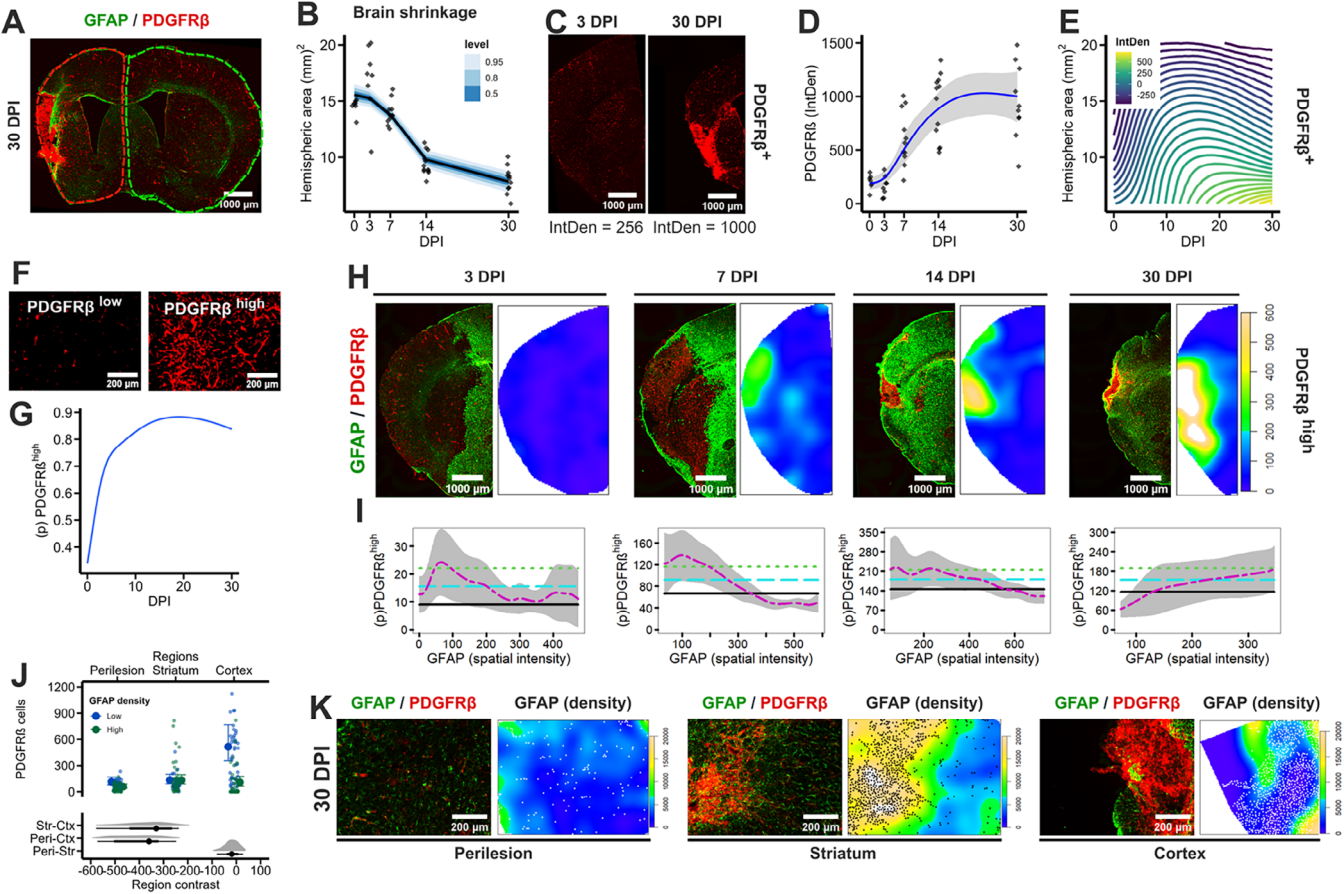
PDGFR*β* ^+^ cells actively respond to injury after ischemic stroke to partake in the formation of an inner scar compartment. (A) Representative brain section (stitched, 5× magnification) at 30 DPI showing the measured ipsilateral (red outline) and contralateral (green outline) hemispheres. (B) Fitted splines model for hemispheric area ~s(DPI). The stat_lineribbon() is displayed with the mean hemispheric area (black) and different credible intervals: 0.05, 0.8, 0.95 (blue brewer scale) and observations as diamonds (see Figure S1D,E (left); Table S1; source data, code, and plot). (C) Representative brain sections (stitched, 5× magnification) at 3 and 30 DPI showing PDGFR*β* ^tdTomato^ cells (see image set). Integrated density (IntDen) values at the button are shown as a reference. (D) Fitted splines model for PDGFR*β*_IntDen_ *~*s(DPI). The conditional_effects are displayed with the mean (blue) and 95% CI (gray‐shadowed region). The observations are displayed as black diamonds (see Figure S1D,E (middle); Table S2; source data, code, and plot). (E) Fitted splines model for PDGFR*β*_IntDen_ *~*t2(DPI, Area). The conditional_effects are displayed with a Viridis scale (IntDen) (see Table S3; source data, code, and plot). (F) Sample crops of PDGFR*β* ^low^ (non‐reactive) and PDGFR*β* ^high^ (reactive) cells in the ischemic hemisphere. (G) Fitted binomial model for PDGFR*β* ^high^|PDGFR*β* ^total^ *~*s(DPI). The conditional_effects are displayed with mean (blue) and 95% CI (gray‐shadowed region) (see Table S6; source data, code, and plot). (H) Representative GFAP^+^/PDGFR*β* ^tdTomato^ immunolabeling in brain sections (left) (stitched, 5× magnification) with corresponding GFAP density kernels (right) at 3, 7, 14, and 30 DPI. The density kernels are represented using the topo.colors (256) scale (see image set; point patterns, and density kernels available in the QN). (I) Relative distribution diagrams (rhohat) depicting the probability of PDGFR*β* ^high^ cells allocation (y‐axis) relative to GFAP spatial intensity (*x*‐axis). The pooled mean intensity (*cyan‐dashed line*) is shown with their corresponding lower (*black‐*solid line) and upper (*green‐dotted line*) limits of the two‐sigma confidence intervals. Note the dissimilar *xy* axes limited by the mapping of the rhohat function for each DPI (see source point patterns, code). The raw plots are available in the “Communication/Plots” component of the OSF repository. (J) Fitted model for PDGFR*β* ^high^ *~*Region:GFAP. The conditional_effects are displayed with mean cell counts and 95% CI as whiskers. At the bottom, entire posterior densities for the contrast between regions are shown with stat_pointinterval (see Figure S2F; Table S8; source data, code, and plot). (K) Representative GFAP^+^/PDGFR*β* ^tdTomato^ immunolabeling in brain sections (left) (10× magnification) with corresponding GFAP density kernels (right) at 30 DPI. The density kernels are represented using the topo.colors (256) scale and PDGFR*β* ^tdTomato^ cells as black or white dots (see image set; point patterns, and density kernels available in the QN). High‐resolution figure: https://osf.io/jxb4z/files/m9g8v.

Analysis of PDGFR*β* ^+^ cells (QN; Table S2A,B) yields a non‐linear increase in the IntDen until the 3rd week after ischemic stroke [0–23 DPI = 841, *β*1 = 36], followed by a nearly plateau phase [23–30 DPI = −29, *β*1 = −4.4] (Figures 2D and S1D,E). We found that reactive PDGFR*β* ^+^ cells are mainly located outside the envelope formed by GFAP^+^ cells, a region prone to liquefaction. In PDGFR*β* ^tdTomato^ cells, overall tdTomato intensity is proportional to basal enrichment of PDGFR*β* that drives Cre‐recombinase expression in cells. Notably, by conditioning IntDen to the hemispheric area (Figure 2E; Table S3), our model predicts that tdTomato IntDen increases in small hemispheric volumes, suggesting that reactivity of PDGFR*β* ^+^ cells implicates more efficient accumulation of tdTomato. An analogous analysis for GFAP^+^ cells (QN) reveals a similar increase in the IntDen until the 2nd week after ischemic stroke [0–16 DPI = +835, *β*1 = 50.1], followed by a resolution phase [16 to 30 DPI = −480, *β*1 = −36] (Figure S1A–E; Table S4A,B). Notably, when conditioning to the hemispheric area, our model predicts an increased reactivity of GFAP^+^ cells at 30 DPI under non‐shrinking conditions (QN; Figure S1C; Table S5). This implies that brain atrophy upon stroke favors emergence of reactive PDGFR*β* ^+^ cells and does not substantially affect the distribution of reactive GFAP^+^ cells.

### 3.2 Highly Reactive PDGFR*β* ^+^ Cells Aggregate in the Inner Scar Compartment

Building upon our volumetric baseline assessments, we employed PPA to establish quantitative spatial parameters for the localization of reactive PDGFR*β* ^+^ cells relative to the GFAP^+^ astrocytic boundary (QN, Supplementary Method 4). Using machine learning‐based classifiers in QuPath, we segregated PDGFR*β* ^high^ (highly reactive; high tdTomato intensity = potent Cre‐mediated recombination driven by high basal PDGFR*β* expression) and PDGFR*β* ^low^ (quiescent; low tdTomato intensity = less potent Cre‐mediated recombination driven by low basal PDGFR*β* expression) cell populations (QN; Supplementary Method 3). Our predictive models outline an increasing likelihood of PDGFR*β* ^high^ cell emergence [*β*_1_ = 25 ± 0.85] within the lesioned areas up to the 2nd week after ischemic stroke [~80%–90%] (Figure 2F,G; Table S6). Spatial modeling via PPA (*rhohat* and *mppm*) reveals that PDGFR*β* ^high^ cells cluster predictably outside the envelope formed by reactive GFAP^+^ cells (convex hull) during the acute/subacute phases of ischemic stroke [*β*_1_ = −0.0015 (3D); −0.0018 (7D); −0.00067 (14D)]. By 30 DPI, we observe a moderate overlap between both cell populations [*β*_1_ = 0.0017] (see Figure 2H,I; QN; Supplementary Method 4), attributed to progressive brain atrophy and subsequent structural collapse of boundaries between striatum and overlaying cortex. Conversely, perivascular PDGFR*β* ^low^ cells remain predominantly co‐localized within the envelope occupied by GFAP^+^ astrocytes (Figure S1F and QN). To mathematically validate this structural demarcation, we modeled the establishment of GFAP^+^ convex hull across whole brain sections (QN; Supplementary Method 5; Figure S1G). Our estimates confirm that glial scar borders form within the 1st week post‐injury and remain stable over time [*β*_1_ = −0.10, 95% CI = −0.38–0.18] (Figure S1G,H; Table S7). Our parameterized models indicate that highly reactive PDGFR*β* ^+^ cells are potently localized early to injured regions prone to irreversible degeneration, a compartmentalization robustly supported by cell count analysis in GFAP^low^ and GFAP^high^ density‐defined tessellations (QN, Supplementary Method 6). Indeed, we observe a ~3‐fold change [*β*_cortex_ = exp.(1.06) = 2.89, 95% CI = 1.9–4.3] in the value for PDGFR*β* ^high^ cells in GFAP^low^ areas in the injured cortex compared to the healthy peri‐lesional region, which decreases by a factor of exp.(−0.64) = −1.89, 95% CI −3.32 to −1.06 for GFAP^high^ areas in the same regions (Figure 2J,K). The QN, Table S8 and Figure S2E,F portrait estimates by region over injury progression (DPI).

Next, to test whether these spatial parameters depend upon injury severity, we modeled a mild striatal‐specific injury (QN, Supplementary Method 6), which lacks important brain atrophy and substantial cortical damage. Under these conditions, our Bayesian estimates reveal that the probability of PDGFR*β* ^high^ cell emergence is reduced by approximately one half compared to the severe cortico‐striatal injury (~0.90 vs. 0.45) (Figure S2A,B; Table S9). Importantly, the distinct spatial segregation between PDGFR*β* ^high^ and PDGFR*β* ^low^ cell populations observed in the severe brain injuries is absent in mild brain injuries (Figure S2C,D). These models quantitatively confirm that the robust aggregation and segregation of PDGFR*β* ^high^ cells represent a specific micro‐architectural signature associated with irreversible tissue damage, although structure‐specific baseline reactivities cannot be entirely excluded.

### 3.3 Morphologically Distinct PDGFR*β* ^+^ Cells Are Present at the Injured Tissue

To quantitatively assess the currently proposed paradigm of vascular detachment of PDGFR*β* ^+^ cells established via antibody‐based approaches, we employed our spatial models in PDGFR*β* ^tdTomato^ reporter mice to accurately track perivascular versus parenchymal (i.e., non‐vascular associated) PDGFR*β* ^+^ cell populations relative to the interaction with CD31^+^ brain vasculature (Figure 3A, QN, Supplementary Method 7). Our analysis shows that in the healthy peri‐lesional region, the probability of parenchymal non‐vascular associated PDGFR*β* ^+^ cells ranges only from 3% to 9%. This value peaks at ~25%, in the injured striatum and to ~50% in the injured cortex during the 2nd week after ischemic stroke (Figure 3B,C; Table S10C). We display cell counts and the contrast between injured cortex and injured striatum (Figure S3A,B). Next, we mathematically modeled the decreased probability of parenchymal PDGFR*β* ^+^ cell emergence in severely injured regions as a function of progressive cell loss associated with tissue degeneration. To quantitatively validate this trajectory, we utilized fluorescence‐activated cell sorting (FACS) to measure the extent of cell death in all PDGFR*β* ^+^ cells (Figure 3D–G; Figure S4; Supplementary Method 8). The resulting estimates indicate a likely increase at 14 DPI for the total proportion of PDGFR*β* ^+^ cells [*β*_1_ = 0.074, 95% CI = 0.033–0.114], as well as the dead PDGFR*β* ^+^ cell fraction [*β*_1_ = 0.137, 95% CI = −0.006 to 0.299] (see Table S11A,B). Our parameterized data suggest reactive PDGFR*β* ^+^ cells populating the core are highly susceptible to death. Afterwards, we parameterized the parenchymal emergence of PDGFR*β* ^+^ cells in mild striatal‐specific injury at 14 and 30 DPI to isolate the effect of injury severity on cell reactivity dynamics (see Supplementary Method 7). Upon mild brain injury, the probability of parenchymal PDGFR*β* ^+^ cells exhibits a continuous upward trend (Figure S3E; Table S12), structurally contrasting with the decline observed upon severe cortico‐striatal injury, even as absolute cell counts rise across both severities (Figure S3C,D; Table S13). These divergent trajectories indicate that extent of PDGFR*β* ^+^ cell loss directly correlates with injury severity after stroke. The possible contribution of tdTomato expression in quiescent parenchymal PDGFR*β* ^low^ cells upon Cre‐recombination activation for long period in PDGFR*β* ^tdTomato^ reporter mouse model was ruled out (see Supplementary Method 7). This confirms that the observed increased cell density at the lesion site after stroke genuinely translates emergence of new parenchymal reactive PDGFR*β* ^high^ cells (Figure S3F,G; Table S14).

**FIGURE 3:**
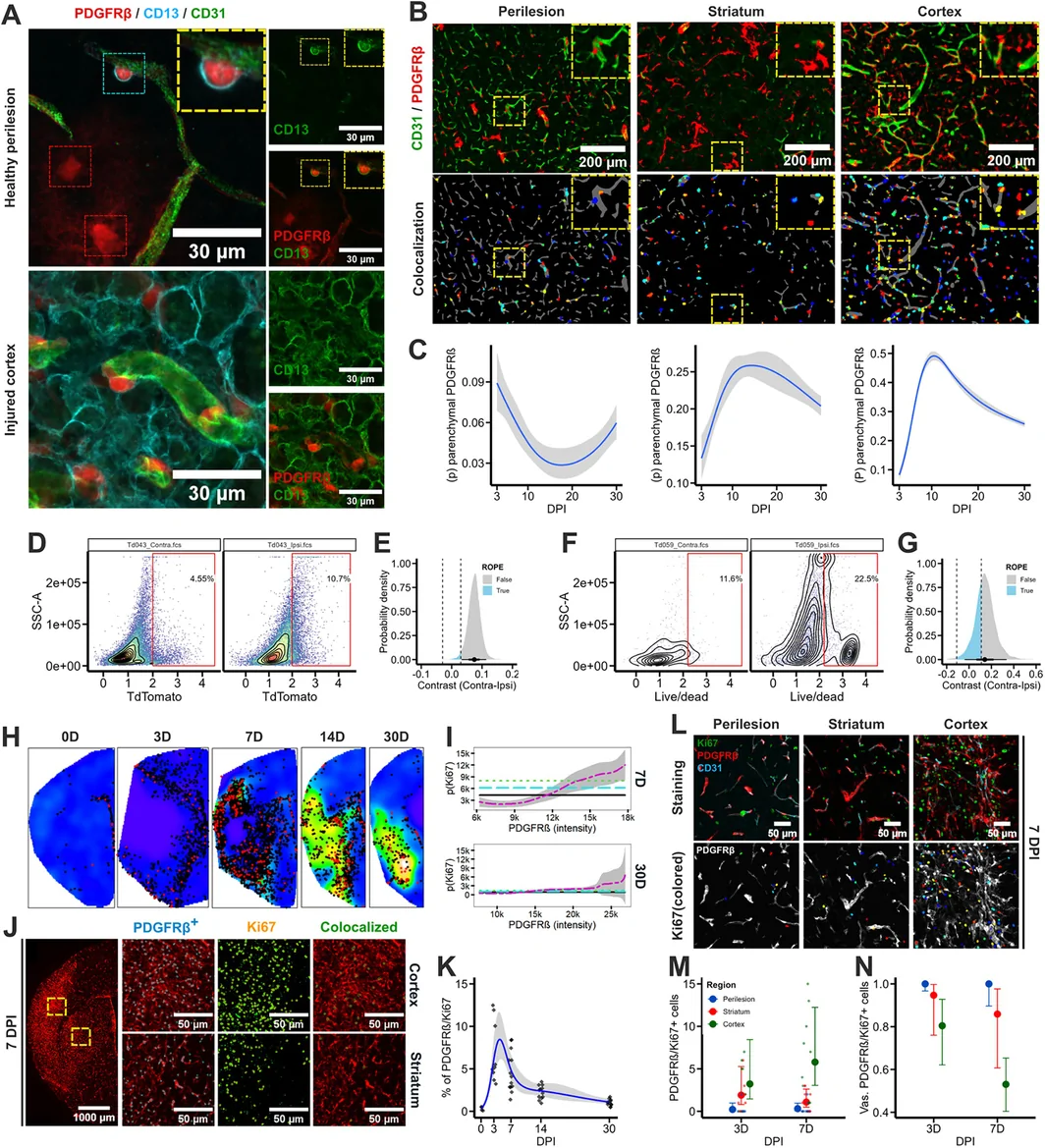
Reactive PDGFR*β* ^+^ cells comprise perivascular and parenchymal cells. (A) Representative images (40× magnification) of PDGFR*β* ^+^ cells in the healthy peri‐lesional and injured cortex. PDGFR*β* ^tdTomato^ cells are associated with CD31^+^ vasculature, and their nuclei co‐localize with CD13, a common pericyte marker. Parenchymal PDGFR*β* ^tdTomato^/CD13^−^ cells are visible interacting with the CD31^+^ vasculature through fine ramifications. In the injured cortex, nuclei‐based colocalization with CD13 is lost and parenchymal PDGFR*β* ^tdTomato^ cells are not in place (see the entire image set). (B) Representative images (10× magnification) of PDGFR*β* ^tdTomato^/CD31^+^ immunolabeling (upper row), and co‐localization analysis (CellProfiler) of PDGFR*β* ^tdTomato^ nuclei (colored) and CD31^+^ vasculature (gray) (bottom row) for peri‐lesional region, striatum, and cortex. The lined‐yellow box is zoomed in the upper right corner of each image (see the entire image set). (C) Fitted binomial models per ROIs for PDGFR*β* ^colocalized^|PDGFR*β* ^total^ *~*s(DPI). The conditional_effects are displayed with a mean (blue) and 95% CI (gray‐shadowed region) (see Figure S3A,B; Table S10; source data, code, and plots in the “Communication/Plots” component of the OSF repository. (D, F) Representative images for contralateral (left, contra) and ipsilateral (right, ipsi) hemispheres showing total PDGFR*β* ^tdTomato^ cells (D) and PDGFR*β* ^tdTomato^/Live Dead^+^ cells (F). In both cases, ggcyto is displayed with geom_density2d, geom_gate, and geom_stats and additional ggplot aesthetics (see Figure S4; source data in the “Datasets/FACS” component of the OSF, code, and plots in the “Communication/Plots/FACS_Pdgfrb” component of the OSF repository and). (E, G) Contrast between the percentage of PDGFR*β* ^tdTomato^ cells (E) and the percentage of PDGFR*β* ^tdTomato^ dead cells (G) in the contralateral (contra) and ipsilateral (ipsi) hemispheres for the linear model Cells~Hemisphere. Posterior draws (spread_draws) are shown with stat_halfeye() and ROPE (see Table S11; source data, code, and plots for (E, G). (H) Representative density kernels of PDGFR*β* ^tdTomato+^ cells at 0 (control), 3, 7, 14 and 30 DPI. The density kernels are represented using the topo.colors (256) scale, and KI67^+^ cells are represented as black dots and co‐localized PDGFR*β* ^tdTomato^/Ki67^+^ immunolabeling is red colored (see point patterns and density kernels in the QN). (I) Relative distribution diagrams (*rhohat*) depicting the probability of Ki67^+^ cell allocation (*y*‐axis) relative to PDGFR*β* ^tdTomato^ cell spatial intensity (x‐axis). The pooled mean intensity (*cyan‐dashed line*) is shown with their corresponding lower (*black‐*solid line) and upper (*green‐dotted line*) limits of the two‐sigma confidence intervals. Note the dissimilar *xy* axis limits defined by the point arguments mapped by the rhohat function for each DPI (see source point patterns, code, and plots for 7 and 30 DPI). (J) (left) Representative image (stitched 10× magnification) of PDGFR*β* ^tdTomato^/Ki67^+^ staining in brain section at 7 DPI. The upper (cortex) and bottom (striatum) panels show the boxed yellow regions, with overlays of detected (CellProfiler) PDGFR*β* ^tdTomato^ cells (cyan), Ki67 (orange), and co‐localized PDGFR*β* ^tdTomato^/Ki67^+^ cells (green) (see the entire image set). (K) Fitted splines model for Percentage*~*s(DPI, *k* = 5), family = lognormal. The conditional_effects are displayed with mean (blue) and 95% CI (gray‐shadowed region) and observations as diamonds (see Table S15 with complementary model derivatives; source data, code, and plot). (L) Representative images (20× magnification) of PDGFR*β* ^tdTomato^/CD31^+^/Ki67^+^ immunolabeling in brain sections (upper row) in the peri‐lesional region, the injured striatum and the injured cortex at 7 DPI. The bottom row depicts Ki67^+^ cells (colored) and PDGFR*β* ^tdTomato^ cells (gray) (see the entire image set). (M) Fitted model for the total number of colocalized PDGFR*β* ^tdTomato^/Ki67^+^ cells (co‐localized~DPI × Region, family = negbinomial). Posterior estimates are displayed with conditional_effects (mean + 95% CI). Dots are observations (see Table S16; source data, code, and plot). (N) Fitted model for the proportion of vascular PDGFR*β* ^tdTomato^/Ki67^+^ cells (Vascular|trials(total)*~*DPI × Region, family = binomial). Posterior estimates are displayed with conditional_effects (mean + 95% CI) (see Table S16; source data, code, and plot). High‐resolution figure: https://osf.io/jxb4z/files/s65pg.

### 3.4 Reactive PDGFR*β* ^+^ Cells Do Not Massively Proliferate Within the Injured Brain

Previous studies using antibodies targeting PDGFR*β* have reported important proliferation of PDGFR*β* ^+^ cells at the injured site during the 1st week of ischemic stroke as well as after traumatic brain injury (Özen et al. 2014; Zehendner et al. 2015). Based on these observations, we hypothesized that the emergence of PDGFR*β* ^+^ parenchymal cells may be due to local proliferation of resident cells rather than cell detachment from the vasculature, as previously suggested (Dore‐Duffy et al. 2000; Fernandez‐Klett et al. 2013; Menezes et al. 2020). Accordingly, we immunolabeled the proliferation marker Ki67 to analyze its distribution relative to the spatial intensity of PDGFR*β* ^tdTomato^ cells as well as its co‐localization with PDGFR*β* ^tdTomato^ cells (see Supplementary Methods 9 and 10). First, we observed consistent expression of Ki67 in regions of elevated tdTomato spatial intensity [*β*_0_ = 6.85, *β*_1_ = 7.26^e‐0^], particularly at 3 [*β*_1_ = 1.07^e‐04^] and 7 DPI [*β*1 = 7.05^e‐06^] (QN, Figure 3H,I). These findings align with the critical periods of cell proliferation associated with glial scar formation (Wanner et al. 2013; Xie et al. 2020). However, in contrast to what is reported in previous studies, our analysis revealed that the proportion of PDGFR*β* ^+^/Ki67^+^ cells (relative to the total number of PDGFR*β* ^+^ cells) does not exceed 10% at its peak at 3 DPI [*x̄* = 7.21%, 95% CI = 4.88–10.13] (see QN, Figure 3J,K; Table S15). These results occurred despite potential systemic overestimation of co‐localized PDGFR*β* ^+^/Ki67^+^ cells due to partial object overlap inherent in the applied automated/unbiased analysis approach (see Supplementary Method 9). We further analyzed PDGFR*β* ^+^/Ki67^+^ cells using laser scan confocal microscopy (20× magnification) along with the endothelial cell marker CD31 in defined ROIs (Figure 3L; QN; Supplementary Method 10). Our results reveal that only a small fraction of co‐localizing PDGFR*β* ^+^/Ki67^+^ cells (Figure 3M; Table S16A) is mainly derived from non‐vascular associated cells in the injured cortex and even to a lesser extent in the injured striatum (Figure 3N; Table S16B). Overall, our findings with the PDGFR*β* ^tdTomato^ reporter mouse suggest that reactive PDGFR*β* ^+^ cells do not massively proliferate after injury, nor could the small fraction of proliferative cells account for the substantial increase in the density of PDGFR*β* ^+^ cells observed at the injured site. These observations question the origin of the parenchymal proliferative PDGFR*β* ^+^ cells reported using antibody‐based approaches, which could originate from vascular detachment, as previously suggested, or from the recruitment of infiltrating PDGFR*β* ^+^ peripheral cells exhibiting fibrotic and macrophage properties, as previously reported by Sakuma et al. (2016), outlining new avenues in this research field.

### 3.5 Reactive PDGFR*β* ^+^ Cells Comprise Morphologically Diverse Populations

Using a semiautomated computational pipeline in Python (skimage and scipy), we analyzed the morphological characteristics and changes of reactive parenchymal and vascular‐associated PDGFR*β* ^+^ cells across injury progression after ischemic stroke (QN‐batch processing, QN‐analysis, Supplementary Method 11; Figure S5). We observed that the healthy peri‐lesional regions are associated with perivascular PDGFR*β* ^+^ cells that are abundantly co‐labeled with CD13, a marker that is expressed in pericytes under normal conditions and in a population of peripheral myeloid immune cells (Nguyen et al. 2023) (Figure 3A). In addition, our PDGFR*β* ^tdTomato^ reporter mouse revealed the presence of a subpopulation of quiescent PDGFR*β* ^low^ cells in the same region. These cells, hereafter referred to as reticuloparenchymal cells, exhibit a highly ramified morphology and maintain substantial narrow contacts with the vasculature via fine processes. Unlike perivascular cells, PDGFR*β* ^low^ cells do not express CD13, indicating that they are neither pericytes nor infiltrating immune cells. On the other hand, the injured brain contains PDGFR*β* ^high^ amoeboid cells, which may be either parenchymal or perivascular, as well as small ramified parenchymal cells, hereafter referred to as reticulite cells (Figure 4A). These reticulite cells are immunoreactive for CD13, suggesting a mural or myeloid immune identity. However, we could not confirm this aspect in amoeboid cells due to the strong immunoreactivity, cell density, and background noise at the injury core (Figure S5C). Based on PCA (QN‐analysis, Figures 4B and S5B; Supplementary Method 11), we portray PDGFR*β* ^+^ cells along with three distinct morphological features (Figures 4C and S5D). Note that due to the co‐linearity between the area and convex hull, the (data variable) area in Figure 4C is shown only as a reference; (1) perivascular PDGFR*β* ^+^ cells are anchored to CD31^+^ vasculature and exhibit a consistent branch length (0.73), convex hull (0.39), and intensity (0.31) (Figure 4C, red). In contrast, (2) reticuloparenchymal cells are distinguished by a larger convex hull (0.82) while their nuclei are located within the brain parenchyma and interact with CD31^+^ vasculature through fine ramifications (Figure 4C, green). Whereas (3) reticulites, located primarily in the ischemic striatum, are characterized by moderate intensity (0.006) relative to their branch length (−0.58), and convex hull (−0.66) (Figure 4C, cyan). Finally, (4) amoeboid PDGFR*β* ^+^ cells, prevalent in the ischemic cortex, show high PDGFR*β* activity (tdTomato relative intensity) (1.88) and are found both anchored to CD31^+^ vasculature as well as within the injured parenchyma (Figure 4C, magenta). Figure S5D portrays the probability of each cell subtype given the referred morphological traits. Our results underscore a strong phenotypic diversity of PDGFR*β* ^+^ cells that is amplified within in the injured brain.

**FIGURE 4:**
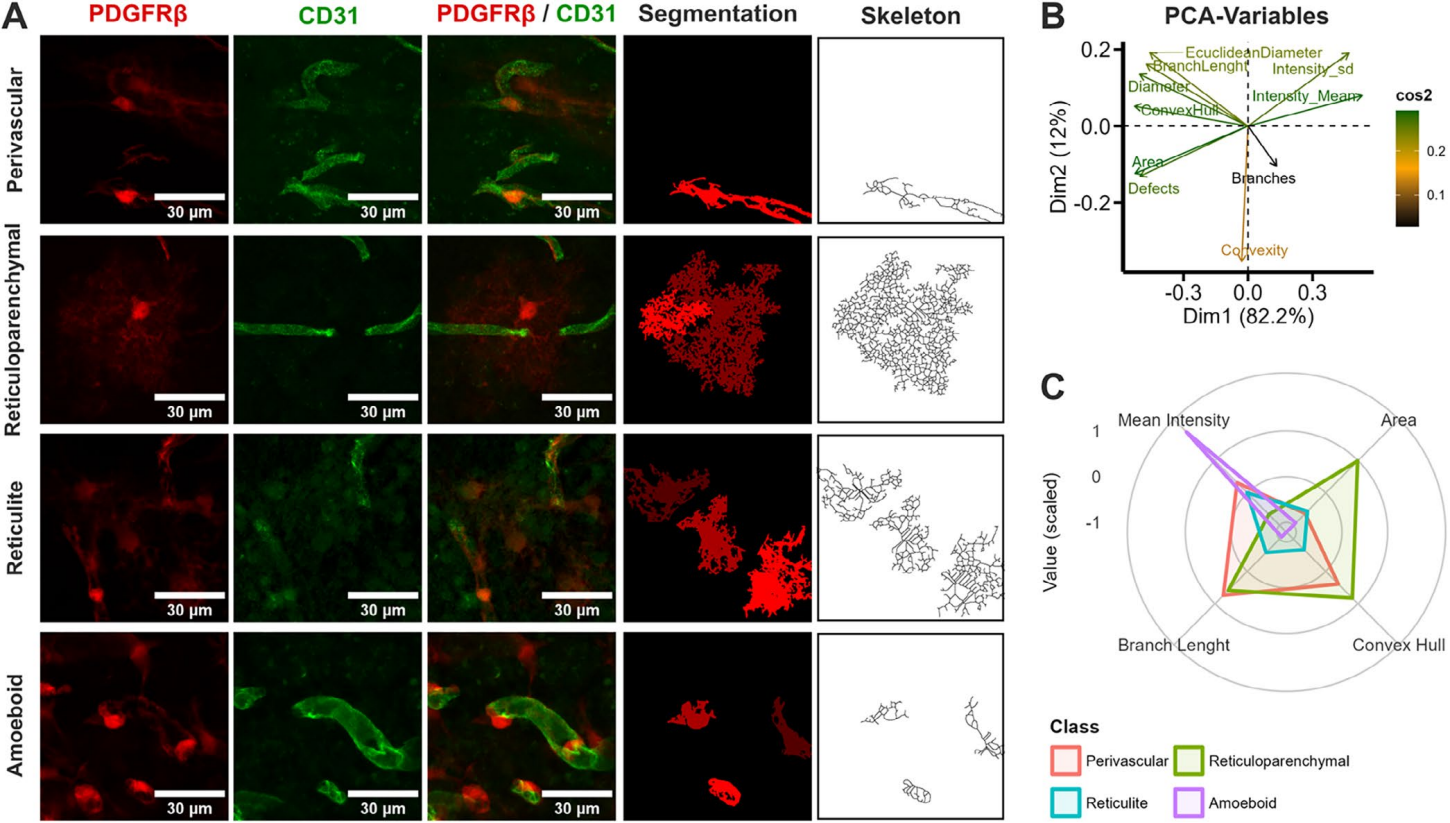
Morphological diversity of brain PDGFR*β* ^+^ cells. (A) Representative images (40× magnification) showing the morphological diversity of PDGFR*β* ^+^ cells. The healthy brain hosts perivascular PDGFR*β* ^high^ and PDGFR*β* ^low^ cells, whereas the injured areas showcase predominantly reticulite and amoeboid PDGFR*β* ^high^ cells (see Figure S5A; entire image set, cropped cells, and segmentations). (B) Biplot from the PCA analysis of morphological features of PDGFR*β* ^+^ cells. Variables negatively correlated are displayed on opposite sides of the Cartesian plane and its weight is shown as distance from the point of origin. Cos2 bar depicts the representation of each variable, with green as higher values (see Figure S5B; source data, code, and plot). (C) Spyder plot showing the three selected variables (mean intensity, convex hull, branch length; areas as reference) via PCA to characterize the morphology of PDGFR*β* ^+^ cells (see Figure S5C; source data, code, and plot). High‐resolution figure: https://osf.io/jxb4z/files/uv5cw.

During our investigations, we attempted to further characterize the morphologically distinct PDGFR*β* ^+^ cells, by co‐immunolabeling for CD13 (Figure S6A), GFAP (Figure S6B), IBA1 (Figure S6C), and PDGFR*α* (Figure S6C). However, the high cell density and the lack of well nuclei‐defined staining made the automated and unbiased co‐localization analysis impracticable. Nonetheless, we observed that perivascular PDGFR*β* ^+^ cells are immunoreactive to CD13 (R&D systems, cat # AF2335, RRID:AB_2227288 antibody), whereas reticuloparenchymal PDGFR*β* ^+^ cells in the healthy brain tissue are not (Figure S6A). In the injured regions, some GFAP‐stained cytoskeletons (Invitrogen, cat # 13‐0300, RRID:AB_86543 antibody) appeared to encircle the nuclei of PDGFR*β* ^+^ cells (Figure S6B). Under these conditions, asserting co‐localization is ambiguous because GFAP^+^ cell branches cannot be unbiasedly associated with specific nuclei. Similarly, we could not confirm co‐localization between PDGFR*β* ^+^ and IBA1^+^ cells (Wako, cat # 019‐19,741, RRID:AB_839504 antibody) as described by (Özen et al. 2014) due to increased cell density and staining artifacts introduced by the cell debris in the injured tissue. Moreover, PDGFR*α* immunolabeling (Abcam, cat # ab203491, RRID:AB_2,892,065) revealed that in the healthy brain, PDGFR*α* ^+^ cells and PDGFR*β* ^+^ cells constitute two distinct populations. PDGFR*α* ^+^ cells appeared as ramified parenchymal cells, whereas PDGFR*β* ^+^ cells are typical perivascular. This observation contrasts with previous reports using antibody‐based approaches to immunolabel PDGFR*β* with the Abcam antibody ab203491 (RRID:AB_2,892,065) (Menezes et al. 2020), which has claimed co‐localization between these two markers in perivascular PDGFR*β* ^+^ cells. Notably, Abcam recently reclassified this antibody as recognizing both PDGFR*α* as well as PDGFR*β*. Overall, we argue that further unbiased and reproducible investigations using simultaneous lineage tracking are needed.

### 3.6 Spatial and Topological Organization of Reactive PDGFR*β* ^+^ Cells Upon Injury

Next, we investigated the topological changes of reactive PDGFR*β* ^+^ cells in defined ROIs (peri‐lesion, striatum, and cortex) in the ipsilateral hemisphere using Haralick features (Supplementary Method 12) and TDA (Supplementary Method 13). The Haralick features (QN‐batch processing; QN‐analysis) were applied to understand image texture changes, as described by (Löfstedt et al. 2019). Our results by PCA (QN‐analysis; Figure S7A,B) revealed that no Haralick features have a strong weight for PDGFR*β* ^+^ cell reactivity. Given these results, we focused on evaluating the variations in contrast, entropy, and the inverse difference moment (IDM) (QN‐analysis and Supplementary Method 12). We found that increased IDM (homogeneity) is predominant in the different injured regions, especially the cortex [*β*_Ctx_ = 2.92, 95% CI = 1.13–4.83] (Figure 5A; Table S17), consistent with increased PDGFR*β* ^+^ cell‐occupied area [*β*_Ctx_ = 4.5, 95% CI = 2.4–6.4] (Figure S7C; Table S18). In contrast, the peri‐lesional region showed high local variation and more complex textures (entropy) (Figure 5A; Table S17). In Figure S7D and Table S17, we show the influence of entropy, contrast, and IDM on the classification of brain regions. Entropy appears to be the most discriminative attribute, forming a gradient from the peri‐lesional region (low entropy) towards the cortex (high entropy). Subsequently, we used TDA and persistence homology (Bhaskar et al. 2021) to elucidate the reorganization patterns of PDGFR*β* ^+^ cells in the same brain regions (JN; Supplementary Method 13). In Figure 5B, we illustrate representative Vietoris‐Rips complexes showing cell connectivity at a specific filtration value (200) (see OSF repository for the entire image set and animations for different filtration values, also shown in Video S1). We computed persistent homology (Figure S7E; see the full image set) and derived 0 and 1‐dimensional Betti curves to quantify the topological complexity of PDGFR*β* ^+^ cells over time (Figure 5C). We then calculated Wasserstein (Figure 5D) and bottleneck (Figure S7F) distances to represent the changes between DPI (Table S19). Our analysis of the number of connected components (0‐dimensional homology) revealed a consistent merging pattern in the peri‐lesional region at most time points (Figure 5C, upper row). At 30 DPI (left‐yellow line), however, we observed a higher merging at lower filtration values (~120 filtration value). This suggests that the healthy peri‐lesional region experiences small‐scale clustering at this specific timepoint. It is consistent with the features of 2‐dimensional loops (1‐dimensional homology), where components tended to form discrete loops rather than a continuous monolayer of cells (Figure 5C, left‐yellow line). This suggests a lack of homogeneity (see Haralick features) of PDGFR*β* ^+^ cells in the healthy brain tissue.

**FIGURE 5:**
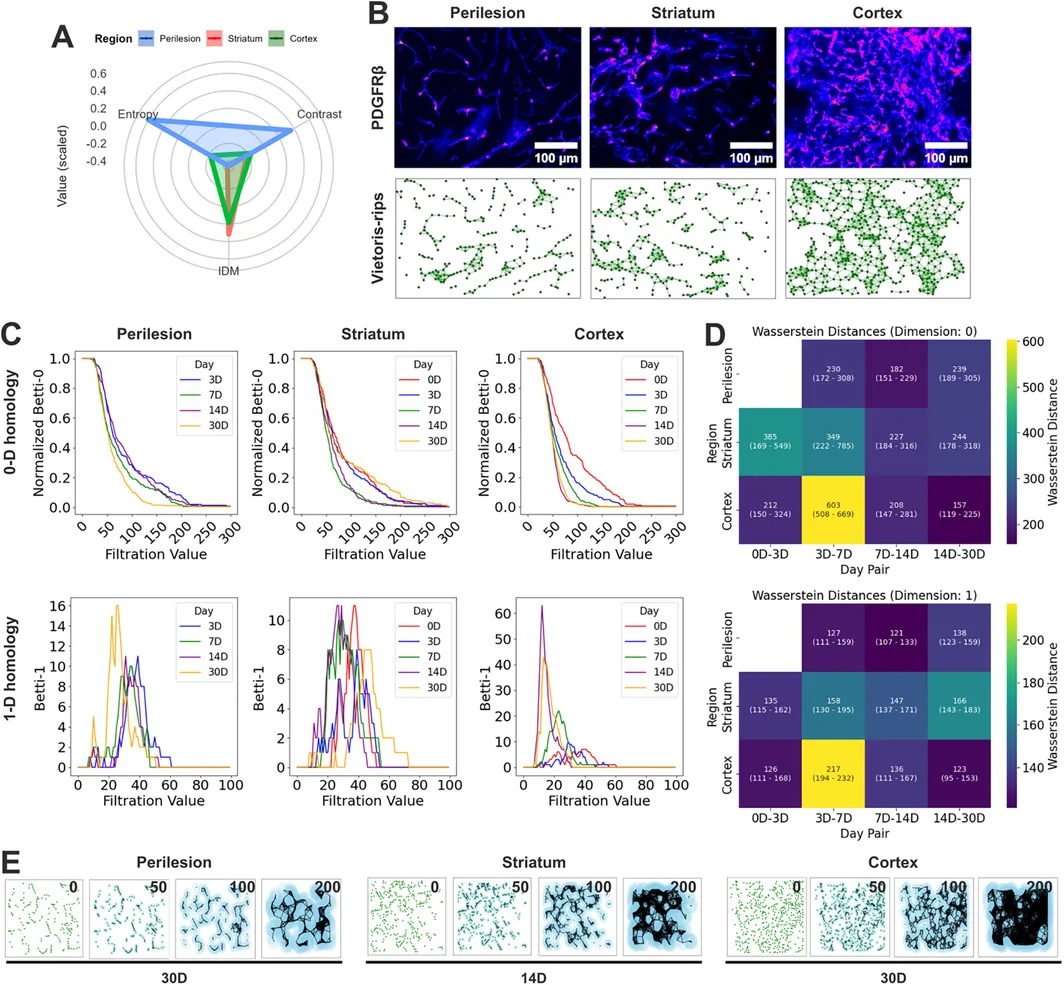
Haralick features and topological data analysis (TDA) of PDGFR*β* ^+^ cells. (A) Spyder plot showing the three selected variables (Entropy, contrast, IDM) by PCA to characterize the Haralick variables of PDGFR*β* ^+^ cells. The represented regions (peri‐lesional region, striatum, cortex) comprise animals from all the time points (see Figure S7A–C; Table S17; source data, code, and plot). (B) (upper row) Representative images (‘fire’ LUT) of the healthy peri‐lesional region, the injured striatum and the injured cortex. Magenta represents higher PDGFR*β* (tdTomato) promoter activity (see entire image set). (Low row) corresponding vietoris‐rips complexes, showing the topological complexity of cells (see source data, entire image set, different filtration values, and animated vietoris‐rips complexes). (C, D) Mean Betti curves (C) per time point and corresponding Wasserstein distances (D) between pairs of time points calculated from 0 and 1‐dimensional homology persistent diagrams (Figure S7E). The uncertainty in Wassertains distances was calculated using bootstrapping with 1000 iterations (see Table S19C,D). Betti curves for 0‐dimensional homology are normalized to 1 (1‐dimensional graphs are not normalized). Line plots (C) and heatmaps (D) are shown using matplotlib (see source point couds, persistent diagrams, and code). Note that bottleneck distances are portrayed in Figure S7F. The raw plots are available in the “Communication/Plots/Widefield_20x_ROIs_Pdgfrb_TDA” component of the OSF repository. (E) Representative images of point clouds derived from PDGFR*β* ^+^ cells at different filtration values (0, 50, 100, 200) for the healthy peri‐lesional region, the injured striatum, and the injured cortex (see entire image set, source data, and code). High‐resolution figure: https://osf.io/jxb4z/files/mdre6.

**FIGURE 6:**
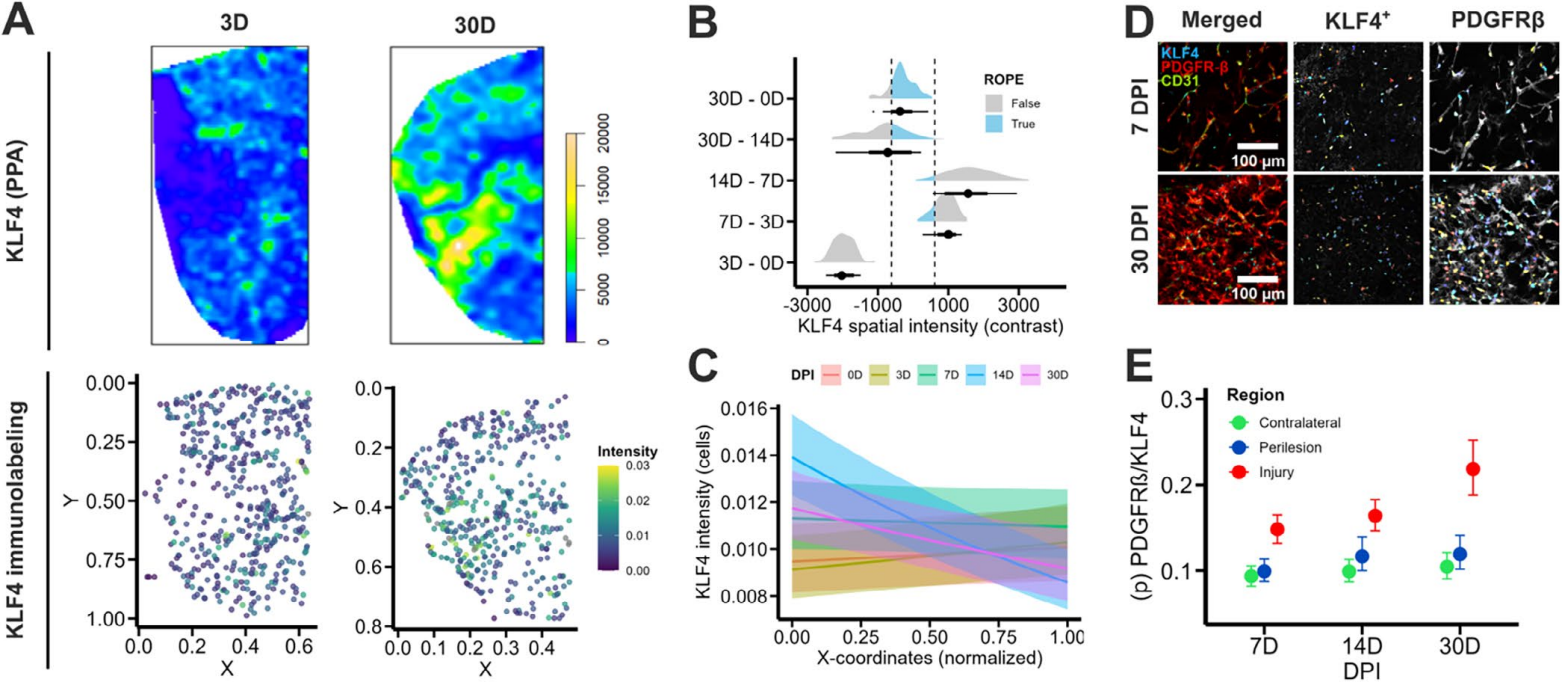
KLF4 overall spatiotemporal regulation after ischemic stroke. (A) (Upper row) Representative density kernels for KLF4^+^ cells (stitched, 10× magnification) in the ipsilateral hemisphere. The plots are represented using the topo.colors (256) scale (see QN and point patterns). (Bottom row) Representative plots of single cell KLF4 immunolabeling intensity. Coordinates at 0.0 are the dorsolateral cortex and 0.4 close to the ventricle. The plot is shown using geom_point and scale_color_viridis_c (see entire image set). (B) Fitted model for Intensity ~0 + DPI, sigma ~0 + DPI, family = student. The posterior probability of contrasts (emmeans) between time points of interest is displayed using add_predicted_draws with stat_halfeye. The ROPE is represented with the shadowed blue region (see Figure S8B; Table S20; source data, code, and plot). (C) Fitted model for Intensity ~0 + DPI × CenterX + (1|MouseID), family = lognormal. Colored lines and 95% CI per DPI are displayed using conditional_effects. Scaled coordinates at 0.0 are the dorsolateral cortex and 1 close to the ventricle (see Figure S8C; Table S21; source data, code, and plot). (D) (left) Representative image (20× magnification) of PDGFR*β* ^tdTomato^/KLF4^+^/CD31^+^ immunolabeling in brain sections at 7 and 30 DPI. (Middle) KLF4^+^ cells detected in CellProfiler with corresponding overlay over PDGFR*β* ^+^ cells (right) (see the entire image set). (E) Fitted model for Colocalized|trials(PDGFR*β*)*~*DPI × Region, family = binomial (7–30 DPI). Posterior estimates are displayed with conditional_effects (mean +95% CI) (see Figure S8D–F; Table S23; source data, code, and plot). High‐resolution figure: https://osf.io/jxb4z/files/42tur.

In the striatum, we observed that the connected components merged more rapidly at 7 and 14 DPI (~120 filtration value) [Wasserstein 0‐D: 227, 95% CI = 184–316]. Together with the early formation of loops at these time points (Figure 5C, middle‐green/magenta), we argue that topological changes in the striatum are evident during the 1st and 2nd week after ischemic stroke. Note, however, that the median distances for the pairs 0–3 DPI [Wasserstein 0‐D: 385, 95% CI = 169–549] and 3–7 DPI [Wasserstein 0‐D: 349, 95% CI = 222–785] are larger with considerable uncertainty. We interpret these changes as small‐scale interactions, aggregations of few cells that create some subtle non‐homogeneities in the tissue. Otherwise, although the merging of connected components at 30 DPI (yellow) is similar to 0 (red) and 3 DPI (blue) (Figure 5C, middle), the late appearance of loops at 30 DPI (1‐dimensional homology, yellow) [Wasserstein 1‐D: 166, 95% CI = 143–183] indicates fewer, but more extensive, interactions between cells. The striatum becomes a uniform, large‐scale inter‐connected monolayer of reactive PDGFR*β* ^+^ cells between the 1st and 2nd week after ischemic stroke. Conversely, the cortex shows a distinct, decreasing gradient of merging components (0‐dimensional homology) over the course of injury progression after stroke (Figure 5C, right). Transitions are larger between 3 and 7 DPI [Wasserstein 0‐D: 603, 95% CI = 508–669], while the smallest are between 14 and 30 DPI [Wasserstein 0‐D: 157, 95% CI = 119–225]. This suggests that, in the cortex, a mature fibrotic scar formed by reactive PDGFR*β* ^+^ cells is steadily established from the 2nd week after injury and undergoes little change thereafter. Compared to the striatum, the maximum merging of connected components (14 and 30 DPI) occurs at ~75 filtration, indicating a denser arrangement of cells. Together with the analysis of 1‐dimensional homology, our results show that the mature scar formed by reactive PDGFR*β* ^+^ cells (14–30 DPI) in the cortex consists of small cell clusters that rapidly form a monolayer at small scales. In Figure 5E, we show examples of different filtration values for representative time points per region and bottleneck distances are shown in Figure S7F. Overall, our analysis reveals that the topological organization of reactive PDGFR*β* ^+^ cells evolves differently in the cortex compared to striatum and peri‐lesion. Reactive PDGFR*β* ^+^ cells in the injured cortex progressively form small‐scale cell aggregations that generate a large‐scale homogeneous monolayer in the 2nd week after stroke. At this stage, the fibrotic scar reaches structural maturity beyond which reactive PDGFR*β* ^+^ cells do not further reorganize.

### 3.7 KLF4 Expression Is Induced at the Injured Brain Upon Ischemic Stroke

Previous investigations in different pathological contexts have shown that KLF4 is a critical regulator of the response of peripheral PDGFR*β* ^+^ cells to hypoxic/ischemic insults (Chandran et al. 2021; Murgai et al. 2017). Therefore, we investigated the expression of KLF4 in our experimental stroke model with emphasis on its regulation in PDGFR*β* ^+^ cells using immunofluorescence. First, we report that, in our experimental conditions, KLF4 is barely expressed in brain perivascular PDGFR*β* ^+^ cells but is abundantly expressed in CD31^+^ brain endothelial cells (see immunolabeling protocol and raw images). Then, we used PPA and co‐localization analysis to evaluate the changes in KLF4 immunoreactivity and its expression in brain PDGFR*β* ^+^ cells over the course of injury progression. Our PPA analysis in the ipsilateral hemisphere (see QN; Supplementary Method 14; Figure 6A‐upper row) shows that the overall spatial intensity of KLF4 decreases (*β*_1_ = −0.62) the closer to the intact regions. Nevertheless, modeling of the mean spatial intensity shows that KLF4 immunoreactivity decreases early after injury (3 DPI), and it is progressively restored until the chronic phase (30 DPI) (Figures 6B and S8A,B; Table S20). This suggests that there is no net increase in overall KLF4 expression in the injured brain, but rather expression recovery. Additionally, we modeled the staining intensity per KLF4^+^ cell nuclei using a multilevel model (see QN; Figures 6A‐bottom row and  S8A,B). Our results indicate that the staining intensity of KLF4 increases from 3 to 14 DPI (Figure S8C; Table S21). We observed that this intensity pattern is also influenced by the position of KLF4^+^ cell nuclei in the x‐coordinates (dorsolateral cortex to ventricles) (Figure 6C; Table S22), with more intense nuclei near the dorsolateral cortex at 14 [*β*_1_ = −0.54, 95% CI = −0.67 to −0.41] and 30 DPI [*β*_1_ = −0.31, 95% CI = −0.47 to −0.15]. Taken together, our data indicate that the number of KLF4^+^ cells does not exceed baseline levels after injury but exhibits a redistribution and marker intensity near the injured region. Next, we evaluated KLF4 expression specifically in PDGFR*β* ^+^ cells in defined ROIs (contralateral hemisphere, healthy peri‐lesional region, and injured cortex) at the time points associated with its upregulation (7, 14, and 30 DPI) (QN; Supplementary Method 15; Figure 6D). Quantification of PDGFR*β*/KLF4 co‐localization is challenging given the anatomical closeness/overlapping of PDGFR*β* ^+^ cells with the CD31^+^ brain endothelial cells that abundantly express KLF4. For this reason, we used the contralateral hemisphere as a reference, accounting for false‐positive co‐localization rates. We estimated that this error oscillates between 5% and 10% (see QN and Figure S8D,E). Our co‐localization analysis shows that a maximum of ~20% of PDGFR*β* ^+^ cells display detectable KLF4 expression in the injured cortex at 30 DPI [*β*_1_ = 0.34, 95% CI = 0.15–0.53] (Figure 6E; Table S23). Considering the estimated co‐localization error rate and the false positives that may arise from increased cell density, we conclude that KLF4 expression is barely induced in reactive PDGFR*β* ^+^ cells and is very subtle compared to that of CD31^+^ brain endothelial cells. Figure S8F shows the estimated probability of PDGFR*β* ^+^/KLF4^+^ co‐localization relative to the total number of PDGFR*β* ^+^ cells which is also close to 10% at its maximum when resting the false‐positive rate (contralateral hemisphere).

### 3.8 KLF4 Induction in Reactive PDGFR*β* ^+^ Cells Is Minimal Upon Ischemic Stroke

To further assess whether the subtle induced expression of KLF4 in reactive PDGFR*β* ^+^ cells is biologically significant, we downregulated its expression specifically in PDGFR*β* ^+^ cells after ischemic stroke onset in PDGFR*β* ^KLF4‐KO^ mice. We chose this strategy to target KLF4 induced expression at its peak, 14  DPI (see Supplementary Methods 16). First, we obtained the mean spatial intensity using PPA, analogous to our analysis of PDGFR*β* ^tdTomato^ cells (Figure 7A). The comparison between genotypes [*β*_1_ = 258, 95% CI = −805 to 1392] (Figure 7B,C; Table S24) revealed no discernible differences in KLF4 expression in the ipsilateral hemisphere. This indicates that attenuation to KLF4 induced expression after stroke specifically in PDGFR*β* ^+^ cells do not largely contribute to its overall expression in the ipsilateral hemisphere. This finding is consistent with our previous results indicating that KLF4 expression is minimal in reactive PDGFR*β* ^+^ cells and, therefore, not appreciable at the larger scale of the ipsilateral hemisphere. Given the close contacts between PDGFR*β* ^+^ cells and CD31^+^ vasculature, co‐localization studies using anti‐PDGFR*β* antibodies are non‐conclusive. Our results clearly indicate that KLF4 is minimally regulated in PDGFR*β* ^+^ cells after ischemic stroke.

**FIGURE 7:**
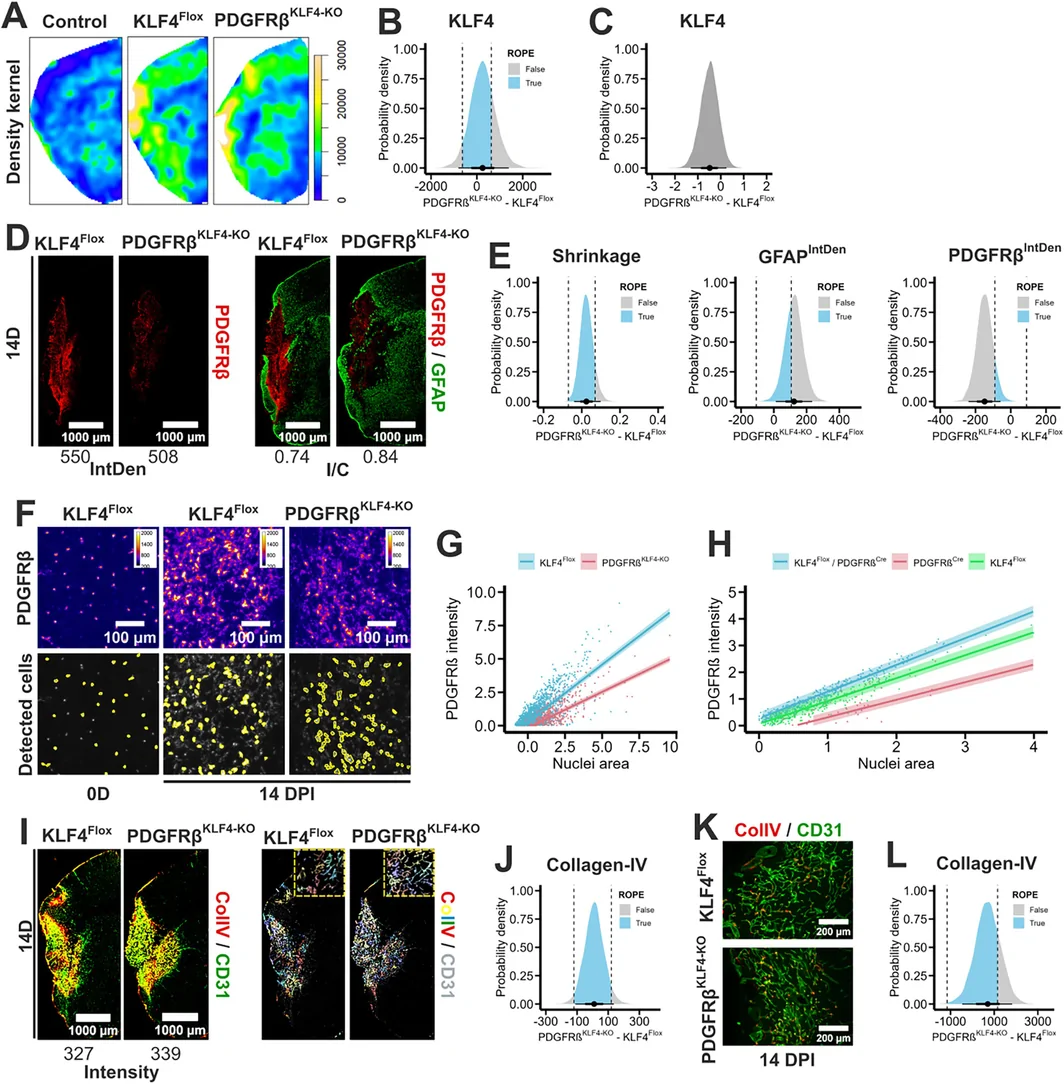
Regulation of KLF4 expression in PDGFR*β* ^KLF4‐KO^ mice. (A) Representative density kernels for KLF4^+^ cells (stitched, 10× magnification) in the ipsilateral hemisphere of control and PDGFR*β* ^KLF4‐KO^ mice. The plots are represented using the topo.colors (256) scale (see QN and point patterns). (B) Contrast between the spatial intensity of KLF4^+^ cells in the ipsilateral hemisphere of PDGFR*β* ^KLF4‐KO^ mice and corresponding controls for the linear model Intensity~Genotype, sigma~Genotype, family = student. Posterior draws (spread_draws) are shown with stat_halfeye() and ROPE (see Table S24; source data, code, and plot). (C) Probability density for sigma (unexplained variance) for the model in (B) (see plot). (D) Representative PDGFR*β* (left) and PDGFR*β* ^+^/GFAP^+^ immunolabeling in brain sections (right) (stitched, 10× magnification) at 14 DPI. Reference values for PDGFR*β* IntDen (left) and ratio ipsilateral/contralateral (I/C) (right) are shown for both genotypes (see the entire image set). (E) Contrast in hemispheric shrinkage (ipsilateral/contralateral) (left), GFAP IntDen (center) and PDGFR*β* IntDen (right) in the ipsilateral hemisphere of PDGFR*β* ^KLF4‐KO^ mice and corresponding controls for the linear models Shrinkage~Genotype, family = student, and IntDen~Genotype, family = student, respectively. Posterior draws (spread_draws) are shown with stat_halfeye() and ROPE (see Tables S25–S27; source data, code, and plots). (F) (upper row) Representative images (10× magnification, ‘fire’ LUT) of ROIs in the ipsilateral cortex labeled for PDGFR*β* mRNA (FISH) at 0 and 14 DPI. Yellow represents higher PDGFR*β* expression (bottom row) corresponding cell detections in CellProfiler showing individual PDGFR*β* ^+^ nuclei (see entire image set). (G) Fitted model for Intensity~Area × Genotype + (1|MouseID), family = student. Colored lines and 95% CI per DPI are displayed using conditional_effects (see Table S28; source data, code, and plot). (H) Fitted model for Intensity~Area × Genotype + (1|MouseID), family = student. Colored lines and 95% CI per DPI are displayed using conditional_effects (see Table S32; source data, code, and plot). (I) Representative ColIV^+^/CD31^+^ (left) immunolabeling in brain sections (stitched, 10× magnification) at 14 DPI. Reference values are shown for ColIV total intensity. (Right) ColIV signal (colored) in CD31^+^ endothelial cells, as analyzed in CellProfiler (see the entire image set). (J) Contrast between ColIV intensity in CD31^+^ endothelial cells in the ipsilateral hemisphere of PDGFR*β* ^KLF4‐KO^ mice and corresponding controls for the linear model Intensity~Genotype family = student. Posterior draws (spread_draws) are shown with stat_halfeye() and ROPE (see Table S34; source data, code, and plot). (K) Representative images (10× magnification) of ColIV^+^/CD31^+^ immunolabeling in brain sections at 14 DPI from PDGFR*β* ^KLF4‐KO^ mice and corresponding controls (see the entire image set). (L) Contrast between ColIV^+^ area of CD31^+^ vasculature in cortical ROIs of the ipsilateral hemisphere of PDGFR*β* ^KLF4‐KO^ mice and corresponding controls for the linear model Area*~*Genotype family = student. Posterior draws (spread_draws) are shown with stat_halfeye() and ROPE (see Table S36; source data, code, and plot). High‐resolution figure: https://osf.io/jxb4z/files/xv4bt.

### 3.9 Attenuation of KLF4 Induction in PDGFR*β* ^+^ Cells Does Not Affect Tissue Scar

Next, we analyzed structural changes, specifically hemispheric shrinkage, as well as glial and fibrotic scar formation in PDGFR*β* ^KLF4‐KO^ mice and corresponding controls (Supplementary Method 17; Figure 7D–G), as previously described (see Supplementary Method 1–3). Our results showed no differences in brain shrinkage between genotypes [*β*_1_ = 0.02. 95% CI = −0.04 to 0.09] (Figure 7E, left; Table S25) but a subtle increase in GFAP IntDen in PDGFR*β* ^KLF4‐KO^ mice [*β*_1_ = 122. 95% CI = 12–233] (Figure 7E, center; Table S26). Furthermore, we observed a decrease in PDGFR*β* expression in these mice [*β*_1_ = −148, 95% CI = −239 to −57] (Figure 7E, right; Table S27). We further analyzed PDGFR*β* IntDen conditioning on shrinkage ratio and ipsilateral area (Figure S9A; Table S28). These results suggest that PDGFR*β* ^KLF4‐KO^ mice may experience reduced PDGFR*β* expression with increasing hemispheric atrophy [*β*_1_ = −145. 95% CI = −239 to 52]. We further explored the regulation of PDGFR*β* in PDGFR*β* ^KLF4‐KO^ mice at the mRNA level using fluorescence in situ hybridization (FISH) (See Supplementary Method 18). Employing unbiased, automated detection methods, we identified individual PDGFR*β* ^+^ cell nuclei and analyzed their number and fluorescence intensity (see QN and Figure 7F). Our analysis indicated no appreciable changes in the number of PDGFR*β* ^+^ nuclei in PDGFR*β* ^KLF4‐KO^ mice compared to controls [*β*_1_ = −0.10, 95% CI = −0.38 to 0.16] (Figure S9D,E; Table S30). However, we observed that the labeling intensity of PDGFR*β* ^+^ nuclei was reduced in PDGFR*β* ^KLF4‐KO^ mice [*β*_Area x KO_ = −0.32, 95% CI = −0.34 to −0.29] (Figure 7G; Figure S8E; Table S31). Furthermore, we found no evidence of altered topological arrangements at the examined scales (ROIs, 10× magnification) resulting from KLF4 downregulation in brain PDGFR*β* ^+^ cells (see QN; and Figure S9F). To verify whether the changes in PDGFR*β* mRNA intensity were not an artifact of the transgene constructions, we evaluated the same marker in healthy KLF4^Flox^ and PDGFR*β* ^CreERT2^ mice (see QN; and Supplementary Method 19; Figure S9G). We included PDGFR*β* ^tdTomato^ mice as controls. Surprisingly, we found that PDGFR*β* ^tdTomato^ mice exhibited a higher number of nuclei compared to PDGFR*β* ^CreERT2^ [*β*_Cre_ = −51, 95% CI = −78 to −24] but not when compared to KLF4^Flox^ [*β*_Flox_ = −2.5, 95% CI = −33 to 27] (Figure S9H; See Table S32). Conversely, an analogous analysis of PDGFR*β* labeling intensity revealed that both PDGFR*β* ^CreERT2^ [*β*_Area x Cre_ = −0.34, 95% CI = −0.37 to −0.31] and KLF4^Flox^ [*β*_Area×Flox_ = −0.14, 95% CI = −0.16 to −0.12] mice exhibited lower PDGFR*β* mRNA intensity compared to control animals (Figure 7H; Supplementary Table 33). This implies that PDGFR*β* expression is altered in PDGFR*β* ^KLF4‐KO^ mice, which warrants further investigation. The potential role of KLF4 regulatory functions in affecting spatial reorganization (3–7 DPI) during the acute/subacute phases, we conducted an additional set of experiments initiating its downregulation prior to stroke onset (Figure S9B,C; Table S29A,B). Under this paradigm, we found no evidence of alterations in PDGFR*β* [*β*_1_ = 18.3, 95% CI = −41 to 74.4] or GFAP expression [*β*_1_ = −54, 95% CI = −162.4 to 58.1] in PDGFR*β* ^KLF4‐KO^ mice compared to control animals. This further confirms that KLF4 does not play a major role in regulating the response of PDGFR*β* ^+^ cells to brain injury after ischemic stroke.

### 3.10 KLF4 Attenuation in PDGFR*β* ^+^ Cells Does Not Influence Vascular Responses

Given the close interaction of a subpopulation of reactive PDGFR*β* ^+^ cells with the vasculature, we extended our exploration to CD31^+^ brain endothelial cells. We analyzed vascular reactivity by measuring the intensity of collagen‐IV (ColIV) in CD31^+^ cells in the ipsilateral hemisphere (QN, Figure 7I; Supplementary Method 20). We observed that attenuation of KLF4 expression in PDGFR*β* ^+^ cells does not affect vascular ColIV expression in the ipsilateral hemisphere [*β*_1_ = 9.9, 95% CI = −111 to 136] (Figure 7J; Table S34). Additionally, we performed an alternative analysis of CD31 and ColIV in defined ROIs within the injured cortex (QN, Figures 7K and S9A; Supplementary Method 20). We found that the area occupied by CD31^+^ vasculature was similar between PDGFR*β* ^KLF4‐KO^ and controls [*β*_1_ = 272, 95% CI = −472 to 999] (Figure S9B,C; Table S35). However, PDGFR*β* ^KLF4‐KO^ mice exhibited greater variability in CD31 reactivity [*𝜎*1 = 1.05, 95% CI = 0.23–1.86] (Figure S9B, right; Table S35), outlining a potentially more heterogeneous response to ischemic stroke. Similarly, we did not observe differences in the area occupied by ColIV^+^ structures associated with CD31^+^ endothelial cells [*β*_1_ = 699, 95% CI = −466 to 1825] (Figures 7L and S10A–C; Table 35). This is consistent with the analysis of total collagen in the injured cortex and striatum using Picosirius staining (QN, Figure S9D–F). Although attenuation of KLF4 expression in PDGFR*β* ^+^ cells seems to affect the mean of ColIV^+^ in the injured cortex [*β*_Ctx‐KO_ = 511, 95% CI = 14–1573], the considerable uncertainty [*𝜎*_1_ = 3974, 95% CI = 3062–5150] hampers any definitive conclusions (Figure S10F; Tables S36 and S37). Our results suggest that although attenuation of KLF4 induction in PDGFR*β* ^+^ cells may augment the variability of injury responses, it does not noticeably affect overall vascular reactivity.

## 4 Discussion

In this study, we aimed to analyze the reactivity of brain PDGFR*β* ^+^ cells and contribution to scar formation upon stroke with emphasis on temporal morphological changes and topological rearrangements. Furthermore, we aimed to elucidate whether KLF4 regulates the reactivity of brain PDGFR*β* ^+^ cells upon stroke, as reported in injured peripheral organs. This was achieved using PDGFR*β* ^tdTomato^ reporter mice and PDGFR*β* ^KLF4‐KO^ transgenic mice subjected to experimental ischemic stroke, implicating the generation of a defined cortico‐striatal injury associated with spatiotemporally well‐organized progressive scar formation. First, we analyzed the reactivity patterns of PDGFR*β* ^+^ cells in the ipsilateral hemisphere using IntDen and PPA. Our findings indicate that the reactivity of PDGFR*β* ^+^ cells is integral to glial scar formation and is closely linked to brain atrophy. This observation is consistent with previous studies, which identified, using qualitative approaches, compartmentalization of PDGFR*β* ^+^ cells within a GFAP^+^ cell convex hull (Reeves et al. 2019). Herein, we further explored this compartmentalization by providing a quantitative model based on PPA to characterize the distribution of PDGFR*β* ^+^ cells within the injured tissue in relation to scar‐forming GFAP^+^ astrocytes, as previously validated (Manrique‐Castano et al. 2024). Our findings confirm that the boundary of the GFAP^+^ cell convex hull constitutes a “no‐entry” point for reactive astrocytes to limit the extension of the outer astroglial layer (Shibahara et al. 2020). The origin of reactive PDGFR*β* ^+^ cells remains a matter of debate and evidence is outlining the potential contribution of resident perivascular cells, including pericytes and fibroblasts (Dias et al. 2021; Shibahara et al. 2020), as well as infiltrating PDGFR*β* ^+^ cells (Jung et al. 2011; Kokovay et al. 2006). Further research is warranted to elucidate the specific functions of reactive PDGFR*β* ^+^ cells and contribution to overall scar organization after stroke.

Several transcriptomic studies have outlined the presence of a PDGFR*β* ^+^ astrocyte subpopulation (Sadick et al. 2022), which is proposed to contribute to glial scar compartmentalization (Dias et al. 2021; Kyyriäinen et al. 2017). Evidence is indicating that germline depletion of PDGFR*β* in neurons and astrocytes increases vascular leakage and increases infarction after ischemic insults (Shen et al. 2012). This suggests that the subpopulation of PDGFR*β* ^+^ astrocytes may contribute to the maintenance of the homeostasis of neurovascular interactions. In 2021, the manufacturer indicated that PDGFR*β* antibody (Abcam, ab32570, RRID:AB_777165) could react with PDGFR*α* that is expressed in a subpopulation of perivascular astrocytes and fibroblasts (Su et al. 2008). The potential of non‐specific labeling of different cell populations in studies utilizing the anti‐PDGFR*β* antibody (ab32570) underscores the need for careful interpretation of results. Beyond the technical constraints involving antibody specificity, we acknowledge that the discrepancies in the reported reactivity of PDGFR*β* ^+^ cells could arise from the inherent biological variabilities and the divergence of the different used experimental stroke models. For instance, our data show that, in contrast to mild striatal‐specific injury, severe cortico‐striatal injury mediates a robust spatial segregation and accumulation of PDGFR*β* ^high^ cells in the injury core. Thus, variations in ischemic insult, extent of reperfusion injury, and ensuing tissue loss across studies will inevitably result in disparate observations regarding PDGFR*β* ^+^ cell detachment, proliferation, and fibrotic transition.

Next, we analyzed the proportion of perivascular and parenchymal reactive PDGFR*β* ^+^ cells after ischemic stroke and explored their proliferative potential using Ki67. Our results indicate that upon stroke, parenchymal PDGFR*β* ^+^ cells emerge mainly in the injured cortex. The detachment of PDGFR*β* ^+^ pericytes from the vasculature has been reported in various brain injuries (Dore‐Duffy et al. 2000). This process is thought to be controlled by the regulator of G‐protein signaling (RGS)5 induced by hypoxia (Enström et al. 2022). Indeed, RGS5^KO^ mice exhibit a reduced density of parenchymal PDGFR*β* ^+^ cells during the 1st week of stroke (Roth et al. 2019), which are associated with a “less reactive” phenotype based on cell projections. It has been reported that reactive PDGFR*β* ^+^ cells detach from the vasculature and reprogram to acquire fibroblast‐like characteristics (Dias et al. 2021; Fernandez‐Klett et al. 2013; Shibahara et al. 2020). Other reports have indicated that PDGFR*α* ^+^ perivascular fibroblasts, which express as well PDGFR*β*, contribute to the fibrotic reaction upon brain injuries, including stroke (Dorrier et al. 2022). In our PDGFR*β* ^tdTomato^ reporter mice, we did not observe tdTomato expression in PDGFR*α* ^+^ cells detected using the Abcam antibody (ab203491, RRID:AB 2892065) in the healthy brain. Detachment of reactive PDGFR*β* ^+^ cells is often reported to be accompanied by proliferative activity (Özen et al. 2014). Using PDGFR*β* ^tdTomato^ reporter mice, we observed an increased proportion of reactive PDGFR*β* ^+^ parenchymal cells at the lesion site, which cannot be exclusively linked to vascular detachment and subsequent parenchymal transition. In PDGFR*β* ^tdTomato^ reporter mice, in which the potency of tdTomato expression depends upon basal PDGFR*β* expression that drives the expression of Cre‐recombinase, we reported an increased intensity at time points during which vascular detachment and parenchymal transition were reported to peak (Fernandez‐Klett et al. 2013). Following a prolonged period after initial Cre‐recombination, we observed the emergence of a population of ramified cells that express low levels of tdTomato throughout the brain parenchyma of PDGFR*β* ^tdTomato^ reporter mice, indicative of the presence of a cell population expressing low basal levels of PDGFR*β*. It is plausible that the emergence of PDGFR*β* ^+^ parenchymal cells might be due to an increased marker expression in a population of non‐mural cells expressing low levels of PDGFR*β*, as reported (Bernard et al. 2024; Renner et al. 2003), such as PDGFR*β* ^+^ astrocytes, rather than vascular detachment per se. Moreover, the contribution of PDGFR*β* ^+^ immune cells derived from the periphery that infiltrate the injured tissue via the vasculature cannot be excluded (Jung et al. 2011; Kokovay et al. 2006). Previous reports have indicated that perivascular cells co‐expressing PDGFR*β* and CD13 leave the vasculature, proliferate and express microglial markers the 1st week after stroke (Özen et al. 2014). It is highly probable that PDGFR*β* ^+^/CD13^+^ cells are infiltrating PDGFR*β* ^+^ immune cells that are transmigrating via the injured vasculature. Although CD13 is expressed in brain pericytes under normal conditions, it has been reported to be expressed in myeloid cells that are recruited to the angiogenic vessels after ischemic stroke (Nguyen et al. 2023). The reported detachment of PDGFR*β* ^+^ cells coincides with a considerable number of cells exhibiting proliferation markers, such as BrdU or Ki67, in ischemic stroke (Fernandez‐Klett et al. 2013; Özen et al. 2014) as well as in other brain injuries (Zehendner et al. 2015). However, utilizing unbiased, automated, and reproducible workflows, we found that the proportion of reactive PDGFR*β* ^+^ cells expressing Ki67 is extremely low and cannot account for the increased overall density of cells observed at the injured tissue. This holds true for both vascular‐associated and parenchymal PDGFR*β* ^+^ cells. Unfortunately, we were unable to obtain comparable evidence using BrdU, as its incorporation requires a denaturation process that significantly damages the tdTomato signal in PDGFR*β* ^+^ cells, rendering the analysis unreliable.

Next, we characterized the morphological changes of PDGFR*β* ^+^ cells in both the injured and intact regions after stroke. Consistent with previous reports (Menezes et al. 2020; Özen et al. 2014), in PDGFR*β* ^tdTomato^ reporter mice, it is possible to identify perivascular cells co‐expressing CD13 in the healthy regions. However, we found no evidence that these cells co‐express GFAP, IBA1, or PDGFR*α* in the injured tissue. We argue that the close interaction between neighboring cells with CD31^+^ vasculature, such as PDGFR*α* ^+^ cells (Vanlandewijck et al. 2018), or the extensive superposition of reactive PDGFR*β* ^+^ cells and IBA1^+^ microglia in the injured regions (Riew et al. 2018), may lead to some misinterpretations. Furthermore, we postulate that our morphological analysis has quantitatively characterized amoeboid PDGFR*β* ^+^ cells described byZehendner et al. (2015), fibroblast‐like cells (reticulite) reported by Dias et al. (2021) and Göritz et al. (2011), and reactive RGS5^+^ cells (reticulite) identified by Özen et al. (2014). Subsequently, we analyzed the redistribution and topological arrangements of reactive PDGFR*β* ^+^ cells over the course of injury progression. We found that PDGFR*β* ^+^ cells undergo a predictable rearrangement pattern. Analyses of cell reactivity and glial scar formation have relied on qualitative assessments that lack spatial insights. Herein, we implemented PPA and TDA workflows to provide spatiotemporal insights (Manrique‐Castano et al. 2024). TDA and PPA have broad applications in biological (Ben‐Said 2021; Bhaskar et al. 2023; McGuirl et al. 2020) and non‐biological sciences (Baddeley et al. 2021; Bilotti et al. 2024). While standard density metrics indicate a general increase in cell numbers, our TDA specifically uncovers that reactive PDGFR*β* ^+^ cells in the injured cortex transition into a large‐scale homogeneous monolayer by the 2nd week of stroke, a distinct micro‐architectural maturation that is absent in the injured striatum. Our approach using these new tools offers a viable alternative to overcoming raw cell counts or density estimations that lack comprehensive spatiotemporal information.

Herein, we extended our previous analyses of NeuN^+^, GFAP^+^, and IBA1^+^ cells (Manrique‐Castano et al. 2024) to include PDGFR*β* ^+^ cells, while providing expanded reproducible workflows. These incorporate the analysis of Haralick features (Löfstedt et al. 2019), the implementation of bootstrapping (Chernik 2007) to analyze Wasserstein and bottleneck distances (Hajij et al. 2018), and the generation of Vietoris‐Rips complexes at different filtration values (Chambers et al. 2010). A limitation in our study is the identification of PDGFR*β* ^+^ cells using CellProfiler and QuPath, particularly in regions with high cell density. This inaccuracy may be influenced by multiple factors, including labeling patterns, cell morphology, and cell overlaps. These constraints are especially pronounced when dealing with ramified and variable‐intensity cells, as current algorithms are primarily optimized for detecting round‐shaped cells (Al‐Kofahi et al. 2018; Stringer et al. 2021). Consequently, our approach estimates local cell density but is limited in its ability to precisely quantify the absolute number of cells within ROIs or time windows. As these computational hurdles prevent the unequivocal decoupling of absolute cell counts from the overlapping fluorescence signals in highly compacted tissues, intensity‐based metrics (such as IntDen) adequately capture a representative composite pattern. Therefore, variations in IntDen at the lesion site could be interpreted as a combined effect of localized cellular accumulation and potent Cre‐recombination driven by high PDGFR*β* basal expression. We acknowledge that the segmentation of ramified cells remains a significant challenge that requires further advancement, particularly through machine learning‐based methods that leverage morphological features for cell segmentation. Addressing this limitation represents an important avenue for future research.

We next aimed to investigate the mechanisms underlying the transition of quiescent PDGFR*β* ^+^ cells towards a reactive state upon injury. In peripheral organs, KLF4 acts as a master regulator of the activation, reprogramming, and fibrotic trans‐differentiation of PDGFR*β* ^+^ cells in response to hypoxic or ischemic insults (Chandran et al. 2021; Shankman et al. 2015; Sheikh et al. 2015). It has been assumed that KLF4 regulatory functions are conserved across PDGFR*β* ^+^ cells in various organs. However, our findings strongly challenge this paradigm by demonstrating that KLF4 is not involved in regulating the reactivity of brain PDGFR*β* ^+^ cells upon ischemic stroke. This could be linked to a fundamental evolutionary and developmental divergence related to origins of PDGFR*β* ^+^ cells in various organs (Korn et al. 2002). Thus, brain PDGFR*β* ^+^perivascular cells appear to be governed by regulatory mechanisms that are distinct from their peripheral counterparts. As KLF4 is only induced in PDGFR*β* ^+^ cells after stroke, we have adopted 2 different strategies to fully elucidate its regulatory functions. This was achieved by mediating KLF4 conditional downregulation in PDGFR*β* ^+^ cells either before or after stroke onset. The conditional attenuation of KLF4 in PDGFR*β* ^+^ cells, whether initiated prior to ischemic stroke onset, which aims to assess the effects of its early regulatory functions between 3 and 7 DPI, or after onset, which aims to explore the effects of attenuating its induced expression at 14 DPI, resulted in no meaningful changes in injury progression, cell morphology, or scar organization. Likewise, vascular ColIV deposition remained unchanged in PDGFR*β* ^KLF4‐KO^ mice. Nonetheless, we acknowledge that while our models suggest that manipulation of KLF4 in reactive PDGFR*β* ^+^ cells does not seem to affect the macroscopic structural reorganization, we cannot completely exclude possible transient region‐specific effects. The macroscopic changes do not fully mirror the pathophysiology; therefore functional assessments, such as vascular permeability or detailed inflammatory profiling, are warranted in future investigation.

Our findings provide different perspectives for the reported upregulation of KLF4 in pericytes and S100^+^ astrocytes after stroke (Nakagomi et al. 2015; C. Wang and Li 2023). Using a validated antibody (ab214666) on both PFA‐ and glyoxal‐fixed tissues, we observed stable, abundant nuclear KLF4 expression largely restricted to CD31^+^ brain endothelial cells, without compelling evidence of widespread upregulation in reactive astrocytes. We emphasize that assessing co‐localization between a nuclear marker (KLF4) and ramified structures, such as GFAP, in compacted injured tissue is exceptionally prone to artifacts, underscoring the need for open‐source raw data to validate such claims. Furthermore, as mentioned before, beyond the technical constraints, discrepancies with some of the current literature could as well be likely driven by the biological variabilities and the different experimental stroke models. In contrast to the findings that have outlined KLF4's role in regulating the reactivity of PDGFR*β* ^+^ cells in peripheral organs, which was supposed to be conserved in all PDGFR*β* ^+^ cells, our observations indicate that KLF4 does not fulfill the same functions in brain PDGFR*β* ^+^ cells. As indicated, this difference could be due to the distinct origin of brain PDGFR*β* ^+^ cells that are mainly derived from the neuroectoderm, whereas peripheral PDGFR*β* ^+^ cells originate from the mesoderm (Korn et al. 2002). A limitation of our study is the inability to perform an unbiased, single‐cell analysis of KLF4 due to the close interaction between CD31^+^ and PDGFR*β* ^+^ cells, and the high cell density in injured tissue. While our data demonstrate that KLF4 is not implicated in regulating the reactivity of brain PDGFR*β* ^+^ cells, a context‐dependent role restricted to the hyper‐acute phase cannot be fully excluded. Nonetheless, our pre‐onset depletion paradigm suggests that even during the early phases of acute tissue reorganization, KLF4 remains dispensable for subsequent scar organization. Our findings underscore the need to reconsider the approaches that investigate KLF4 and PDGFR*β* using immunohistochemistry, especially following CNS injuries.

Finally, one scope of our study is the use of model‐based Bayesian inference with brms and MCMC, which allows us to obtain precise parameters/coefficients with their respective uncertainties. We are convinced that our scientific inference based on effect sizes and full posterior distributions (Kruschke and Liddell 2018), which draws on a coherent family of distributions and statistical (linear and nonlinear) models, provides a more informative approach than threshold‐based analysis based on the uninformative notion of significance. We make our statistical models (as. R objects) and raw data available as open source to promote their reproducibility and validation in further research. We are convinced that our models can be used as priors or quantitative information for research in the field of brain injuries.

## 5 Conclusion

In this study, we provide fully open‐source, model‐based quantitative workflows to comprehensively decode the spatiotemporal distribution of brain PDGFR*β* ^+^ cells following brain injuries in relation to scar organization. Moving beyond the qualitative descriptions of macroscopic scar compartmentalization, our predictive models reveal a heterogeneous reactivity of PDGFR*β* ^+^ cells in the injured brain. Notably, our findings indicate that the presence of reactive PDGFR*β* ^+^ parenchymal cells cannot be exclusively linked to localized cell proliferation or vascular detachment, thus providing a new framework to further investigate the diversity of PDGFR*β* ^+^ cell populations. Furthermore, we demonstrate that, in contrast to PDGFR*β* ^+^ cells in injured peripheral organs, KLF4 regulatory functions are not required to mediate the reactivity of brain PDGFR*β* ^+^ cells and consequently spatial reorganization of scar tissue after ischemic stroke. This suggests that brain PDGFR*β* ^+^ perivascular cells are governed by distinct regulatory mechanisms compared to peripheral cells, likely reflecting their respective diverse and distinct developmental origins. Finally, to foster reproducibility and allow the community to validate, challenge, or build upon our approach, we make all raw images, point clouds, analysis codes, and Bayesian models freely available.

## Author Contributions

Ayman ElAli and Daniel Manrique‐Castano designed the study. Daniel Manrique‐Castano performed the experiments and prepared the figures. Ayman ElAli and Daniel Manrique‐Castano validated the analysis and interpreted the results. Ayman ElAli and Daniel Manrique‐Castano drafted and edited the manuscript.

## Funding

This work is supported by grants from the Heart and Stroke Foundation of Canada (HSFC) (G‐23‐0035047) and the Canadian Institutes of Health Research (CIHR) (169062; 186148) (all to Ayman ElAli). Daniel Manrique‐Castano is supported by Fonds de recherche du Québec (FRQS)‐Santé (318466). Ayman ElAli holds a Tier 2 Canada Research Chair in molecular and cellular neurovascular interactions.

## Ethics Statement

Animal studies were performed according to the Canadian Council on Animal Care guidelines, as implemented by the institutional animal ethics committee (Comité de Protection des Animaux de l'Université Laval‐3 (CPAUL‐3); Protocol # 20‐470) and were reported according to ARRIVE 2.0 guidelines.

## Conflicts of Interest

The authors declare no scientific or financial conflicts of interest.

## Supporting information


**Figure S1:** Reactivity of PDGFR*β* ^+^ and GFAP^+^ cells after ischemic stroke. (A) Representative brain section (stitched, 5× magnification) at 3 and 14 DPI showing the astroglial scar formed by GFAP^+^ astrocytes. Integrated density (IntDen) values are shown at the button as a reference. (B) Fitted splines model for GFAP_IntDen_~s(DPI). The conditional_effects are shown with the mean (blue) and 95% CI (gray‐shadowed region) and observations as diamonds. (C) Fitted splines for GFAP_IntDen_~t2(DPI, Area). The conditional_effects are displayed with a Viridis scale (IntDen). (D) Model derivatives calculated with modelbased for the non‐linear models fitted for brain shrinkage (left), PDGFR*β* (middle), and GFAP (right). The curves show the effect of each DPI (positive or negative) in the response variable. (E) Display the sigma (𝜎) coefficients and their respective uncertainty for the fitted models for brain shrinkage (left), PDGFR*β* (middle), and GFAP (right). The results are shown in the log scale. (F) PDGFR*β* ^low^ density kernels (left) at 7, 14, and 30 DPI. The density kernels are represented using the topo.colors(256) scale. The relative distribution diagrams (right) depict the probability of the allocation of PDGFR*β* ^low^ cells (y‐axis) relative to GFAP spatial intensity (*x*‐axis). The pooled mean intensity (cyan‐dashed line) is shown with their corresponding lower (black‐solid line) and upper (green‐dotted line) limits of the two‐sigma confidence intervals. Note the dissimilar *xy* axes limited by the mapping of the rhohat function for each DPI. Diagrams for PDGFR*β* ^high^ are displayed in Figure 2H,I. (G) Representative GFAP^+^/PDGFR*β* ^tdTomato^ immunolabeling in brain sections (stitched, 5× magnification) showing the GFAP^+^ cell convex hull (yellow contour). Reactive PDGFR*β* ^+^ cells allocate primarily outside these boundaries. (H) Fitted linear model for GFAP/contralateral area~DPI. We display stat_lineribbon() with the mean hemispheric area (black) and different credible intervals: 0.05, 0.8, 0.95 (blue brewer scale). The observations are displayed as black diamonds. High‐resolution figure: https://osf.io/jxb4z/files/ztfb6.
**Figure S2:** Distribution of PDGFR*β* ^+^ cells upon striatal‐specific injury. (A) Sample crops of PDGFR*β* ^low^ (non‐reactive) and PDGFR*β* ^high^ (reactive) cells in the ischemic hemisphere of animals with ischemic stroke‐mediated striatal‐specific injury. (B) Fitted binomial model for PDGFR*β* ^high^|PDGFR*β* ^total^~DPI in mice with striatal‐specific injury. The conditional_effects are displayed with the mean (blue) and 95% CI (gray‐shadowed region). (C) Representative PDGFR*β* ^tdTomato^/GFAP^+^ immunolabeling in brain sections (left) (stitched, 5× magnification) with corresponding PDGFR*β* ^+^ cell density kernels (right) at 14, and 30 DPI in animals with striatal‐specific injury. The density kernels are represented using the topo.colors(256) scale. (D) Relative distribution diagrams (*rhohat*) depicting the allocation probability of PDGFR*β* ^high^ cells (y‐axis) relative to GFAP spatial intensity (x‐axis) in animals with striatal‐specific injury. The pooled mean intensity (cyan‐dashed line) is shown with their corresponding lower (*black‐*solid line) and upper (green‐dotted line) limits of the two‐sigma confidence intervals. Note the dissimilar *xy* axes limited by the mapping of the rhohat function for each DPI. (E) Representative GFAP^+^/PDGFR*β* ^tdTomato^ immunolabeling in brain sections (upper rows) (10× magnification) with corresponding GFAP density kernels (bottom rows) at 3, 7, 14, and 30 DPI in the healthy peri‐lesional region, the injured striatum, and the injured cortex. The density kernels are represented using the topo.colors(256) scale and PDGFR*β* ^tdTomato^ cells as black dots. (F) Fitted models for PDGFR*β*~Region. The conditional_effects() with mean cell counts and 95% CI are displayed as whiskers. At the bottom, entire posterior densities for the contrast between DPI are shown with stat_pointinterval. High‐resolution figure: https://osf.io/jxb4z/files/3q8pm.
**Figure S3:** Emergence of a subpopulation of parenchymal PDGFR*β* ^+^ cells after ischemic stroke. (A) Fitted model (Pdgfrb_Total~DPI × Region, family = hurdle_lognormal) for the total number of parenchymal PDGFR*β* ^+^ cells in the peri‐lesional region, the injured striatum, and the injured cortex. Colored dots are observations. (B) Contrast between the number of PDGFR*β* ^+^ cells in the injured striatum and the injured cortex. After vanishing at 3 DPI in the cortex, the number of PDGFR*β* ^+^ increases subsequently. (C, D) Fitted model (Pdgfrb_Total~DPI × Lesion, sigma*~*DPI × Lesion, family = student) for the comparison between striatal‐specific (striatal) and cortico‐striatal injuries at 14 and 30 DPI. (E) Fitted model (Pdgfrb_Parenchymal|trials(Pdgfrb_Total)~DPI × Lesion, family = binomial) for the proportion of parenchymal PDGFR*β* ^+^ cells in striatal‐specific (striatal) and cortico‐striatal injuries at 14 and 30 DPI. (F) Representative images (10× magnification) of PDGFR*β* ^tdTomato^/CD31^+^ immunolabeling in brain sections (upper row), and co‐localization analysis (CellProfiler) of nuclei in PDGFR*β* ^tdTomato^ cells (colored) and CD31^+^ vasculature (white) (bottom row) for sham and ischemic stroke animals (MCAo). (G) Fitted model (Pdgfrb_Parenchymal~DPI × Condition, family = student) to study the impact of Cre‐recombination on the number of parenchymal PDGFR*β* ^+^ cells in the healthy cortex. Prolonged Cre‐recombination does not affect the emergence of parenchymal PDGFR*β* ^+^ cells. (A, C–E, G). The posterior estimates are displayed with conditional_effects (mean +95% CI). High‐resolution figure: https://osf.io/jxb4z/files/ywnez.
**Figure S4:** Sorting of brain PDGFR*β* ^+^ cells after stroke. (A) FSC‐A (forward scatter area) and SSC‐A (side scatter area) from ipsilateral (ipsi) and contralateral (contra) hemispheres at 14 DPI. (B) Singlet gating, (C) tdTomato (PDGFR*β* ^+^ cells) gated from singlets and, (D) dead cells (Live/dead^+^ cells) from PDGFR*β* ^+^ cells. (A–D). The graphs are plotted using geom_hex (bins), geom_density2d (black contour), geom_gate (red gated area), geom_stats (percentage of cells in gated regions). High‐resolution figure: https://osf.io/jxb4z/files/zxku4.
**Figure S5:** Processing of brain PDGFR*β* ^+^ cells for morphological analysis. (A) Laser scan confocal microscope was used to acquire z‐stacks at 40× magnification of PDGFR*β* ^+^ cells in the cortical peri‐lesional region, the injured striatum and the injured cortex. The z‐stacks were projected in a single plane (2D cells), and images were processed using Python libraries skimage and scipy to enhance, threshold, and generate masks of single cells. Then, the morphological characteristics were measured using regionprops and regionprops_table functions from skimage.measure and data exported to R for principal component analysis (PCA). (B) Correlation plot of the morphological features used for PCA in the ggcorrplot package. The features are defined in the respective QN notebook. (C) Perivascular PDGFR*β* ^+^ (tdTomato) cells exhibit changes in the immunoreactivity to CD13 upon injury. CD13 immunoreactivity was not detected in reticuloparenchymal PDGFR*β* ^+^ cells in the healthy tissue. The meaningful induction of CD13 immunoreactivity in ischemic regions (amoeboid, right column) hinders the quantitative, unbiased analysis of PDGFR*β* ^tdTomato^/CD13^+^ immunolabeling co‐localization. (D) Logistic regression plots for the model Class~ConvexHull + BranchLenght + Intensity_Mean, family = categorical. The conditional_effects are displayed with means and 95% CI for the probability of perivascular (red), reticuloparenchymal (green), reticulate (cyan), and amoeboid (magenta) cells conditioning on defined morphological features (Convex hull, branch length, and intensity). Please note that all morphological traits have been scaled during PCA analysis. High‐resolution figure: https://osf.io/jxb4z/files/famn2.
**Figure S6:** Profiling expression of CD13, GFAP, IBA1, and PDGFR*α* in brain PDGFR*β* ^tdTomato^ cells. (A) Representative laser scan confocal images of PDGFR*β* ^tdTomato^/CD13^+^ immunolabeling in brain sections at 40× magnification in the perilesional cortex (perilesion), the injured striatum and the injured cortex. The upper panel on the left shows perivascular PDGFR*β* ^tdTomato^/CD13^+^ cells (zoomed in the yellow squared section). In contrast, reticuloparenchymal PDGFR*β* ^+^ cells do not co‐express CD13. The patterns of CD13 immunoreactive change in the striatum and cortex following injury. In particular, the ischemic cortex shows increased immunoreactivity to CD13. (B) Confocal images of PDGFR*β* ^tdTomato^/GFAP^+^ immunolabeling in brain sections at 20× magnification in the same ROIs, as previously described. GFAP^+^ cell branches appear to wrap some PDGFR*β* ^tdTomato^ nuclei in regions of strong astrogliosis. However, the origin of GFAP^+^ branches is ambiguous. (C) Confocal images of PDGFR*β* ^tdTomato^/IBA1^+^ immunolabeling in brain sections at 20× magnification in the same ROIs, as previously described. PDGFR*β* ^tdTomato^/IBA1^+^ cells were not observed in the healthy brain and IBA1 increased immunoreactivity in the injured tissue made an unbiased and reproducible analysis impracticable. (D) Widefield images (with apotome optical sectioning) of PDGFR*β* ^tdTomato^/PDGFR*α* ^+^ immunolabeling in brain sections at 20× magnification. PDGFR*β* ^+^ and PDGFR*α* ^+^ cells constitute two distinct populations in the healthy brain. Following injury, both cell types react by increasing the marker intensity and covered area. Although both markers partially co‐localize in some regions, undefined cellular morphology and non‐specific nuclear staining make co‐localization analysis challenging with the tools at our disposal. (A–D) In all cases, unbiased and automated colocalization with PDGFR*β* ^tdTomato^ cells is ambiguous given the high cell density and uncertain nuclei labeling. High‐resolution figure: https://osf.io/jxb4z/files/ebfdw.
**Figure S7:** Haralick features and topological data analysis (TDA) of reactive PDGFR*β* ^+^ cells. (A) Correlation plot of the Haraclick features used for PCA using the ggcorrplot package. See the definition of all the features in Supplementary Method 12. (B) The Bioplot of Haralick features show the similarities between the variables and its impact on each component. The analysis suggests that all the variables have a similar loading for the components. (C) Fitted model for the area covered by reactive PDGFR*β* ^+^ cells (Area*~*DPI × Region, family = student). The posterior estimates are displayed using conditional_effects with lines as mean and 95% CI. Dots are observations (see Table S18). (D) Fitted model for the probability of cell type given specific morphological features (Region*~*entropy + contrast + IDM, family = categorical). The posterior estimates are displayed using conditional_effects with lines as mean and 95% CI. (E) Representative persistent diagrams per brain region showing the birth (x‐axis) and death (y‐axis) of topological features. Zero‐ and 1‐dimensional persistent homology from point clouds were calculated using the ripser Python package. Betti curves per brain region are displayed in Figure 5. (F) Bottleneck distances between pairs of time points calculated from 0 and 1‐dimensional homology persistent diagrams. The uncertainty in the mean distances (confidence intervals) was calculated using bootstrapping with 1000 iterations (see Table S19c,d). matplotlib was used to produce heatmaps. High‐resolution figure: https://osf.io/jxb4z/files/5kumt.
**Figure S8:** Temporal regulation of KLF4 expression upon ischemic stroke. (A) (upper row) Representative smoothed intensity kernels obtained with *density* (sigma = 0.02) from spatstat at each DPI. Individual cells with KLF4 signal intensity are shown as scatter plots on the right of each density kernel. The dorsolateral brain boundary is located on the left side of the images. (bottom row) Examples of detected individual cells in CellProfiler. The complete image set is available in the OSF repository. (B) Fitted model for KLF4 intensity ~0 + DPI, sigma ~0 + DPI, family = student. The posterior probability per DPI (left) is displayed using add_predicted_draws with stat_halfeye and the sigma (heteroskedasticity) estimates with conditional_effects (mean + 95% CI). (C) Left, fitted model for single cell KLF4 signal intensity (Intensity ~0 + DPI × Scaled_CenterX + (1|MouseID), family = lognormal). The posterior probability with conditional_effects showing mean estimates with 95% CI is displayed as whiskers. Right, the posterior probability of contrasts (emmeans) between time points of interest is displayed using add_predicted_draws with stat_halfeye. (D) Fitted model for the proportion of PDGFR*β* ^+^/KLF4^+^ cells over total KLF4^+^ cells in the contralateral hemisphere (Colocalized|trials(Klf4)*~*DPI, family = binomial). This information was used to estimate a ground truth for false‐positive colocalization rates given the proximity of non‐KLF4‐expressing perivascular PDGFR*β* ^+^ cells with CD31^+^/KLF4^+^ vasculature. (E) Fitted model for the proportion of PDGFR*β* ^+^/KLF4^+^ cells over the total of KLF4^+^ cells in distinct brain regions (Colocalized|trials(Klf4)*~*Region, family = binomial). (F) Fitted model for the proportion of PDGFR*β* ^+^/KLF4^+^ cells over the total of PDGFR*β* ^+^ cells (Colocalized|trials(Pdgfrb) ~DPI × Region, family = binomial). (D–F) The posterior probability with conditional_effects showing mean estimates with 95% CI is displayed as whiskers. High‐resolution figure: https://osf.io/jxb4z/files/qgs42.
**Figure S9:** Scar tissue formation in the injured brain of PDGFR*β* ^KLF4‐KO^ mice. (A) Fitted models for the PDGFR*β* IntDen conditioning on the ipsilateral/contralateral hemispheric ratio (left) (IntDen*~*Genotype × Shrinkage, family = student), and the ipsilateral (scaled) area (IntDen*~*Genotype × Ipsilateral, family = student). PDGFR*β* ^KLF4‐KO^ mice exhibit reduced PDGFR*β* expression with increasing hemispheric atrophy (with considerable uncertainty). (B) Representative brain sections (stitched, 5× magnification) at 25 DPI from PDGFR*β* ^KLF4‐KO^ mice and corresponding controls in which KLF4 depletion was initiated prior to ischemic stroke onset. PDGFR*β* (red) and GFAP (green) immunolabeling show a mature glial scar at the chronic phase. (C) Contrast for PDGFR*β* IntDen (left) and GFAP IntDen (right) in the ipsilateral hemisphere of PDGFR*β* ^KLF4‐KO^ mice and corresponding controls for the linear models IntDen*~*Genotype, family = student. The posterior draws (*spread_draws*) are shown with *stat_halfeye()* and ROPE. (D, E) Fitted multilevel model (Counts*~*Genotype + (1|MouseID), family = negbinomial) for the number of PDGFR*β* ^+^ cells conditioning on the genotype (PDGFR*β* ^KLF4‐KO^ and KLF4^Flox^). Three images (ROIs) per mouse brain were analyzed. The posterior estimates are displayed using *add_predicted_draws* with *stat_halfeye(*), showing point estimates and 95% CI. Dots in (D) are observations. Note that E represents the variation within animals. (F) Mean Betti curves calculated from 0 (left) and 1‐dimensional homology (right). Betti curves for 0‐dimensional homology are normalized to 1 (1‐dimensional graphs are not normalized). PDGFR*β* ^+^ cells from both genotypes share similar topological features. Line plots are shown using matplotlib. (G) (upper row) Representative images (10× magnification, ‘fire’ LUT) of ROIs in the ipsilateral cortex labeled for PDGFR*β* mRNA (FISH) in the healthy brain of KLF4^Flox^/PDGFR*β* ^Cre^, PDGFR*β* ^Cre^, and KLF4^Flox^. Yellow represents higher PDGFR*β* expression. (bottom row) Corresponding cell detections in CellProfiler showing individual nuclei of PDGFR*β* ^+^ cells. (H) Fitted multilevel model (Counts*~*Genotype + (1|MouseID), family = student) for the number of PDGFR*β* ^+^ cells conditioning on the genotype (KLF4^Flox^/PDGFR*β* ^Cre^, PDGFR*β* ^Cre^, and KLF4^Flox^). Three images (ROIs) per mouse brain were analyzed. The posterior estimates are displayed using *add_predicted_draws* with *stat_halfeye(*), showing point estimates and 95% CI. Dots are observations. High‐resolution figure: https://osf.io/jxb4z/files/875wx.
**Figure S10:** Brain vascular reactivity in PDGFR*β* ^KLF4‐KO^ mice. (A) Representative images (10× magnification) of Collagen‐IV (ColIV)/CD31^+^colocalization analysis in PDGFR*β* ^KLF4‐KO^ and KLF4^Flox^ mice. Colored regions are ColIV and CD31 is CD31^+^ vasculature. (B) Fitted model for the labeled CD31 area (Sum_CD31_Area*~*Genotype, sigma*~*Genotype, family = student). We present mean point estimates and their uncertainty using half‐eye and stat_interval. Black dots are observations accompanied by prediction intervals (Brewer scale). (C) Contrast for CD31 labeled area in defined ROIs of PDGFR*β* ^KLF4‐KO^ mice and corresponding controls for the linear model described in (B). We show posterior draws (*spread_draws*) with *stat_halfeye()* and ROPE. (D) Representative image from Picosirious staining in the ischemic cortex. Total collagen is visible as a red staining over a yellowish background. (E) Picosirious‐stained images in the striatum and cortex from healthy and ischemic stroke animals (14 DPI). Images are shown showing the Red‐Hot LUT in FIJI. (F) Fitted model for the Picosirious‐labeled area (Area*~*Region × Genotype, family = student) for the comparison between PDGFR*β* ^KLF4‐KO^ and KLF4^Flox^ mice at 14 DPI. We display posterior estimates with conditional_effects (mean + 95% CI). High‐resolution figure: https://osf.io/jxb4z/files/cxw6b.

**Table S1:** Hemispheric area following MCAo. (A) Posterior summary with parameter estimates (Est.) and 95% credible intervals (2.5%–97.5%) for the non‐linear (splines) model Area*~*s(DPI, *K* = 5), sigma*~*DPI, family = student. Parameters for sigma are in the scale. (B) Model derivatives (estimate_smooth) showing linear changes from 0 to 23 DPI and 23 to 30 DPI.
**Table S2:** PDGFRβ integrated density following MCAo. (A) Posterior summary with parameter estimates (Est.) and 95% credible intervals (2.5%–97.5%) for the non‐linear (splines) model IntDen~s(DPI, K = 5), sigma~DPI, family = student. Parameters for sigma are in the scale. (B) Model derivatives (estimate_smooth) showing linear changes from 0 to 23 DPI and 23–30 DPI.
**Table S3:** PDGFRβ integrated density following MCAo (conditioning on hemispheric area). (A) Posterior summary with parameter estimates (Est.) and 95% credible intervals (2.5%–97.5%) for the non‐linear (splines) model Pdgfrb_IntDen~t2(DPI,Tissue_Area), family = Gaussian.
**Table S4:** GFAP integrated density following MCAo. (A) Posterior summary with parameter estimates (Est.) and 95% credible intervals (2.5%–97.5%) for the non‐linear (splines) model IntDen~s(DPI, K = 5), sigma~DPI, family = student. Parameters for sigma are shown in the scale. (B) Model derivatives (estimate_smooth) showing linear changes from 0 to 16 DPI and 16–30 DPI.
**Table S5:** GFAP integrated density following MCAo (conditioning on hemispheric area). Posterior summary with parameter estimates (Est.) and 95% credible intervals (2.5%–97.5%) for the non‐linear (splines) model Gfap_IntDen~t2(DPI,Tissue_Area), family = Gaussian.
**Table S6:** PDGFRβhigh likelihood following MCAo. Posterior summary with parameter estimates (Est.) and 95% credible intervals (2.5%–97.5%) for the binomial model Pdgfrb_high|trials (Pdgfrb_Total)~s(DPI, k = 5), family = binomial.
**Table S7:** GFAP convex hull following MCAo. Posterior summary with parameter estimates (Est.) and 95% credible intervals (2.5%–97.5%) for the linear model Healthy_Ratio~DPI, family = student.
**Table S8:** PDGFRβ counts in tesselated GFAP regions. (A) Posterior summary with parameter estimates (Est.) and 95% credible intervals (2.5%–97.5%) for the model Pdgfrb~Region × Gfap, family = hurdle_lognormal(). (B–D) Posterior summary for models by region Pdgfrb~DPI × Gfap, family = hurdle_lognormal().
**Table S9:** PDGFRβhigh likelihood in striatal‐only lesions. Posterior summary with parameter estimates (Est.) and 95% credible intervals (2.5%–97.5%) for the binomial model Pdgfrb_high|trials(Pdgfrb_Total)~DPI, family = binomial.
**Table S10:** Parenchymal PDGFRβ likelihood following MCAo. (A–C) Posterior summary with parameter estimates (Est.) and 95% credible intervals (2.5%–97.5%) for the binomial models Pdgfrb_Parenchymal|trials (Pdgfrb_Total)~DPI, family = binomial for perilesion, sriatum and cortex, respectively.
**Table S11:** FACS‐sorted PDGFRβ+ cells derived from contralateral and ipsilateral hemispheres. (A, B) Posterior summary with parameter estimates (Est.) and 95% credible intervals (2.5%–97.5%) for the linear models Cells~Hemisphere, family = student estimating the percentage of total PDGFRβ+ cells (A) and dead PDGFRβ+ cells (B).
**Table S12:** Parenchymal PDGFRβ likelihood in striatal‐restricted lesions. Posterior summary with parameter estimates (Est.) and 95% credible intervals (2.5%–97.5%) for the binomial model Pdgfrb_Parenchymal|trials (Pdgfrb_Total)~DPI, family = binomial in striatal‐only lesions.
**Table S13:** Number of PDGFRβ+ cells in striatal‐only and cortico‐striatal lesions. Posterior summary with parameter estimates (Est.) and 95% credible intervals (2.5%–97.5%) for the model Pdgfrb_Total~DPI × Lesion, sigma~DPI × Lesion, family = student in striatal‐only lesions.
**Table S14:** Number of parenchymal PDGFRβ+ cells in sham and MCAo mice. Posterior summary with parameter estimates (Est.) and 95% credible intervals (2.5%–97.5%) for the model Parenchymal~DPI × Condition, family = student in sham and MCAo mice.
**Table S15:** PDGFRβ+ cells proliferation in the ischemic hemisphere following MCAo. (A) Posterior summary with parameter estimates (Est.) and 95% credible intervals (2.5%–97.5%) for the non‐linear (splines) model Percentage_Pdgfrb~s(DPI_Cont, k = 5), family = lognormal. (B) Model derivatives (estimate_smooth) showing linear changes from 0–3 DPI and 3–30 DPI.
**Table S16:** PDGFRβ+ cells proliferation in defined ROIs following MCAo. (A) Posterior summary with parameter estimates (Est.) and 95% credible intervals (2.5%–97.5%) for the model Cells~DPI × Region, family = negbinomial showing the total amount of colocalized cells. (B) The same estimates for the model Vascular|trials (Total)~DPI × Region, family = binomial showing the proportion of vascular PDGFRβ+ cells relative to the total number of PDGFRβ+ cells.
**Table S17:** Haralick features of PDGFRβ+ cells. Posterior summary with parameter estimates (Est.) and 95% credible intervals (2.5%–97.5%) for the logistic model Region~entropy + contrast + IDM, family = categorical.
**Table S18:** PDGFRβ stained area in defined ROIs. Posterior summary with parameter estimates (Est.) and 95% credible intervals (2.5%–97.5%) for the binomial model Area~DPI_cont × Region, family = student.
**Table S19:** Topological assessment of PDGFRβ+ cells following injury: Wasserstein (A, B) and Bottleneck (C, D) distances for 0‐ and 1‐dimension homology. We boostraped the estimates (1000 replications) to show the median Betti curve distance with 95% credible intervals (2.5%–97.5%).
**Table S20:** Spatial intensity of KLF4+ nuclei following injury. Posterior summary with parameter estimates (Est.) and 95% credible intervals (2.5%–97.5%) for the model Intensity~0 + DPI, sigma~0 + DPI, family = student. Note that the sigma coefficients are in the log scale.
**Table S21:** Staining intensity of KLF4+ nuclei following injury. Population‐level posterior summary with parameter estimates (Est.) and 95% credible intervals (2.5%–97.5%) for the multilevel model Intensity~0 + DPI + (1|MouseID), family = hurdle_lognormal. Note that the group‐level (mouse‐level) coefficients are shown in the respective QN.
**Table S22:** Staining intensity of KLF4+ nuclei relative to the x‐coordinates. Population‐level posterior summary with parameter estimates (Est.) and 95% credible intervals (2.5%–97.5%) for the multilevel model Intensity~0 + DPI × Scaled_CenterX + (1|MouseID), family = lognormal. Note that the group‐level (mouse‐level) coefficients are shown in the respective QN.
**Table S23:** KLF4/PDGFRβ co‐localization in defined ROIs. Posterior summary with parameter estimates (Est.) and 95% credible intervals (2.5%–97.5%) for the model Coloc|trials(Klf4)~DPI × Region, family = binomial.
**Table S24:** Spatial intensity of KLF4+ nuclei in PDGFRβKLF4‐KO mice following injury. Posterior summary with parameter estimates (Est.) and 95% credible intervals (2.5%–97.5%) for the model Intensity~Genotype, sigma~Genotype, family = student.
**Table S25:** Hemispheric ratio in PDGFRβKLF4‐KO mice. Posterior summary with parameter estimates (Est.) and 95% credible intervals (2.5%–97.5%) for the model Shrinkage~Genotype, family = student.
**Table S26:** GFAP integrated density in PDGFRβKLF4‐KO mice. Posterior summary with parameter estimates (Est.) and 95% credible intervals (2.5%–97.5%) for the model Gfap~Genotype, family = student.
**Table S27:** PDGFRβ integrated density in PDGFRβKLF4‐KO mice. Posterior summary with parameter estimates (Est.) and 95% credible intervals (2.5%–97.5%) for the model Pdgfrb~Genotype, family = student.
**Table S28:** PDGFRβ integrated density in PDGFRβKLF4‐KO mice with additional predictors. Posterior summary with parameter estimates (Est.) and 95% credible intervals (2.5%–97.5%) for the models (a) Pdgfrb~Genotype × Shrinkage, family = student and (b) Pdgfrb~Genotype × Ipsilateral_Scaled, family student.
**Table S29:** PDGFRβ and GFAP integrated density in PDGFRβKLF4‐KO mice (intraperitoneal injection prior to ischemia). Posterior summary with parameter estimates (Est.) and 95% credible intervals (2.5%–97.5%) for the models (a) Pdgfrb~Genotype, family = student and (b) Gfap~Genotype, family student.
**Table S30:** Number of PDGFRβ+ nuclei in PDGFRβKLF4‐KO mice. Posterior summary with parameter estimates (Est.) and 95% credible intervals (2.5%–97.5%) for the multilevel model (a) Counts~Genotype + (1|MouseID), family = negbinomial. The values are presented in the log scale.
**Table S31:** PDGFRβ+ labeling intensity in PDGFRβKLF4‐KO mice. Posterior summary with parameter estimates (Est.) and 95% credible intervals (2.5%–97.5%) for the multilevel model (a) Intensity~Area × Genotype + (1|MouseID), family = student.
**Table S32:** Number of PDGFRβ+ nuclei in PDGFRβCre and KLF4Flox mice. Posterior summary with parameter estimates (Est.) and 95% credible intervals (2.5%–97.5%) for the multilevel model Cells~Genotype + (1|MouseID), family = student.
**Table S33:** PDGFRβ+ labeling intensity in PDGFRβCre and KLF4Flox mice. Posterior summary with parameter estimates (Est.) and 95% credible intervals (2.5%–97.5%) for the multilevel model Intensity~Area × Genotype + (1|MouseID), family = student.
**Table S34:** Collagen‐IV (ColIV) labeling intensity in PDGFRβKLF4‐KO mice. Posterior summary with parameter estimates (Est.) and 95% credible intervals (2.5%–97.5%) for the model Intensity~Genotype, family = student.
**Table S35:** CD31 area in PDGFRβKLF4‐KO mice. Posterior summary with parameter estimates (Est.) and 95% credible intervals (2.5%–97.5%) for the model Area~Genotype, sigma~Genotype, family = student.
**Table S36:** ColIV area in PDGFRβKLF4‐KO mice. Posterior summary with parameter estimates (Est.) and 95% credible intervals (2.5%–97.5%) for the model Area~Genotype, family = student.
**Table S37:** ColIV area in PDGFRβKLF4‐KO mice using Picrosirius staining. Posterior summary with parameter estimates (Est.) and 95% credible intervals (2.5%–97.5%) for the model Area~Genotype, family = student.

**Data S1:** Supplementary Method 1. Analysis of the hemispheric shrinkage.
Supplementary Method 2: Analysis of the reactivity (Integrated density) of PDGFR*β* ^tdTomato^ and GFAP^+^ cells in the ipsilateral hemisphere.
Supplementary Method 3: Analysis of PDGFR*β* ^high^ and PDGFR*β* ^low^ subpopulations in the ipsilateral hemisphere.
Supplementary Method 4: Point pattern analysis (PPA) for PDGFR*β* ^+^ cells and GFAP^+^ cells in the ipsilateral hemisphere.
Supplementary Method 5: Analysis of GFAP^+^ cell convex hull in the ipsilateral hemisphere.
Supplementary Method 6: Analysis of PDGFR*β* ^+^ cell count in tessellated GFAP^+^ areas in the ipsilateral hemisphere.
Supplementary Method 7: Analysis of perivascular and parenchymal PDGFR*β* ^+^ cells in defined ROIs of the ipsilateral hemisphere.
Supplementary Method 8: Fluorescence‐activated cell sorting (FACS) of brain PDGFR*β* ^+^ cells.
Supplementary Method 9: PPA and colocalization analysis of Ki67^+^/PDGFR*β* ^+^ cells in the ipsilateral hemisphere.
Supplementary Method 10: Analysis of Ki67^+^/PDGFR*β* ^tdTomato^ cells in defined ROIs of the ipsilateral hemisphere.
Supplementary Method 11: Morphological analysis of PDGFR*β* ^+^ cells in defined ROIs of the ipsilateral hemisphere.
Supplementary Method 12: Analysis of covered area and Haralick features (texture) of PDGFR*β* ^+^ cells in defined ROIs of the ipsilateral hemisphere.
Supplementary Method 13: Topological data analysis (TDA) of PDGFR*β* ^+^ cells in defined ROIs of the ipsilateral hemisphere.
Supplementary Method 14: Spatial and labeling intensity analysis of KLF4 in the ipsilateral hemisphere.
Supplementary Method 15: Analysis of PDGFR*β* ^+^/KLF4^+^ cells in defined ROIs of the ipsilateral hemisphere.
Supplementary Method 16: Analysis of KLF4 expression in PDGFR*β* ^KLF4‐KO^ mice.
Supplementary Method 17: Analysis of brain shrinkage and PDGFR*β*/GFAP expression in PDGFR*β* ^KLF4‐KO^ mice.
Supplementary Method 18: Analysis of PDGFR*β* mRNA transcript spatial expression in the brain of PDGFR*β* ^KLF4‐KO^ mice.
Supplementary Method 19: Analysis of PDGFR*β* mRNA transcript spatial expression in the brain of PDGFR*β* ^KLF4‐KO^, KLF4^Flox^, and PDGFR*β* ^Cre^ mice.
Supplementary Method 20: Analysis of Collagen IV (ColIV) in PDGFR*β* ^KLF4‐KO^ mice.

**Video S1:** Representative animation of Vietoris‐Rips complexes.

## Data Availability

All the data associated with the publication are made fully available via https://zenodo.org/records/10553084, which includes original microscopy images stored in Zenodo (10.5281/zenodo.10553084) as well as access to complementary data stored in Open Science Framework (OSF) repository (10.17605/OSF.IO/74MQN), and reproducible workflows for image processing and analysis are available on GitHub (10.5281/zenodo.13948420).
