# Supplementary material for: Brain PDGFRβ + Cells Exhibit Diverse Reactive Phenotypes After Stroke Without Requiring KLF4
Source: Glia. 2026 Oct 31;75(1):e70232. doi: 10.1002/glia.70232 (PMC13723038; doi:10.1002/glia.70232)
Supplement: Supplementary file 1 [file GLIA-75-0-s001.docx]

**Supplementary Methods**

Supplementary Method 1: Analysis of the hemispheric shrinkage	3

Supplementary Method 2: Analysis of the reactivity (Integrated density) of PDGFRβ^tdTomato^ and GFAP^+^ cells in the ipsilateral hemisphere	4

Supplementary Method 3: Analysis of PDGFRβ^high^ and PDGFRβ^low^ subpopulations in the ipsilateral hemisphere	6

Supplementary Method 4: Point pattern analysis (PPA) for PDGFRβ^+^ cells and GFAP^+^ cells in the ipsilateral hemisphere	8

Supplementary Method 5: Analysis of GFAP^+^ cell convex hull in the ipsilateral hemisphere	10

Supplementary Method 6: Analysis of PDGFRβ^+^ cell count in tessellated GFAP^+^ areas in the ipsilateral hemisphere	11

Supplementary Method 7: Analysis of perivascular and parenchymal PDGFRβ^+^ cells in defined ROIS of the ipsilateral hemisphere	13

Supplementary Method 8: Fluorescence-activated cell sorting (FACS) of brain PDGFRβ^+^ cells	16

Supplementary Method 9: PPA and colocalization analysis of Ki67^+^ / PDGFRβ^+^ cells in the ipsilateral hemisphere	18

Supplementary Method 10: Analysis of Ki67^+^ / PDGFRβ^tdTomato^ cells in defined ROIs of the ipsilateral hemisphere	20

Supplementary Method 11: Morphological analysis of PDGFRβ^+^ cells in defined ROIs of the ipsilateral hemisphere	22

Supplementary Method 12: Analysis of covered area and Haralick features (texture) of PDGFRβ^+^ cells in defined ROIs of the ipsilateral hemisphere	24

Supplementary Method 13: Topological data analysis (TDA) of PDGFRβ^+^ cells in defined ROIs of the ipsilateral hemisphere	27

Supplementary Method 14: Spatial and labeling intensity analysis of KLF4 in the ipsilateral hemisphere	29

Supplementary Method 15: Analysis of PDGFRβ^+^ / KLF4^+^ cells in defined ROIs of the ipsilateral hemisphere	32

Supplementary Method 16: Analysis of KLF4 expression in PDGFRβ^KLF4-KO^ mice	34

Supplementary Method 17: Analysis of brain shrinkage and PDGFRβ / GFAP expression in PDGFRβ^KLF4-KO^ mice	36

Supplementary Method 18: Analysis of PDGFRβ mRNA transcript spatial expression in the brain of PDGFRβ^KLF4-KO^ mice	38

Supplementary Method 19: Analysis of PDGFRβ mRNA transcript spatial expression in the brain of PDGFRβ^KLF4-KO^, KLF4^Flox^, and PDGFRβ^Cre^ mice	40

Supplementary Method 20: Supplementary Method 20: Analysis of Collagen IV (ColIV) in PDGFRβ^KLF4-KO^ mice	41

Herein, we provide further details on the statistical modeling and analysis methods for the article **“Brain PDGFRβ^+^ cells exhibit diverse reactive phenotypes after stroke without requiring KLF4”**. The raw images and data are available in Zenodo ([10.5281/zenodo.10553084](https://zenodo.org/uploads/10553084)) and Open Science Framework (OSF) ([10.17605/OSF.IO/74MQN](https://osf.io/74mqn/)) repositories. Note that some resources are not rendered in the OSF repository and users should download them to access the content. Likewise, the complete analysis pipelines in R (Quarto notebooks) or Python (Jupyter notebooks) are available in the GitHub repository (<https://github.com/elalilab/Stroke_PDGFR-B_Reactivity>).

Otherwise stated, we analyzed data by fitting case-specific Bayesian linear and non-linear regression models using the brms R package (Bürkner, 2017). For point pattern analysis (PPA), we employed the spatstat package (Baddeley et al., 2005) and associated multiple point process models (ppm) and relative distribution (rhohat) functions. Given the heterogeneity of our approaches, we thoroughly describe data handling and statistical modeling (including priors, model validation, and model comparison) in fully annotated [Quarto](https://quarto.org/) notebooks (QNs). Moreover, we used the [modelsummary](https://modelsummary.com/) package to write latex (.tex) and .html summary tables displaying model results. This material is available in the “[Tables/tex](https://osf.io/e7gwm/)” and “[Tables/html](https://osf.io/e7gwm/)” components of the OSF repository. In the following sections, we provide a tailored description for the analysis of each dataset. We remark that this content contains direct links to single files (i.e. [Widefield_5x_Ipsilateral_Gfap-Pdgfrb.zip](https://osf.io/jrzq5)) hosted in the Zenodo or OSF repositories, or routes to the OSF components (Datasets, Images_Processing) and specific folders when referring to several files (i.e. [StatisticalModels_brms/Widefield_5x_Ipsilateral_Gfap-Pdgfrb_Shrinkage](https://osf.io/zvnxa/)). We note that we have had problems running brms in the last version of R and are unaware if these issues still exist. Be aware that we employed R 4.1.2 and the last available versions of all R-packages. Please check the sessionInfo() in each QN for further details. We also expect warnings opening QuPath projects, Ilastik, and CellProfiller pipelines because of file paths. We provide all the required files in our repositories to reproduce our results, but the user must download the material and change the file path accordingly. This does not apply to QN, JN, and .csv files if the user establishes an R-project hosting the files. Do not hesitate to raise an issue on [GitHub](https://github.com/elalilab/Stroke_PDGFR-B_Reactivity/issues) or contact the research team if some issues arise.

**Supplementary Method 1: Analysis of the hemispheric shrinkage.**

| **Component** | **Link** |
| --- | --- |
| Figures | Figure 2A-B, Suppl. Figure 1D-E |
| Raw Images | [Widefield_5x_Ipsilateral_Gfap-Pdgfrb.zip](https://zenodo.org/api/records/10553084/draft/files/Widefield_5x_Ipsilateral_Gfap-Pdgfrb.zip/content) |
| Low-resolution images | [Widefield_5x_Ipsilateral_Gfap-Pdgfrb.zip](https://osf.io/jrzq5) |
| Image Processing | [Images_Processing/Widefield_5x_Ipsilateral_Gfap-Pdgfrb](https://osf.io/9zdc7/) |
| Raw Data | [Raw_Widefield_5x_Ipsilateral_Gfap_Str_QuPathAnnotations.csv](http://raw_widefield_5x_ipsilateral_gfap_str_qupathannotations.csv) |
| Processed data | [Widefield_5x_Ipsilateral_Gfap-Pdgfrb_Inten.csv](https://osf.io/3t8b2) |
| Statistical models | [StatisticalModels_brms/Widefield_5x_Ipsilateral_Gfap-Pdgfrb_Shrinkage](https://osf.io/zvnxa/) |
| Analysis notebook | [Widefield_5x_Ipsilateral_Gfap-Pdgfrb_Handling.qmd](https://github.com/elalilab/Stroke_PDGFR-B_Reactivity/blob/main/Widefield_5x_Ipsilateral_Gfap-Pdgfrb_Handling.qmd)  [Widefield_5x_Ipsilateral_Pdgfrb_Shrinkage.qmd](https://github.com/elalilab/Stroke_PDGFR-B_Reactivity/blob/main/Widefield_5x_Ipsilateral_Pdgfrb_Shrinkage.qmd) |

We acquired widefield-stitched [images](https://osf.io/jrzq5) at 5x magnification of the ipsilateral hemisphere of PDGFRβ^tdTomato^ mice to analyze hemispheric shrinkage (atrophy). Using a CellProfiler [script](https://osf.io/6vtn8), we quantified the hemispheric area in an unbiased manner. We cleaned and processed the raw data as specified in [QN](https://github.com/elalilab/Stroke_PDGFR-B_Reactivity/blob/main/Widefield_5x_Ipsilateral_Gfap-Pdgfrb_Handling.qmd). The resulting [dataset](https://osf.io/3t8b2) includes the variables analyzed in the following sections: Analysis of cell reactivity (Integrated density) for PDGFRβ and GFAP^+^ and Analysis of PDGFRβhigh and PDGFRβlow populations. For the analysis of brain shrinkage, we fitted various statistical models to the data, and we made model selection aided by the Watanabe-Akaike Information Criterion (WAIC) or Leave-one-out (Loo) cross-validation (Vehtari et al., 2016). We performed statistical inference based on a [splines model](https://osf.io/z6qus) (with heteroskedasticity):

$$Shrinkage \sim s(DPI, k=5), sigma \sim DPI, family = student$$

We calculated the corresponding linear derivatives to interpret the effects of DPI using [modelbased](https://easystats.github.io/modelbased/) package [(Makowski et al., 2020)](https://www.zotero.org/google-docs/?wf4bCN) as specified in the [QN](https://github.com/elalilab/Stroke_PDGFR-B_Reactivity/blob/main/Widefield_5x_Ipsilateral_Pdgfrb_Shrinkage.qmd). We share the [raw model](https://osf.io/wup8e) summary or [derivatives](https://osf.io/ksrdu) as .tex tables in “[Tables/tex](https://osf.io/e7gwm/)” component of the OSF repository. For the visualization of the results, we obtained posterior draws using the fitted function from stats package and generated a [line graph](https://osf.io/fr29g) using stat_lineribbon() with 0.5, 0.8, and 0.95 posterior credible intervals (CI) (blue Brewer scale).

**Supplementary Method 2: Analysis of the reactivity (Integrated density) of PDGFRβ^tdTomato^ and GFAP^+^ cells in the ipsilateral hemisphere.**

| **Component** | **Link** |
| --- | --- |
| Figures | Figure 2C-E and Suppl. Figure 1A-E |
| Raw Images | [Widefield_5x_Ipsilateral_Gfap-Pdgfrb.zip](https://zenodo.org/api/records/10553084/draft/files/Widefield_5x_Ipsilateral_Gfap-Pdgfrb.zip/content) |
| Low-resolution images | [Widefield_5x_Ipsilateral_Gfap-Pdgfrb.zip](https://osf.io/jrzq5) |
| Image Processing | [Images_Processing/Widefield_5x_Ipsilateral_Gfap-Pdgfrb](https://osf.io/9zdc7/) |
| Raw Data | [Raw_Widefield_5x_Ipsilateral_Gfap-Pdgfrb_AreaIntensity.csv](https://osf.io/6a3t4) |
| Processed data | [Widefield_5x_Ipsilateral_Gfap-Pdgfrb_Inten.csv](https://osf.io/3t8b2) |
| Statistical models | [StatisticalModels_brms/Widefield_5x_Ipilateral_Gfap-Pdgfrb_Inten](https://osf.io/zvnxa/) |
| Analysis notebook | [Widefield_5x_Ipsilateral_Pdgfrb_IntDen.qmd](https://github.com/elalilab/Stroke_PDGFR-B_Reactivity/blob/main/Widefield_5x_Ipsilateral_Pdgfrb_IntDen.qmd)  [Widefield_5x_Ipsilateral_Gfap_IntDen.qmd](https://github.com/elalilab/Stroke_PDGFR-B_Reactivity/blob/main/Widefield_5x_Ipsilateral_Gfap_IntDen.qmd) |

We analyzed widefield-stitched [images](https://osf.io/jrzq5) at 5x magnification of PDGFRβ^tdTomato^/GFAP^+^ immunolabeling in brain sections (see the [protocol](https://osf.io/d2un3/files/79v2c)) to quantify the integrated density (IntDen) (mean intensity x labeled area) of [PDGFRβ](https://github.com/elalilab/Stroke_PDGFR-B_Reactivity/blob/main/Widefield_5x_Ipsilateral_Pdgfrb_IntDen.qmd) and [GFAP](https://github.com/elalilab/Stroke_PDGFR-B_Reactivity/blob/main/Widefield_5x_Ipsilateral_Gfap_IntDen.qmd). We made the measurements in the ipsilateral hemisphere using a FIJI [script](https://osf.io/mqukx) and cleaned the [data](https://osf.io/sx8fv) for analysis as specified in the [QN](https://github.com/elalilab/Stroke_PDGFR-B_Reactivity/blob/main/Widefield_5x_Ipsilateral_Gfap-Pdgfrb_Handling.qmd). Based on WAIC, we made statistical inferences with a [spline model](https://osf.io/ag87j) that included heteroskedasticity (the estimation of sigma given different group variance):

$$IntDen \sim s(DPI, k=5), sigma \sim DPI, family = student$$

We calculated the corresponding linear derivatives as described previously. We share the [raw model](https://osf.io/gf9rd) summary or [derivatives](https://osf.io/67t3d) as .tex tables in the “[Tables/tex](https://osf.io/e7gwm/)” component of the OSF repository. We visualized the [results](https://osf.io/hqu39) using the conditional_effects function from brms with additional ggplot aesthetics. Additionally, we investigated the relationship between integrated density (IntDen) and hemispheric area (Tissue_Area) using the [spline model](https://osf.io/kyqnc):

$$IntDen \sim t2(DPI, TisseArea), family =guassian$$

The [results](https://osf.io/56q9f) are shown using the conditional_smooths function from brms with a Viridis scale. Please note that links in this section are for PDGFRβ. Analogous resources for GFAP are available in the OSF repository.

**Supplementary Method 3: Analysis of PDGFRβ^high^ and PDGFRβ^low^ subpopulations in the ipsilateral hemisphere.**

| **Component** | **Link** |
| --- | --- |
| Figures | Figure 2F-G, Suppl. Figure 1F. |
| Raw Images | [Widefield_5x_Ipsilateral_Gfap-Pdgfrb.zip](https://zenodo.org/api/records/10553084/draft/files/Widefield_5x_Ipsilateral_Gfap-Pdgfrb.zip/content) |
| Low-resolution images | [Widefield_5x_Ipsilateral_Gfap-Pdgfrb.zip](https://osf.io/jrzq5) |
| Image Processing | [Images_Processing/Widefield_5x_Ipsilateral_Gfap-Pdgfrb](https://osf.io/9zdc7/) |
| Raw Data | [Raw_Widefield_5x_Ipsilateral_Pdgfrb_QuPathAnnotations.csv](https://osf.io/7nyza)  [Raw_Widefield_5x_Ipsilateral_Pdgfrb_Str_QuPathAnnotations.csv](https://osf.io/u7zr2) |
| Processed data | [Widefield_5x_Ipsilateral_Gfap-Pdgfrb_Inten.csv](https://osf.io/3t8b2)  [Widefield_5x_Ipsilateral_Gfap-Pdgfrb_Inten_Str.csv](https://osf.io/rxk9f) |
| Statistical models | [StatisticalModels_brms/Widefield_5x_Ipsilateral_Pdgfrb_LowHigh](https://osf.io/zvnxa/) |
| Analysis notebook | [Widefield_5x_Ipsilateral_Pdgfrb_LowHigh.qmd](https://github.com/elalilab/Stroke_PDGFR-B_Reactivity/blob/main/Widefield_5x_Ipsilateral_Pdgfrb_LowHigh.qmd)  [Widefield_5x_Ipsilateral_Pdgfrb_LowHigh_Str.qmd](https://github.com/elalilab/Stroke_PDGFR-B_Reactivity/blob/main/Widefield_5x_Ipsilateral_Pdgfrb_LowHigh_Str.qmd) |

We acquired widefield-stitched [images](https://osf.io/jrzq5) at 5x magnification of sections in the ipsilateral hemisphere of PDGFRβ^tdTomato^ mice to analyze the populations of PDGFRβ^high^ and PDGFRβ^low^ cells. We unbiasedly detected and quantified cells using QuPath (Bankhead et al., 2017). We loaded the images into a [QuPath project](https://osf.io/8ehyu) and processed the files using a.groovy batch processing [script](https://osf.io/6vtn8). This pipeline makes use of machine learning-based classifiers (available in the [QuPath project](https://osf.io/8ehyu) folder in the OSF repository) that label typical PDGFRβ cells as non-reactive (PDGFRβ^low^) or increased PDGFRβ expression as reactive cells (PDGFRβ^high^). We handled the raw data using the same [QN](https://github.com/elalilab/Stroke_PDGFR-B_Reactivity/blob/main/Widefield_5x_Ipsilateral_Gfap-Pdgfrb_Handling.qmd) employed for brain shrinkage and exported the results to the same [dataset](https://osf.io/3t8b2). To analyze cell proportions, we fitted Bayesian models using a binomial family distribution, where the response variable represents a series of Bernoulli trials (PDGFRβ^high^ or PDGFRβ^low^ cells) in a fixed number of independent trials (PDGFRβ^total^). For additional details concerning this modeling strategy, validation, and model comparison, please refer to the associated [QN](https://github.com/elalilab/Stroke_PDGFR-B_Reactivity/blob/main/Widefield_5x_Ipsilateral_Pdgfrb_LowHigh.qmd). We performed statistical inference using a [spline model](https://osf.io/nkgsm):

$$PDGFR\beta_{High} | trials(PDGFR\beta_{Total} \sim s(DPI, k=5), family = binomial$$

We visualized the [results](https://osf.io/rq2ad) using the conditional_effects function from brms with additional ggplot aesthetics and shared the model summary as a .tex [table](https://osf.io/6uc85) in the OSF repository. Please note that the [modelbased](https://easystats.github.io/modelbased/) package does not have functions to generate linear derivatives from binomial models.

We performed the same procedure for an image set derived from mice with only striatal lesions. We generated a different [Qupath project](https://osf.io/ty4z5) because the staining and imaging for these animals were done on a different day. The analysis pipeline is detailed in the [QN](https://github.com/elalilab/Stroke_PDGFR-B_Reactivity/blob/main/Widefield_5x_Ipsilateral_Pdgfrb_LowHigh_Str.qmd) and all the associated resources are analogously present in the OSF repository.

## **Supplementary Method 4: Point pattern analysis (PPA) for PDGFRβ^+^ cells and GFAP^+^ cells in the ipsilateral hemisphere.**

| **Component** | **Link** |
| --- | --- |
| Figures | Figure 1H-I, Suppl. Figure 1F, Suppl. Figure 2A,F |
| Raw Images | [Widefield_5x_Ipsilateral_Gfap-Pdgfrb.zip](https://zenodo.org/api/records/10553084/draft/files/Widefield_5x_Ipsilateral_Gfap-Pdgfrb.zip/content) |
| Low-resolution images | [Widefield_5x_Ipsilateral_Gfap-Pdgfrb.zip](https://osf.io/jrzq5) |
| Image Processing | [Images_Processing/Widefield_5x_Ipsilateral_Gfap-Pdgfrb](https://osf.io/9zdc7/) |
| Raw Data | [Widefield_5x_Ipsilateral_Gfap-Pdgfrb_QuPath.zip](https://osf.io/8ehyu)  [Widefield_5x_Ipsilateral_Gfap-Pdgfrb_QuPath_Str.zip](https://osf.io/ty4z5) |
| Processed data | [Widefield_5x_Ipsilateral_Gfap-Pdgfrb-Dapi_Coordinates.zip](https://osf.io/ybw26)  [Widefield_5x_Ipsilateral_Gfap-Pdgfrb-Dapi_Str_Coordinates.zip](https://osf.io/bje4d) |
| Point patterns | [Widefield_5x_Ipsilateral_Pdgfrb-Gfap_PPP.rds](https://osf.io/ab4y5)  [Widefield_5x_Ipsilateral_Pdgfrb-Gfap_Str_PPP.rds](https://osf.io/2c5fj) |
| Statistical models | N/A - mppm and rhohat models |
| Analysis notebook | [Widefield_5x_Ipsilateral_Gfap-Pdgfrb_Covariance.qmd](https://github.com/elalilab/Stroke_PDGFR-B_Reactivity/blob/main/Widefield_5x_Ipsilateral_Gfap-Pdgfrb_Covariance.qmd)  [Widefield_5x_Ipsilateral_Gfap-Pdgfrb_Covariance_Str.qmd](https://github.com/elalilab/Stroke_PDGFR-B_Reactivity/blob/main/Widefield_5x_Ipsilateral_Gfap-Pdgfrb_Covariance_Str.qmd) |

We acquired widefield-stitched [images](https://osf.io/8u6kt) at 5x magnification of PDGFRβ^tdTomato^/GFAP^+^ immunolabeling in brain sections (see the [protocol](https://osf.io/d2un3/files/79v2c)) to analyze the distribution and covariance of PDGFRβ^+^ and GFAP^+^ cells in the ischemic hemisphere. We used QuPath (including machine-learning classifiers) to detect [PDGFRβ](https://osf.io/6vtn8)^tdTomato^, [GFAP](https://osf.io/9dm2n)^+^, and [DAPI](https://osf.io/xjt7z)^+^ cells and obtain single-cell *xy* coordinates. DAPI was used only to define the observation window of PDGFRβ and GFAP point patterns. We handled the raw data to save cell coordinates (per mouse) in individual .csv [files](https://osf.io/ybw26) for further processing (see [QN](https://github.com/elalilab/Stroke_PDGFR-B_Reactivity/blob/main/Widefield_5x_Ipsilateral_Gfap-Pdgfrb_Handling.qmd)). Subsequently, we created point patterns using the ppp function from spatstat as specified in the [QN](https://github.com/elalilab/Stroke_PDGFR-B_Reactivity/blob/main/Widefield_5x_Ipsilateral_Gfap-Pdgfrb_Handling.qmd), and stored them as .rds (R-software) [hyper-frames](https://osf.io/ab4y5) with their respective grouping variables. These objects are available in the OSF repository to encourage their reuse for validation, research, or educational purposes.

We used spatstat-associated functions to generate density kernels (0.2 sigma) and conduct PPA (see [QN](https://github.com/elalilab/Stroke_PDGFR-B_Reactivity/blob/main/Widefield_5x_Ipsilateral_Gfap-Pdgfrb_Covariance.qmd)). Then, we used rhohat function to calculate the relative spatial intensity of PDGFRβ^+^ cells with respect to the density kernels of GFAP^+^ cells. In this context, spatial intensity refers to the number of objects per unit area, while maintaining all the spatial information available. Importantly, we passed the rhohat function with the argument do.CI = FALSE to allow further pooling per DPI with the pool function. The source code for generating density kernel plots and graphs of relative distribution **(as shown in Figure 1H-I and Suppl. Figure 1F)** are available in the [QN](https://github.com/elalilab/Stroke_PDGFR-B_Reactivity/blob/main/Widefield_5x_Ipsilateral_Gfap-Pdgfrb_Covariance.qmd).

Next, we fitted a spatial model for replicated point patterns using the mppm function from spatstat. This function assumes that the points follow an inhomogeneous Poisson process with an intensity function λ that varies over space μ. Please see the [QN](https://github.com/elalilab/Stroke_PDGFR-B_Reactivity/blob/main/Widefield_5x_Ipsilateral_Gfap-Pdgfrb_Covariance.qmd) for further details. We fitted nested models for PDGFRβ^high^ (Figure 1I-J) and PDGFRβ^low^ **(Suppl. Figure 1H)**:

$$PDGFR\beta\sim DPI/GFAP Kernel, family = Poisson$$

The estimates with 95% confidence intervals (output on the log scale) are available in Supplementary Tables but not as a .tex table in the OSF repository. We performed an analogous procedure for mice with only [striatal lesions](https://github.com/elalilab/Stroke_PDGFR-B_Reactivity/blob/main/Widefield_5x_Ipsilateral_Gfap-Pdgfrb_Covariance_Str.qmd) (as explained previously).

## **Supplementary Method 5: Analysis of GFAP^+^ cell convex hull in the ipsilateral hemisphere.**

| **Component** | **Link** |
| --- | --- |
| Figures | Suppl. Figure 1F-G |
| Raw Images | [Widefield_5x_Whole_Gfap-Pdgfrb.zip](https://zenodo.org/api/records/10553084/draft/files/Widefield_5x_Whole_Gfap-Pdgfrb.zip/content) |
| Low-resolution images | [Widefield_5x_Whole_Gfap-Pdgfrb.zip](https://osf.io/8u6kt) |
| Image Processing | [Images_Processing/Widefield_5x_Whole_Gfap-Pdgfrb](https://osf.io/9zdc7/) |
| Raw Data | N/A - Manual measurements |
| Processed data | [Widefield_5x_Whole_Gfap_Coverage.csv](https://osf.io/72rfz) |
| Statistical models | [StatisticalModels_brms/Widefield_5x_Whole_Gfap_Convex](https://osf.io/zvnxa/) |
| Analysis notebook | [Widefield_5x_Ipsilateral_Gfap_Convex.qmd](https://github.com/elalilab/Stroke_PDGFR-B_Reactivity/blob/main/Widefield_5x_Ipsilateral_Gfap_Convex.qmd) |

We analyzed widefield-stitched [images](https://osf.io/8u6kt) at 5x magnification of PDGFRβ^tdTomato^/GFAP^+^ immunolabeling in brain sections to quantify the convex hull area (area covered by GFAP^+^ cells) in the ipsilateral hemisphere. We performed manual measurements of the are covered by GFAP^+^ cells using the standard *freehand section* and *measure* functions in FIJI **(as shown in Suppl. Figure 1G, yellowed encircled area)**. Previous research has shown that this area is fully populated by NeuN^+^ cells and therefore corresponds to viable tissue [(Manrique-Castano et al., 2024)](https://www.zotero.org/google-docs/?p8wWyc). [Dataset](https://osf.io/72rfz) handling and model diagnostics/comparison are specified in the [QN](https://github.com/elalilab/Stroke_PDGFR-B_Reactivity/blob/main/Widefield_5x_Ipsilateral_Gfap_Convex.qmd). We performed statistical inference with a [linear model](https://osf.io/pvwqj):

$$Healthy\_Ratio \sim DPI, family = student$$

We share the summary [table](https://osf.io/y6z3u) and show the [results](https://osf.io/przmb) as a line plot using the same strategy as for brain shrinkage.

## **Supplementary Method 6: Analysis of PDGFRβ^+^ cell count in tessellated GFAP^+^ areas in the ipsilateral hemisphere.**

| **Component** | **Link** |
| --- | --- |
| Figures | Figure 1K, L, Suppl. Figure 2A-B |
| Raw Images | [Widefield_10x_ROIs_Gfap-Pdgfrb.zip](https://zenodo.org/api/records/10553084/draft/files/Widefield_10x_ROIs_Gfap-Pdgfrb.zip/content) |
| Low-resolution images | [Widefield_10x_ROIs_Gfap-Pdgfrb.zip](https://osf.io/qrgws) |
| Image Processing | [Images_Processing/Widefield_10x_ROIs_Gfap-Pdgfrb](https://osf.io/9zdc7/) |
| Raw Data | [Raw_Widefield_10x_ROIs_Gfap-Pdgfrb_Tessellations.csv](https://osf.io/29kpm)  [Widefield_10x_ROIs_Gfap-Pdgfrb_QuPath.zip](https://osf.io/tp6ws)  [Widefield_10x_ROIs_Gfap-Pdgfrb_QuPath_Str.zip](https://osf.io/dsxmb) |
| Processed data | [Widefield_10x_ROIs_Gfap-Pdgfrb-Dapi_Coordinates.zip](https://osf.io/byavc)  [Widefield_10x_ROIs_Gfap-Pdgfrb-Dapi_Coordinates_Excluded.zip](https://osf.io/n5xjb) |
| Point patterns | [Widefield_10x_ROIs_Gfap-Pdgfrb_PPP.rds](https://osf.io/3fymw) |
| Statistical models | [StatisticalModels_brms/Widefield_10x_ROIs_Gfap-Pdgfrb_Covariance](https://osf.io/zvnxa/) |
| Analysis notebook | [Widefield_10x_ROIs_Gfap-Pdgfrb_Handling.qmd](https://github.com/elalilab/Stroke_PDGFR-B_Reactivity/blob/main/Widefield_10x_ROIs_Gfap-Pdgfrb_Handling.qmd)  [Widefield_10x_ROIs_Gfap-Pdgfrb_Covariance.qmd](https://github.com/elalilab/Stroke_PDGFR-B_Reactivity/blob/main/Widefield_10x_ROIs_Gfap-Pdgfrb_Covariance.qmd) |

We acquired widefield [images](https://osf.io/qrgws) at 10x magnification in the injured cortex (Ctx, cortex), injured striatum (Str, striatum), and the healthy perilesional cortex (Peri, perilesion) of PDGFRβ^tdTomato^/GFAP^+^ immunolabeling in brain sections (see the [protocol](https://osf.io/d2un3/files/79v2c)) to analyze the relative distribution of PDGFRβ^+^ conditional on the spatial intensity of reactive astrocytes. We detected [PDGFRβ](https://osf.io/vqd6c)^tdTomato^, [GFAP](https://osf.io/t45fn/)^+^, and [DAPI](https://osf.io/cuk46)^+^ cells using batch processing scripts for QuPath and created [point patterns](https://osf.io/3fymw) using DAPI^+^ cells to establish the observation window. We note that for this dataset we [excluded](https://osf.io/n5xjb) mouse 80 (sham) and mouse 74 (3D), given that no detection of GFAP^+^ cells (*NULL* data) prevented the successful construction of the hyper-frame. We analyzed the covariance of PDGFRβ^+^ and GFAP^+^ cells using GFAP-based tessellations. These are non-overlapping regions segregating areas of low and high GFAP spatial intensity. We built tessellations using two quantiles for GFAP^low^ and GFAP^high^ spatial intensity and the tess function from spatstat. Then, we obtained the number of PDGFRβ^+^ in each region using the quadratcount function as shown in [QN](https://github.com/elalilab/Stroke_PDGFR-B_Reactivity/blob/main/Widefield_10x_ROIs_Gfap-Pdgfrb_Handling.qmd). We employed two modeling strategies for this dataset. First, we fitted a linear [model](https://osf.io/2kfye):

$$PDGFR\beta\sim Region*GFAP, family = hurdle\_lognormal$$

to estimate the number of PDGFRβ^+^ cells conditioning on the interaction between region (perilesion, striatum, or cortex) and GFAP spatial intensity (low, high). We used the hurdle_lognormal family distribution to account for the overdispersion of positive data values containing zeros. Model diagnostics are available in the [QN](https://github.com/elalilab/Stroke_PDGFR-B_Reactivity/blob/main/Widefield_10x_ROIs_Gfap-Pdgfrb_Covariance.qmd). We show the [results](https://osf.io/rdbgt) using conditional_effects for point estimates and their uncertainty and comparisons (emmeans) between regions using stat_slab + stat_pointinterval. The summary [table](https://osf.io/uh7c4) is available in OSF.

In the second place, we could not fit linear models conditioning on the three grouping variables (region, GFAP tessellation, DPI), given that MCMC chains did not converge on a stable posterior distribution. Therefore, we fitted separate models for the [perilesion](https://osf.io/n79hf), [striatum](https://osf.io/dps5k), and [cortex](https://osf.io/mk2xh) with the same model formula and distribution family. We show the results as previously described. The source code for the representative images displayed in the research article is available in the [QN](https://github.com/elalilab/Stroke_PDGFR-B_Reactivity/blob/main/Widefield_10x_ROIs_Gfap-Pdgfrb_Covariance.qmd).

## **Supplementary Method 7: Analysis of perivascular and parenchymal PDGFRβ^+^ cells in defined ROIS of the ipsilateral hemisphere.**

| **Component** | **Link** |
| --- | --- |
| Figures | Figure 3B-C, L, Suppl. Figure 3A-G |
| Raw Images | [Widefield_10x_ROIs_CD31-Pdgfrb.zip](https://zenodo.org/api/records/10553084/draft/files/Widefield_10x_ROIs_CD31-Pdgfrb.zip/content) |
| Low-resolution images | [Widefield_10x_ROIs_CD31-Pdgfrb.zip](https://osf.io/csvrx) |
| Image Processing | [Images_Processing/Widefield_10x_ROIs_CD31-Pdgfrb](https://osf.io/9zdc7/) |
| Raw Data | [Raw_Widefield_10x_ROIs_CD31-Pdgfrb_Coloc.csv](https://osf.io/ev5md) |
| Processed data | [Widefield_10x_ROIs_CD31-Pdgfrb_Coloc.csv](https://osf.io/zfcgh) |
| Statistical models | [StatisticalModels_brms/Widefield_10x_ROIs_CD31-Pdgfrb_Coloc](https://osf.io/zvnxa/) |
| Analysis notebook | [Widefield_10x_ROIs_CD31-Pdgfrb_Coloc.qmd](https://github.com/elalilab/Stroke_PDGFR-B_Reactivity/blob/main/Widefield_10x_ROIs_CD31-Pdgfrb_Coloc.qmd) |

We acquired widefield [images](https://osf.io/csvrx) at 10x magnification in the injured cortex (Ctx, cortex), injured striatum (Str, striatum), and the healthy perilesional cortex (Peri, perilesion) of PDGFRβ^tdTomato^ /CD31 immunolabeling in brain sections (see the [protocol](https://osf.io/d2un3/files/9mgyh)) to analyze the proportions of perivascular (vascular-associated) and parenchymal (no vascular-associated) PDGFRβ^+^ cells after ischemic stroke.

We used the [pixel classification](https://osf.io/cvq3a) tool from Ilastik [(Berg et al., 2019)](https://www.zotero.org/google-docs/?LQu4Mu) to enhance the detection of PDGFRβ^tdTomato^ cells. Subsequently, we imported .png segmentation masks into CellProfiler [(Stirling et al., 2021)](https://www.zotero.org/google-docs/?THo9R3) to perform unbiased/automated [cell detection](https://osf.io/6ec89) using the MaskImage and IdentifyPrimaryObjects modules. On the other hand, we detected CD31^+^ cells using the IdentifyPrimaryObjects module and dilated (DilateObjects) the objects with disk = 2 as the structuring element. With this procedure, we aimed to capture the PDGFRβ^+^ cells interacting with CD31^+^ vasculature from a side plane (see graph below). Finally, we related the PDGFRβ^+^ and CD31^+^ objects using the RelateObjects module and exported the [data](https://osf.io/ev5md) as a .csv file. The complete CellProfiler [pipeline](https://osf.io/6ec89) is available in the OSF repository.

|  | PDGFRβ^+^ perivascular pericytes can be detected directly over the vasculature (A) or from a side plane (B). We dilated PDGFRβ^+^ objects to capture cells from both imaging planes. |
| --- | --- |

We analyzed the changes in the proportion of parenchymal PDGFRβ^+^ cells by fitting independent binomial Bayesian models per region ([perilesion](https://osf.io/c84qr), [striatum](https://osf.io/9u7vr), and [cortex](https://osf.io/nb9qx)), as described previously:

$$PDGFR\beta_{parenchymal} | trials(PDGFR\beta_{Total} \sim s(DPI, k=4), family = binomial$$

The modeling strategy, validation, and model comparison are available in [QN](https://github.com/elalilab/Stroke_PDGFR-B_Reactivity/blob/main/Widefield_10x_ROIs_CD31-Pdgfrb_Coloc.qmd). We visualized the results using the conditional_effects function from brms with additional ggplot aesthetics and shared the posterior estimates as .tex tables in the OSF repository. Please note that the [modelbased](https://easystats.github.io/modelbased/) package does not have functions to generate linear derivatives from binomial models.

Complementarily, we [modeled](https://osf.io/82vun) the number (not the proportion) of PDGFRβ^+^ cells with the interaction between DPI and Regions (cortex, striatum, perilesion) as predictors:

$$PDGFR\beta_{parenchymal} \sim DPI * Region, family = hurdle\_lognormal$$

We used a hurdle_lognormal model to account for overdispersion and a high proportion of zeros in perilesional areas. We show these [posterior estimates](https://osf.io/54jek) using conditional_effects and provide the .tex [table](https://osf.io/xy8hr) in the OSF repository. From this model, we also calculated the contrast between the number of PDGFRβ^+^ cells in the cortex and striatum, and [plot](https://osf.io/azf74) them using stat_slab() + stat_pointinterval().

Next, we compared mice with cortico-striatal and striatal-only lesions at 14 and 30 DPI to evaluate the influence of cortical-striatal injuries on the appearance of PDGFRβ^+^ parenchymal cells. For this purpose, we used two modeling strategies (see [QN](https://github.com/elalilab/Stroke_PDGFR-B_Reactivity/blob/main/Widefield_10x_ROIs_CD31-Pdgfrb_Coloc.qmd)). First, given the exploratory data analysis, we fitted a linear [model](https://osf.io/xyat9) with heteroscedasticity (predicting sigma) to estimate the total number of PDGFRβ^+^ cells having the interaction of DPI and type of lesion as predictors:

$$PDGFR\beta_{parenchymal}\sim DPI * Lesion, sigma \sim DPI * Lesion, family = student$$

We show the posterior estimates for the [number of cells](https://osf.io/xthmw) and [sigma](https://osf.io/yqu9z) with their respective posterior [summary](https://osf.io/gksda) as described previously in this section.

Secondly, we [modeled](https://osf.io/9u7vr) the proportion of PDGFRβ^+^ cells with the interaction of DPI and lesion type as predictors:

$$PDGFR\beta_{parenchymal} | trials(PDGFR\beta_{Total} \sim DPI, family = binomial$$

See the posterior [summary](https://osf.io/teu9f) in the OSF repository and the [QN](https://github.com/elalilab/Stroke_PDGFR-B_Reactivity/blob/main/Widefield_10x_ROIs_CD31-Pdgfrb_Coloc.qmd) for further details on the modeling strategy, diagnostics, and source code for data visualization.

Finally, we investigated the effects of 4-OHT-mediated Cre-recombination on the number of parenchymal PDGFRβ^+^ cells. We performed an analogous tamoxifen injection in sham animals and harvested the brains at the same time points as done for MCAO mice. We analyzed the cortex of Sham and MCAO mice using a linear model:

$$PDGFR\beta_{parenchymal} \sim DPI * Condition, family = student$$

We show the [results](https://osf.io/p5asu) and posterior [summary](https://osf.io/tpb4x) as previously described in this section.

## **Supplementary Method 8: Fluorescence-activated cell sorting (FACS) of brain PDGFRβ^+^ cells.**

| **Component** | **Link** |
| --- | --- |
| Figures | Figure 3D-G; Suppl. Figure 4. |
| Raw Images | N/A |
| Low-resolution images | N/A |
| Image Processing | N/A |
| Raw Data | [FACS](https://osf.io/zvnxa/) |
| Processed data | [Data_Processed/Facs_Pdgfrb_Cells-Dead.csv](https://osf.io/7h6mg) |
| Point patterns | N/A |
| Statistical models | [StatisticalModels_brms/Facs_Psgfrb-Dapi](https://osf.io/zvnxa/) |
| Analysis notebook | [FlowCytometry_Pdgfrb_Processing.qmd](https://github.com/elalilab/Stroke_PDGFR-B_Reactivity/blob/main/FlowCytometry_Pdgfrb_Processing.qmd)  [FlowCytometry_Pdgfrb_Analysis.qmd](https://github.com/elalilab/Stroke_PDGFR-B_Reactivity/blob/main/FlowCytometry_Pdgfrb_Analysis.qmd) |

We use the adult brain dissociation kit (Miltenyi Biotec, cat # 130-107-677) to sort PDGFRβ^+^ cells from PDGFRβ^tdTomato^ mice at 14 DPI, as detailed in the [protocol](https://osf.io/mdnzy). To compare the proportion of dead cells between the ipsilateral and contralateral hemispheres, we stained the cells with Fixable Viability Stain 780 (Live/Dead; BD Biosciences, cat #3565388). We analyzed the resulting .fcs files—available in “[Datasets/FACS](https://osf.io/zvnxa/)” component of the OSF repositories—using open-source packages from [Bioconductor](https://www.bioconductor.org/).

We loaded the files using the read.flowset function from the flowCore package [(Hahne et al., 2009)](https://www.zotero.org/google-docs/?bojg7N). Next, we compensated the samples using the compensate function from flowWorkspace and performed quality checks with the flow_auto_qc function from the flowAI package [(Monaco et al., 2016)](https://www.zotero.org/google-docs/?zwtlqp). These procedures ensured the removal of anomalies related to the flow rate, signal acquisition, and dynamic range. We have made the quality check [files](https://osf.io/huf8p) available in the OSF repository. Subsequently, we defined the gating strategy and visualized the clee proportions using functions from the ggcyto package [(Van et al., 2018)](https://www.zotero.org/google-docs/?ZGoAuX). **Supplementary Figure 4** presents plots of the complete sample set. The complete handling and processing pipeline of the raw .fcs files is provided in the [QN](https://github.com/elalilab/Stroke_PDGFR-B_Reactivity/blob/main/FlowCytometry_Pdgfrb_Processing.qmd).

We modeled the number of PDGFRβ^+^ cells per hemisphere using a linear model (single 14 DPI):

$$PDGFR{\beta_{Cells}} \sim Hemisphere, family =student$$

We show the [results](https://osf.io/ac2g4) and posterior [summary](https://osf.io/b7dhn) in the OSF repository.

Second, we modeled the number of PDGFRβ^+^ dead cells distributed by hemisphere at the same DPI. We fitted two models:

Model 1 = $PDGFR{\beta_{Dead}} \sim Hemisphere, family =student$

Model 2 = $PDGFR{\beta_{Dead}} \sim Hemisphere, sigma \sim Hemisphere, family =student$

The posterior predictive checks showed that the model with heteroskedasticity (Model 2) deviates less from the observed data and is less penalized by out-of-sample prediction (see [QN](https://github.com/elalilab/Stroke_PDGFR-B_Reactivity/blob/main/FlowCytometry_Pdgfrb_Analysis.qmd)). We show the [results](https://osf.io/49k2a) and posterior [summary](https://osf.io/dz7tv) for this model in the OSF repository.

## **Supplementary Method 9: PPA and colocalization analysis of Ki67^+^ / PDGFRβ^+^ cells in the ipsilateral hemisphere.**

| **Component** | **Link** |
| --- | --- |
| Figures | Figure 3H-K |
| Raw Images | [Widefield_10x_Ipsilateral_Ki67-Pdgfrb(a).zip](https://zenodo.org/api/records/10553084/draft/files/Widefield_10x_Ipsilateral_Ki67-Pdgfrb(a).zip/content)  [Widefield_10x_Ipsilateral_Ki67-Pdgfrb(b).zip](https://zenodo.org/api/records/10553084/draft/files/Widefield_10x_Ipsilateral_Ki67-Pdgfrb(b).zip/content) |
| Low-resolution images | [Widefield_10x_Ipsilateral_Ki67-Pdgfrb.zip](https://osf.io/52h64) |
| Image Processing | [Images_Processing/Widefield_10x_Ipsilateral_Ki67-Pdgfrb](https://osf.io/9zdc7/) |
| Raw Data | [Data_Raw/Widefield_10x_Ipsilateral_Ki67-Pdgfrb](https://osf.io/zvnxa/) |
| Processed data | [Data_Processed/Widefield_10x_Ipsilateral_Ki97-Pdgfrb](https://osf.io/zvnxa/) |
| Point patterns | [Widefield_10x_Ipsilateral_Ki67-Pdgfrb_PPP.rds](https://osf.io/fxgr6) |
| Statistical models | [StatisticalModels_brms/Widefield_10x_Ipsilateral_Ki67-Pdgfrb](https://osf.io/zvnxa/) |
| Analysis notebook | [Widefield_10x_Ipsilateral_Ki67_PPA.qmd](https://github.com/elalilab/Stroke_PDGFR-B_Reactivity/blob/main/Widefield_10x_Ipsilateral_Ki67_PPA.qmd)  [Widefield_10x_Ipsilateral_Ki67-Pdgfrb_Coloc.qmd](https://github.com/elalilab/Stroke_PDGFR-B_Reactivity/blob/main/Widefield_10x_Ipsilateral_Ki67-Pdgfrb_Coloc.qmd) |

We acquired widefield [images](https://osf.io/52h64) at 10x magnification of PDGFRβ^tdTomato^ / Ki67 imunolabeling in brain sections (see the [protocol](https://osf.io/5jmfk)) in the ipsilateral hemisphere to analyze the proportion and distribution of Ki67^+^/PDGFRβ^+^ cells following injury. First, we performed pixel classification for [Ki67](https://osf.io/dwsv7)^+^ and [PDGFRβ^tdTomato^](https://osf.io/tqs32) using Ilastik to enhance cell detection in CellProfiler. We then imported the segmentation masks into cellProfiler and conducted unbiased, automated object detection using the IdentifyPrimeryObjects module. Next, we filtered Ki67 and PDGFRβ objects to eliminate small particles and abnormally large objects. Finally, we related the objects using the RelateObjects module and exported the data into .csv files. We share the complete [pipeline](https://osf.io/wdzk7) in the OSF repository, which includes the generation of [outlines and crops](https://osf.io/52h64) for visualization, validation, and reuse.

Secondly, we created point patterns using *xy* coordinates for individual [Ki67](https://osf.io/ecq7h)^+^ and [PDGFRβ](https://osf.io/7jmw2)^+^ cells obtained from CellProfiler, as [previously](https://docs.google.com/document/d/1Wg0jb0JgCNJFF7BXj3c8w0YHqb7nHkrXJOaywK8Uco4/edit?tab=t.0#heading=h.5oddfd6j597b) specified. We used the PDGFRβ^tdtomato^ coordinates to define the observation window. We then analyzed the distribution of Ki67^+^ cells conditioned on the spatial intensity of [PDGFRβ](https://osf.io/7jmw2) by calculating the relative distribution (rhohat) and fitting a point process model (mppm) as described earlier. For additional details, please refer to the [QN](https://github.com/elalilab/Stroke_PDGFR-B_Reactivity/blob/main/Widefield_10x_Ipsilateral_Ki67_PPA.qmd). Afterward, we evaluated the PDGFRβ^+^ / Ki67 colocalization in the ipsilateral hemisphere using the [summary](https://osf.io/z6tvk) .csv file from CellProfiler containing cell counts per brain. We estimated the percentage of colocalized cells using a [spline model](https://osf.io/cnrbg):

$$PDGFR\beta\sim s(DPI, k =5), family = lognormal$$

and calculated the corresponding linear [derivatives](https://osf.io/vgyrk), as described previously. We share the [raw model](https://osf.io/jfw3t) summary or [derivatives](https://osf.io/edbkp) as .tex tables in the “[Tables/tex](https://osf.io/e7gwm/)” component of the OSF repository. We visualized the [results](https://osf.io/hqu39) using the conditional_effects with additional ggplot aesthetics.

## **Supplementary Method 10: Analysis of Ki67^+^ / PDGFRβ^tdTomato^ cells in defined ROIs of the ipsilateral hemisphere.**

| **Component** | **Link** |
| --- | --- |
| Figures | Figure 3H-I |
| Raw Images | [Confocal_20x_ROIs_Ki67-Pdgfrb-CD31.zip](http://confocal_20x_rois_ki67-pdgfrb-cd31.zip) |
| Low-resolution images | [Confocal_20x_ROIs_Ki67-Pdgfrb-CD31.zip](https://osf.io/ut7h5) |
| Image Processing | [Images_Processing/Confolcal_20x_ROIs_Ki67-Pdgfrb-CD31](https://osf.io/9zdc7/) |
| Raw Data | [Data_Raw/](https://osf.io/zvnxa/)[Confolcal_20x_ROIs_Ki67-Pdgfrb-CD31](https://osf.io/9zdc7/) |
| Processed data | [Confocal_20x_ROIs_Ki67-Pdgfrb_Coloc.csv](https://osf.io/6a95w) |
| Statistical models | [StatisticalModels_brms/Confocal_20x_ROIsl_Ki67-Pdgfrb_Coloc](https://osf.io/zvnxa/) |
| Analysis notebook | [Confocal_20x_ROIs_Ki67-Pdgfrb_Coloc.qmd](https://github.com/elalilab/Stroke_PDGFR-B_Reactivity/blob/main/Confocal_20x_ROIs_Ki67-Pdgfrb_Coloc.qmd) |

We acquired confocal [images](https://osf.io/ut7h5) at 20 x magnification of PDGFRβ^tdTtomato^ / Ki67 immunolabeling in brain sections (see the [protocol](https://osf.io/5jmfk)) in defined ROIs (healthy cortical peri-lesional region, injured striatum, and injured cortex) to analyze the proportion of vascular (attached to CD31^+^ cells) Ki67^+^ / PDGFRβ^+^ cells. To enhance cell detection in CellProfiler, we used pixel classification for [Ki67](https://osf.io/z6ru4)^+^ and [PDGFRβ^tdTomato^](https://osf.io/2nbzs) using Ilastik. For Ki67, we applied the Smooth and MaskImage modules (utilizing segmentation masks from Ilastik) before proceeding to detect individual cells with IndentifyPrimeryObjects. We then filtered the objects using FilterObjects (*maximum value only* = 2000) to eliminate abnormalities and shrank the remaining objects with ExpandOrShrinkObjects by *10 pixels* to reduce false-positive colocalization caused by cell proximity. We conducted an analogous procedure for PDGFRβ without object shrinkage. Finally, we detected CD31^+^ vasculature by smoothing and merging using SplitOrMergeObjects with a maximum pixel distance of 2, followed by filtering the objects as aforementioned.

Using this analysis pipeline, we quantified (1) the number of perivascular (CD31-attached) PDGFRβ^+^ cells; (2) the number of vascular Ki67^+^ / PDGFRβ^+^ cells; and (3) the total number of Ki67^+^ / PDGFRβ^+^ cells, including both vascular and non-vascular associated cells. The CellProfiler [pipeline](https://osf.io/79wzq), along with a complete set of object [images and outlines](https://osf.io/ut7h5), is available in the OSF repository. To investigate the total number of Ki67^+^ / PDGFRβ^+^ cells, we fitted a Bayesian [model](https://osf.io/hnuca) incorporating the interaction of DPI and brain region (perilesion, striatum, cortex) as predictors:

$$Cells \sim DPI * Region, family = negbinomial$$

We used a negative binomial distribution (family = negbinomial) to model counts and account for overdispersion. In contrast, to [model](https://osf.io/zd5x3) the proportion of vascular associated (CD31-attached) Ki67^+^ / PDGFRβ^+^ cells, we employed the same predictors but used a binomial distribution as previously described:

$$Ki67/PDGFR\beta_{vascular} | trials(Ki67/PDGFR\beta_{total} \sim DPI * Region, family = binomial$$

Additional modeling details are provided in the [QN](https://github.com/elalilab/Stroke_PDGFR-B_Reactivity/blob/main/Confocal_20x_ROIs_Ki67-Pdgfrb_Coloc.qmd). We present the model [results](https://osf.io/mnjhz) using the conditional_effects function and share the [posterior summaries](https://osf.io/9xe52) in the OSF repository.

## **Supplementary Method 11: Morphological analysis of PDGFRβ^+^ cells in defined ROIs of the ipsilateral hemisphere.**

| **Component** | **Link** |
| --- | --- |
| Figures | Figure 4A-C; Suppl. Figure 5A-D |
| Raw Images | [Confocal_40x_ROIs_CD31-Pdgfrb-CD13.zip](https://zenodo.org/api/records/10553084/draft/files/Confocal_40x_ROIs_CD31-Pdgfrb-CD13.zip/content) |
| Low-resolution images | [Confocal_40x_ROIs_CD31-Pdgfrb_CD13.zip](https://osf.io/mfyge)  [Confocal_40x_ROIs_Pdgfrb_Crops.zip](https://osf.io/szpj6)  [Confocal_40x_ROIs_Pdgfrb_Segmented.zip](https://osf.io/h2uxq) |
| Image Processing | [Images_Processing/Confolcal_40x_ROIs_CD31-Pdgfrb-CD13](https://osf.io/9zdc7/) |
| Raw Data | [Data_Raw/](https://osf.io/zvnxa/)[Confolcal_40x_ROIs_Pdgfrb_Morph](https://osf.io/9zdc7/) |
| Processed data | [Confocal_40x_ROIs_Pdgfrb_Morph.csv](https://osf.io/z3hyx) |
| Statistical models | [Confocal_40x_ROIs_Pdgfrb_Morph_Fit1.rds](https://osf.io/d6r38) |
| Analysis notebook | [Confocal_40x_ROIs_Pdgfrb_Morpho_BatchScript.qmd](https://github.com/elalilab/Stroke_PDGFR-B_Reactivity/blob/main/Confocal_40x_ROIs_Pdgfrb_Morpho_BatchScript.qmd)  [Confocal_40x_ROIs_Pdgfrb_Morpho_Analysis.qmd](https://github.com/elalilab/Stroke_PDGFR-B_Reactivity/blob/main/Confocal_40x_ROIs_Pdgfrb_Morpho_Analysis.qmd) |

We acquired confocal z-stack [images](https://osf.io/mfyge) at 40x magnification of PDGFRβ^tdTtomato^ / CD31 / CD13 immunolabeling in brain sections (see the [protocol](https://osf.io/9mgyh)) in defined ROIs (healthy cortical peri-lesional region, injured striatum, and injured cortex) to analyze the morphological features of cells. Using a FIJI [script](https://osf.io/tns7r), we created z-projection images and [manually](https://osf.io/mfyge) cropped them to segment one or more well-differentiated cells (see [resulting cells](https://osf.io/szpj6)). Subsequently, we employed the Python libraries skimage and scipy to enhance, threshold, and generate [masks](https://osf.io/h2uxq) of single cells, as demonstrated in the [QN](https://github.com/elalilab/Stroke_PDGFR-B_Reactivity/blob/main/Confocal_40x_ROIs_Pdgfrb_Morpho_BatchScript.qmd). We measure the morphological features of these masks using the regionprops and regionprops_table functions from skimage.measure.

Afterward, we used the Object Classification module from CellProfiler to perform a user-defined classification into “Amoeboid”, “Perivascular”, “Reticuloparenchymal”, and “Reticulite” cells. We utilized the manually cropped and segmented cells, along with the masks generated by the Python pipeline, as input for classification. We share the resulting [raw data](https://osf.io/zvnxa/) files (.csv per brain) containing the classification results and measured morphological traits, including *Intensity_sd, Intensity_Mean, Defects, Convexity, Area, ConvexHull, BranchLenght, Branches, Diameter, EcuclideanDiameter*.

After loading, cleaning, and processing the raw data files, we created a single [dataset](https://osf.io/z3hyx) containing the relevant grouping variables and morphological traits (see [QN](https://github.com/elalilab/Stroke_PDGFR-B_Reactivity/blob/main/Confocal_40x_ROIs_Pdgfrb_Morpho_Analysis.qmd)). To analyze the data, we performed principal component analysis (PCA) using the FactoMineR [(Lê et al., 2008)](https://www.zotero.org/google-docs/?wa9bDZ) and FactoExtraR packages. Based on the resulting [correlation matrix](https://osf.io/qncv3) and [biplot](https://osf.io/y3nfu), we selected area, convex hull, branch length, and mean intensity as modeling variables. We then fitted a multinomial logistic regression [model](https://osf.io/d6r38) with these morphological features as predictors of cell class (amoeboid, perivascular, reticuloparenchymal, and reticulite):

$$Class \sim Area + ConvexHull + BranchLenght + Intensity\_Mean, family = categorical$$

The modeling strategy and diagnostics are fully described in the [QN](https://github.com/elalilab/Stroke_PDGFR-B_Reactivity/blob/main/Confocal_40x_ROIs_Pdgfrb_Morpho_Analysis.qmd). We present the results as a [spyder plot](https://osf.io/n5ysk) showing means (without displaying uncertainty) and provide individual plots for each morphological attribute using conditional_effects. The [posterior summary](https://osf.io/r6npq) is also available in the OSF repository.

## **Supplementary Method 12: Analysis of covered area and Haralick features (texture) of PDGFRβ^+^ cells in defined ROIs of the ipsilateral hemisphere.**

| **Component** | **Link** |
| --- | --- |
| Figures | Figure 5A; Suppl. Figure 7A-D |
| Raw Images | [Widefield_20x_ROIs_Pdgfrb.zip](https://zenodo.org/api/records/10553084/draft/files/Widefield_20x_ROIs_Pdgfrb.zip/content) |
| Low-resolution images | [Widefield_20x-ROIs_Pdgfrb.zip](https://osf.io/m76pn) |
| Image Processing | [Images_Processing/Widefield_20x_ROIs_Pdgfrb](https://osf.io/9zdc7/) |
| Raw Data | [Raw_Widefield_20x_ROIs_Pdgfrb_Haralick.csv](https://osf.io/xaqbz)  [Data_Raw/Widefield_20x_ROIs_Pdgfrb/CellProfiler](https://osf.io/zvnxa/) |
| Processed data | N/A – Raw data is clean |
| Statistical models | [Widefield_20x_ROIs_Pdgfrb_Area_Fit1.rds](https://osf.io/a8bmh)  [Widefield_20x_ROIs_Pdgfrb_Haralick_Fit1.rds](https://osf.io/ecsjr) |
| Analysis notebook | [Widefield_20x_ROIs_Pdgfrb_Haralick_BatchScript.qmd](https://github.com/elalilab/Stroke_PDGFR-B_Reactivity/blob/main/Widefield_20x_ROIs_Pdgfrb_Haralick_BatchScript.qmd)  [Widefield_20x_ROIs_Pdgfrb_Haralick.qmd](https://github.com/elalilab/Stroke_PDGFR-B_Reactivity/blob/main/Widefield_20x_ROIs_Pdgfrb_Haralick.qmd) |

We acquired widefield z-stack [images](https://osf.io/m76pn) at 20x magnification of PDGFRβ^tdTomato^ / PDGFRɑ^+^ immunolabeling in brain sections (see the [protocol](https://osf.io/5hm42)) in defined ROIs (healthy cortical perilesion, injured striatum, and injured cortex) to analyze the spatial and topological arrangement of cells. For this analysis, we focused exclusively on PDGFRβ⁺ cells. Using [FIJI](https://osf.io/y3qpk), we created two-dimensional z-projections and analyzed the resulting images with CellProfiler. We identified single cells using the IndentifyPrimaryObjects module and measured each object’s occupied area with the MeasureImageAreaOccupied module. We analyzed the cell area data using a linear [model](https://osf.io/a8bmh) with DPI and Region (cortex, striatum, perilesion) as predictors (see [QN](https://github.com/elalilab/Stroke_PDGFR-B_Reactivity/blob/main/Widefield_20x_ROIs_Pdgfrb_Haralick.qmd)):

$$Area \sim DPI\_cont * Region, family = student$$

We present the [results](https://osf.io/yqs75) using conditional_effects function with additional ggplot aesthetics and have made the [posterior summary](https://osf.io/azujf) available in the OSF repository. Subsequently, we measured Haralick features (see table below) [(Löfstedt et al., 2019)](https://www.zotero.org/google-docs/?FUyLnr) using the mahotas Python package [(Coelho, 2013)](https://www.zotero.org/google-docs/?OWP2jr) as detailed in the [QN](https://github.com/elalilab/Stroke_PDGFR-B_Reactivity/blob/main/Widefield_20x_ROIs_Pdgfrb_Haralick_BatchScript.qmd).

| **Haralick features** | **Description** |
| --- | --- |
| Angular Second Moment (ASM) / Energy | Measures the uniformity of an image. A higher value indicates that the image has more uniform textures or constant regions. |
| Contrast | Represents the difference between the highest and the lowest intensity value in the co-occurrence matrix. It measures the amount of local variations present in an image. |
| Correlation | Measures the joint probability occurrence of the specified pixel pairs. It provides information about the linear dependency of gray levels in the neighboring pixels. |
| Sum of Squares / Variance | It provides a measure of the squared differences from the mean intensity value. |
| Inverse Difference Moment (IDM) / Homogeneity | Measures the local homogeneity of an image. The values are high when the local textures are consistent or homogeneous. |
| Sum Average | Represents the average intensity value of the co-occurrence matrix. |
| Sum Variance | Measures the variance of the sum of the intensity values from the average value in the co-occurrence matrix. |
| Sum Entropy | Represents the randomness or complexity in the sum of the intensity values of the co-occurrence matrix. |
| Entropy | Provides a measure of the randomness or complexity of the image. Higher values indicate more complex textures. |
| Difference Variance | Represents the variance in the differences between the intensity values of pairs of pixels. |
| Difference Entropy | Measures the randomness or complexity in the differences between the intensity values of pairs of pixels. |
| Informational Correlation 1 (Info Corr 1) | Represents the correlation between the occurrence of the specified pixel pairs and their average intensity values. |
| Informational Correlation 2 (Info Corr 2) | Provides another measure of the correlation between the occurrence of the specified pixel pairs and their average intensity values. It’s typically more sensitive to changes than Info Corr 1. |

We analyzed the Haralick features using PCA and presented the results as previously described. Based on the PCA, we selected entropy, contrast, and inverse difference moment (IDM) as the most relevant features (see [QN](https://github.com/elalilab/Stroke_PDGFR-B_Reactivity/blob/main/Widefield_20x_ROIs_Pdgfrb_Haralick.qmd)). We performed statistical modeling by fitting a multinomial logistic regression [model](https://osf.io/ecsjr) in brms to predict the brain region (perilesion, striatum or cortex) based on the selected Haralick features:

$$Region \sim Entropy + Contrast +IDM, family = categorical$$

Model diagnostics are available in the [QN](https://github.com/elalilab/Stroke_PDGFR-B_Reactivity/blob/main/Widefield_20x_ROIs_Pdgfrb_Haralick.qmd). We visualize the results for each feature using the conditional_effects function and have made the [posterior summary](https://osf.io/7m4ju) in the OSF repository.

## **Supplementary Method 13: Topological data analysis (TDA) of PDGFRβ^+^ cells in defined ROIs of the ipsilateral hemisphere.**

| **Component** | **Link** |
| --- | --- |
| Figures | Figure 5B-E; Suppl. Figure 7E-F |
| Raw Images | [Widefield_20x_ROIs_Pdgfrb.zip](https://zenodo.org/api/records/10553084/draft/files/Widefield_20x_ROIs_Pdgfrb.zip/content) |
| Low-resolution images | [Widefield_20x-ROIs_Pdgfrb.zip](https://osf.io/m76pn) |
| Image Processing | [Images_Processing/Widefield_20x_ROIs_Pdgfrb](https://osf.io/9zdc7/) |
| Raw Data | [Data_Raw/Widefield_20x_ROIs_Pdgfrb/CellProfiler](https://osf.io/zvnxa/) |
| Processed data | [Data_Processed/Widefield_20x_ROIs_Pdgfrb/](https://osf.io/zvnxa/) |
| Statistical models | N/A |
| Analysis notebook | [Widefield_20x_ROIs_Pdgfrb_TDA.ipynb](https://github.com/elalilab/Stroke_PDGFR-B_Reactivity/blob/main/Widefield_20x_ROIs_Pdgfrb_TDA.ipynb) |

We performed TDA using the same images and datasets from the previous section. We obtained single *xy* [cell coordinates](https://osf.io/vzhxe) from CellProfiler to serve as input for TDA. Utilizing the GUDHI Python library [(The GUDHI Project, 2023)](https://www.zotero.org/google-docs/?RzLmBe), we calculated Vietoris-rips complex [(Chambers et al., 2010)](https://www.zotero.org/google-docs/?bdowtF) with a maximum dimension of 2 and a radius of 100 (see [JN](https://github.com/elalilab/Stroke_PDGFR-B_Reactivity/blob/main/Widefield_20x_ROIs_Pdgfrb_TDA.ipynb)). We have made available a full set of [static](https://osf.io/f59bt) and [animated](https://osf.io/49aqu) (see [example](https://osf.io/zteg3)) Vietoris-rips complex visualizations for reuse in research or educational settings.

To calculate persistent homology [(Aktas et al., 2019; Otter et al., 2017)](https://www.zotero.org/google-docs/?ukph3u), we generated [point clouds](https://osf.io/rykgn) and saved them as .npy Numpy arrays. We used the ripser Python library [(Tralie et al., 2018)](https://www.zotero.org/google-docs/?RqSge9) to compute persistent homology for dimension-0 (0D, connected components) and dimension-1 (1D, loops). The complete set of [persistent diagrams](https://osf.io/fyasg) is shared as .npy files for research purposes and as .png for [visualization](https://osf.io/exf7s). Subsequently, we calculated Betti curves to analyze the evolution of the topological features revealed by persistent homology. For connected components (0D homology), we calculated normalized (to 1) Betti curves (steps = 300, max filtration = 300) to eliminate the bias by the number of components (cells). For loops (1D homology), we employed 100 steps and a maximum filtration value of 700 without normalization. We plotted the Betti curves per dimension and region as line plots using matplotlib [(Hunter, 2007)](https://www.zotero.org/google-docs/?Zrnmb4). To quantify the differences between DPIs, we calculated Bottleneck and Wasserstein distances [(Hajij et al., 2018)](https://www.zotero.org/google-docs/?uECPUk) using the bottleneck_distance and wasserstein_distance functions from the gudhi library, respectively. We obtained median estimates with their respective uncertainty by bootstrapping [(Chernik, 2007)](https://www.zotero.org/google-docs/?gzUk3L) the samples with 1000 replications using the choices function from the random library. We present the results for Bottleneck and Wasserstein distances as DPI contrast heatmaps, displaying median values and 95% confidence intervals. For additional details on Vietoris-Rips complexes, persistent homology, Betti curves, and Bottleneck/Wasserstein calculations, please refer to the [JN](https://github.com/elalilab/Stroke_PDGFR-B_Reactivity/blob/main/Widefield_20x_ROIs_Pdgfrb_TDA.ipynb).

## **Supplementary Method 14: Spatial and labeling intensity analysis of KLF4 in the ipsilateral hemisphere.**

| **Component** | **Link** |
| --- | --- |
| Figures | Figure 6A-C; Suppl. Figure 8A-C |
| Raw Images | [Widefield_10x_Ipsilateral_KLF4.zip](https://zenodo.org/api/records/10553084/draft/files/Widefield_10x_Ipsilateral_KLF4.zip/content)  [Widefield_10x_Ipsilateral_KLF4.z01](https://zenodo.org/api/records/10553084/draft/files/Widefield_10x_Ipsilateral_KLF4.z01/content)  [Widefield_10x_Ipsilateral_KLF4.z02](https://zenodo.org/api/records/10553084/draft/files/Widefield_10x_Ipsilateral_KLF4.z02/content) |
| Low-resolution images | [Widefield_10x_Ipsilateral_Klf4.zip](https://osf.io/86wdv) |
| Image Processing | [Images_Processing/Widefield_10x_Ipsilateral_Klf4](https://osf.io/9zdc7/) |
| Raw Data | [Data_Raw/Widefield_10x_Ipsilateral_Klf4](https://osf.io/zvnxa/) |
| Processed data | [Data_Processed/Widefield_10x_Ipsilateral_Klf4](https://osf.io/zvnxa/) |
| Point patterns | [Widefield_10x_Ipsilateral_Klf4_PPP.rds](https://osf.io/jdk59) |
| Statistical models | [StatisticalModels_brms/Widefield_10x_Ipsilateral_Klf4](https://osf.io/zvnxa/) |
| Analysis notebook | [Widefield_10x_Ipsilateral_Klf4_Exp.qmd](https://github.com/elalilab/Stroke_PDGFR-B_Reactivity/blob/main/Widefield_10x_Ipsilateral_Klf4_Exp.qmd) |

We acquired widefield [images](https://osf.io/52h64) at 10 x magnification of PDGFRβ^tdTomato^ / KLF4 immunolabeling in brain sections (see the [protocol](https://osf.io/qu7hp)) in the ipsilateral hemisphere to analyze the spatial distribution and signal intensity of KLF4 following ischemia. To improve cell detection in [CellProfiler](https://osf.io/e5y86), we performed [pixel classification](https://osf.io/7xj23) for KLF4 using Ilastik. We detected KLF4^+^ cells by using the segmentation masks as input for the IdentifyPrimeryObjects module in CellProfiler. Additionally, we detected DAPI^+^ cells to define the observation window for point patterns. The CellProfiler pipeline includes the generation of [cropped](https://osf.io/86wdv) images representing the detection of single cells. We processed the raw files from CellProfiler to build [point patterns](https://osf.io/jdk59) (ppp), calculate the spatial intensity ($intensity), derive density kernels (density), and fit multiple point process models (mppm). Please refer to the [QN](https://github.com/elalilab/Stroke_PDGFR-B_Reactivity/blob/main/Widefield_10x_Ipsilateral_Klf4_Exp.qmd) for further details and the source code. To evaluate the distribution of KLF4 following injury, we fitted a mixed-effects model using spatstat, with the x-axis (representing distance from the ischemic area in the ipsilateral hemisphere) as a predictor varying by DPI:

$$KLF4 \sim x, random = \sim x | DPI, family = Poisson$$

Please note that the model summary in the [QN](https://github.com/elalilab/Stroke_PDGFR-B_Reactivity/blob/main/Widefield_10x_Ipsilateral_Klf4_Exp.qmd) is on the log scale.

To account for variance across different DPIs, we extracted the spatial intensity of KLF4⁺ cells from the point patterns and fitted a linear model with heteroskedasticity using brms. Specifically, we modeled intensity as a function of DPI without an intercept and allowed the residual variance (σ) to vary with DPI:

$$Intensity \sim0 + DPI, sigma \sim0 + DPI, family=student$$

Model diagnostics and source code are available in the [QN](https://github.com/elalilab/Stroke_PDGFR-B_Reactivity/blob/main/Widefield_10x_Ipsilateral_Klf4_Exp.qmd). We present the results as full posterior distributions per DPI for both the parameter [estimates](https://osf.io/ts5fh) (add_predicted_draws + stat_haleye from tidybayes) and the residual standard deviation ([sigma](https://osf.io/gnx7p)) (conditional_effects). We calculated the [contrast](https://osf.io/mygbz) between time points using the emmeans package [(Lenth, 2024)](https://www.zotero.org/google-docs/?Ds4Rt3) as detailed in the [QN](https://github.com/elalilab/Stroke_PDGFR-B_Reactivity/blob/main/Widefield_10x_Ipsilateral_Klf4_Exp.qmd). The [posterior summary](https://osf.io/c9w7p) is shared as a .tex table in the OSF repository. We performed an analogous analysis for point patterns and estimated KLF4 mean intensity for brains derived from PDGFRβ^KLF4-KO^ mice. Subsequently, we analyzed the labeling intensity of KLF4 on a per-cell basis, as obtained from the CellProfiler pipeline. Due to computational limitations with the full dataset of 278,340 observations, we randomly subsampled 10% of the data using sample_frac(0.1) and fitted a multilevel model with a hurdle_lognormal() distribution to handle zero-inflated data and capture continuous observations:

$$Intensity \sim0 + DPI + (1 | MouseID), family =hurdle\_lognormal()$$

We included MouseID as a random intercept to account for the hierarchical structure of the data and to obtain more precise estimates by integrating variance within mice. Modeling details and diagnostics are available in the [QN](https://github.com/elalilab/Stroke_PDGFR-B_Reactivity/blob/main/Widefield_10x_Ipsilateral_Klf4_Exp.qmd). We present the [results](https://osf.io/hjx6s) using conditional effects, rendering point estimates with 95% CI, and [contrasts](https://osf.io/2fxvq) between DPIs using stat_pointinterval.

Additionally, we fitted a second multilevel [model](https://osf.io/q67h4) to evaluate the influence of the x-coordinates within the ipsilateral hemisphere on KLF4 labeling intensity. We centered the x-coordinates to mitigate bias from brain shrinkage and to facilitate parameter space exploration. Employing a similar modeling strategy with random intercepts for MouseID, we specified:

$$Intensity \sim0 + DPI * x.coor+ (1 | MouseID), family =hurdle\_lognormal()$$

We visualized the results using conditional_effects, rendering colored line plots per DPI with 95% CI. Additionally, we generated [scatter plots](https://osf.io/9ngdq) of cell positions with a *Viridis* color scale to depict KLF4 immunolabeling intensity per cell.

## **Supplementary Method 15: Analysis of PDGFRβ^+^ / KLF4^+^ cells in defined ROIs of the ipsilateral hemisphere.**

| **Component** | **Link** |
| --- | --- |
| Figures | Figure 6D-E; Suppl. Figure 8D |
| Raw Images | [Confocal_20x_ROIs_Klf4-Pdgfrb-CD31(a).zip](https://zenodo.org/api/records/10553084/draft/files/Confocal_20x_ROIs_Klf4-Pdgfrb-CD31(a).zip/content)  [Confocal_20x_ROIs_Klf4-Pdgfrb-CD31(b).zip](https://zenodo.org/api/records/10553084/draft/files/Confocal_20x_ROIs_Klf4-Pdgfrb-CD31(b).zip/content) |
| Low-resolution images | [Confocal_20x_ROIs_Klf4-Pdgfrb_Overlay.zip](https://osf.io/69m32) |
| Image Processing | [Images_Processing/Confocal_20x_ROIs_Klf4-Pdgfrb-CD31](https://osf.io/9zdc7/) |
| Raw Data | [Data_Raw/](https://osf.io/zvnxa/)[Confocal_20x_ROIs_Klf4-Pdgfrb-CD31](https://osf.io/9zdc7/) |
| Processed data | [Confocal_20x_ROIs_Klf4-Pdgfrb_Coloc.csv](https://osf.io/425ed) |
| Statistical models | [StatisticalModels_brms/](https://osf.io/zvnxa/)[Confocal_20x_ROIs_Klf4-Pdgfrb-CD31](https://osf.io/9zdc7/) |
| Analysis notebook | [Confocal_20x_ROIs_Klf4-Pdgfrb_Coloc.qmd](https://github.com/elalilab/Stroke_PDGFR-B_Reactivity/blob/main/Confocal_20x_ROIs_Klf4-Pdgfrb_Coloc.qmd) |

We acquired confocal [images](https://osf.io/69m32) at 20x magnification of PDGFRβ^tdTomato^ / KLF4 / CD31 immunolabeling in brain sections (see the [protocol](https://osf.io/qu7hp)) in defined ROIs (contralateral hemisphere, healthy perilesion, and injured cortex) to analyze PDGFRβ^tdTtomato^ and KLF4 co-localization. This dataset differs from others in that we included the contralateral hemisphere instead of the injured striatum. We used the contralateral hemisphere as a control (ground truth) for colocalization error, given that in healthy tissue, KLF4 is prominently expressed in the vasculature by CD31⁺ brain endothelial cells but not in PDGFRβ⁺ perivascular cells.

To enhance cell detection and co-localization analysis in [CellProfiler](https://osf.io/meqa7), we performed [pixel classification](https://osf.io/qyxur) for KLF4 using Ilastik. For analyzing colocalization, we employed a pipeline that included filling (FillObjects), filtration (FilterObjects), and erosion (ErodeObjects) of PDGFRβ^+^ and KLF4^+^ cells. This approach aimed to reduce the rate of false positives in PDGFRβ / KLF4 co-localization due to the proximity of these two markers in the perivascular space. We calculated co-localization by cell counts using the RelateObjects module. Note that CD31 was included in this pipeline solely to generate reference visualizations, as shown in **Figure 6D**. The pipeline also generated images with [overlaid contours](https://osf.io/69m32) (found in the “ObjectsOverlay” folder) for visualization and illustration purposes **(see Figure 6D)**.

We processed the CellProfiler [output](https://osf.io/tyejk) to obtain a clean [dataframe](https://osf.io/425ed) containing the counts of PDGFRβ^+^, KLF4^+,^ and co-localized PDGFRβ^+^ / KLF4^+^ cells. Our statistical inference was based on four different binomial models, as previously described. First, we fitted a linear [model](https://osf.io/c7f48) (Mdl1) to explore the distribution of PDGFRβ^+^/ KLF4^+^ co-localized cells across distinct DPIs:

$PDGFR\beta-KLF4 | trials(Klf4) \sim DPI, family = binomial$

Secondly, using the same approach, we [modeled](https://osf.io/pv42k) (Mdl2) this cell distribution by brain regions— injured cortex (Ctx), healthy cortical perilesion (Peri), and contralateral hemisphere (Ctr)s—. Next, excluding data from day 0 (sham animals), we fitted a third [model](https://osf.io/qwgs7) (Mdl3) to evaluate PDGFRβ^+^ / KLF4^+^ co-localization, conditioning on the interaction effect between DPI and Regions. We excluded sham animals because this group does not have differentiated injured cortex or perilesion areas. The model takes the following notation:

$$PDGFR\beta-KLF4 | trials(Klf4) \sim DPI * Region, family = binomial$$

Finally, we applied the same strategy to fit a fourth [model](https://osf.io/xnzjm) (Mdl4) estimating the proportion of PDGFRβ^+^ / KLF4^+^ co-localization relative to the number of PDGFRβ^+^ cells. For additional details on the modeling strategy and diagnostics, please refer to the [QN](https://github.com/elalilab/Stroke_PDGFR-B_Reactivity/blob/main/Confocal_20x_ROIs_Klf4-Pdgfrb_Coloc.qmd). Note that the summaries of binomial models (Bernoulli trials) are presented on the logit scale. We visualized the model results using conditional_effects, displaying point estimates with 95% Cis and, when applicable, color codes for different brain regions. Model summaries are also available in the `Tables` component of the OSF repository.

## **Supplementary Method 16: Analysis of KLF4 expression in PDGFRβ^KLF4-KO^ mice.**

| **Component** | **Link** |
| --- | --- |
| Figures | Figure 7A-E |
| Raw Images | [Widefield_10x_Ipsilateral_KO_Klf4-Pdgfrb-CD31(a).zip](https://zenodo.org/api/records/10553084/draft/files/Widefield_10x_Ipsilateral_KO_Klf4-Pdgfrb-CD31(a).zip/content)  [Widefield_10x_Ipsilateral_KO_Klf4-Pdgfrb-CD31(b).zip](https://zenodo.org/api/records/10553084/draft/files/Widefield_10x_Ipsilateral_KO_Klf4-Pdgfrb-CD31(b).zip/content) |
| Low-resolution images | [Widefield_10x_Ipsilateral_KO_Klf4-Pdgfrb-CD31.zip](https://osf.io/jrghm) |
| Image Processing | [Images_ProcessingWidefield_10x_Ipsilateral_KO_Klf4-Pdgfrb-CD31](https://osf.io/9zdc7/) |
| Raw Data | [Data_Raw/Widefield_10x_Ipsilateral_KO_Klf4](https://osf.io/zvnxa/) |
| Processed data | [Data_Processed/Widefield_10x_Ipsilateral_KO_Klf4](https://osf.io/zvnxa/) |
| Statistical models | [StatisticalModels_brms/Widefield_10x_Ipsilateral_KO_Klf4_Exp](https://osf.io/zvnxa/) |
| Analysis notebook | [Widefield_10x_Ipsilateral_KO_Klf4_Exp.qmd](https://github.com/elalilab/Stroke_PDGFR-B_Reactivity/blob/main/Widefield_10x_Ipsilateral_KO_Klf4_Exp.qmd) |

We acquired widefield images at 10x magnification of KLF4^+^ / PDGFRβ^+^ / CD31^+^ immunolabeling in brain sections (see the [protocol](https://osf.io/qu7hp)) from the ipsilateral hemisphere of PDGFRβ^KLF4-KO^ mice and corresponding controls. We included four sham-operated animals as a reference group for exploratory data visualization and to estimate error ratios. We performed [pixel classification](https://osf.io/34zxy) for KLF4 in Ilastik to eliminate the background and improve cell detection in [CellProfiler](https://osf.io/c8jga). The generated binary masks were used as input for the IndentifyPrimeryObjects module in CellProfiler. Additionally, we detected DAPI^+^ cells to establish the observation window for point patterns. We exported the data sheets and [cropped](https://osf.io/jrghm) images (’KLF4_Crops’ folder) illustrating the identified cells.

Our initial aim was to analyze KLF4 specifically in PDGFRβ^+^ cells, segregated from CD31^+^ vasculature. However, antibody staining made this analysis impracticable and susceptible to multiple systematic errors. In contrast to PDGFRβ^tdTomato^ cells, antibody labeling does not fill the cell nuclei expected to colocalize with KLF4. Subsequently, we processed (see [QN](https://github.com/elalilab/Stroke_PDGFR-B_Reactivity/blob/main/Widefield_10x_Ipsilateral_KO_Klf4_Exp.qmd)) the CellProfiler [output](https://osf.io/e9pgs) to extract the coordinates of individual cells and constructed [point patterns](https://osf.io/jdk59) using spatstat. We generated and plotted density kernels (sigma = 0.02) and performed mppm to analyze the relative distribution of KLF4 along the x-coordinates:

$KLF4 \sim x, random =\sim x| Genotype, family = Poisson$

We extracted the mean intensity from the point pattern hyperframe and modeled this variable using brms. We fitted an intercept-only [model](https://osf.io/5pwq9) (Intensity ~ 1) using the data from sham animals to estimate a baseline value for KLF4 expression. Then, we fitted a linear [model](https://osf.io/24ms7) with heteroskedasticity with ‘genotype’ as predictor:

$$Intensity \sim Genotype, sigma \sim Genotype, family = student$$

Model diagnostics are available in the [QN](https://github.com/elalilab/Stroke_PDGFR-B_Reactivity/blob/main/Widefield_10x_Ipsilateral_KO_Klf4_Exp.qmd). We calculated the contrast between PDGFRβ^KLF4-KO^ and controls using the emmeans package and presented the results as half-eye densities and point intervals. The model summary is also [available](https://osf.io/qmpkn) in the “Tables” component of the OSF repository.

## **Supplementary Method 17: Analysis of brain shrinkage and PDGFRβ / GFAP expression in PDGFRβ^KLF4-KO^ mice.**

| **Component** | **Link** |
| --- | --- |
| Figures | Figure 7F-H; Suppl. Figure 9A-C |
| Raw Images | [Widefield_5x_Ipsilateral_KO_Pdgfrb-NeuN-Gfap.zip](https://zenodo.org/api/records/10553084/draft/files/Widefield_5x_Ipsilateral_KO_Pdgfrb-NeuN-Gfap.zip/content)  [Widefield_5x_Whole_KO_Pdgfrb-NeuN-Gfap.zip](https://zenodo.org/uploads/10553084) |
| Low-resolution images | [Widefield_5x_Ipsilateral_KO_Pdgfrb-NeuN-Gfap.zip](https://osf.io/r294m) |
| Image Processing | [Images_Processing/](https://osf.io/9zdc7/)[Widefield_5x_Ipsilateral_KO_Pdgfrb-NeuN-Gfap](https://zenodo.org/api/records/10553084/draft/files/Widefield_5x_Ipsilateral_KO_Pdgfrb-NeuN-Gfap.zip/content) |
| Raw Data | [Data_Raw/Widefield_5x_Ipsilateral_KO_Pdgfrb-NeuN-Gfap](https://osf.io/zvnxa/) |
| Processed data | [Data_Processed/Widefield_5x_Ipsilateral_KO_Structure.csv](https://osf.io/4dpze) |
| Statistical models | [StatisticalModels_brms/Widefield_5x_Ipsilateral_KO_Pdgfrb-NeuN-Gfap_Structure](https://osf.io/zvnxa/) |
| Analysis notebook | [Widefield_5x_Ipsilateral_KO_Structure.qmd](https://github.com/elalilab/Stroke_PDGFR-B_Reactivity/blob/main/Widefield_5x_Ipsilateral_KO_Structure.qmd) |

We acquired widefield images at 5x magnification of PDGFRβ^+^ / NeuN+ / GFAP^+^ immunolabeling in brain sections from PDGFRβ^KLF4-KO^ mice and corresponding controls. We included four sham-operated animals as a reference group for exploratory data visualization and to estimate error ratios. To calculate the amount of hemispheric atrophy (shrinkage), we measured the area of the ischemic and contralateral hemispheres using the standard *measure* function in FIJI.

For the analysis of brain shrinkage, we first estimated the intrinsic area ratio (ipsilateral/contralateral) in healthy brains by fitting a statistical [model](https://osf.io/mcwpy) with no predictors (Shrinkage ~ 1, family = student). Then, we fitted a linear model to analyze the distribution of brain shrinkage by genotype:

$$Shrinkage \sim Genotype, family = student$$

We share the [model summary](https://osf.io/2txav) as .tex table in the “[Tables/tex](https://osf.io/e7gwm/)” component of the OSF repository. For the visualization of the results, we obtained posterior draws using the spread_draws function and generated a [density plot](https://osf.io/mh9xq) using stat_halfeye with the ROPE limits in cyan.

Next, we performed pixel classification for [PDGFRβ](https://osf.io/nys5f) and [GFAP](https://osf.io/u2akw) in Ilastik to analyze the integrated density of these markers in [CellProfiler](https://osf.io/cxnbd). We exported binary masks from Ilastik into CellProfiler with the MaskImage module to measure the intensity of PDGFRβ and GFAP solely in the trained regions using the MeasureImageIntensity module. We exported the results into a .cvs file for statistical modeling in R. We fitted analogous linear models for PDGFRβ and GFAP to estimate the marker intensity conditioning on genotype:

$$Intensity \sim Genotype, family = student$$

We present the results using the spread_draws function and [density plots](https://osf.io/mh9xq) with stat_halfeye*.* In both cases, the ROPE is shown as cyan*.* We share the model summaries as .tex tables in the “[Tables/tex](https://osf.io/e7gwm/)” component of the OSF repository.

## **Supplementary Method 18: Analysis of PDGFRβ mRNA transcript spatial expression in the brain of PDGFRβ^KLF4-KO^ mice.**

| **Component** | **Link** |
| --- | --- |
| Figures | Suppl. Figure 9D-F |
| Raw Images | [Widefield_10x_ROIs_KO_Fish_Pdgfrb.zip](https://zenodo.org/records/10553084/files/Widefield_10x_ROIs_KO_Fish_Pdgfrb.zip?download=1) |
| Low-resolution images | [Widefield_10x_ROIs_KO_Fish_Pdgfrb.zip](https://osf.io/mb257/files/osfstorage/6705ad2bd235e5575d9fd437) |
| Image Processing | [Images_Processing/Widefield_10x_ROIs_KO_Fish_Pdgfrb](https://osf.io/9zdc7/files) |
| Raw Data | [Data_Raw/](https://osf.io/zvnxa/)[Widefield_10x_ROIs_KO_Fish_Pdgfrb](https://osf.io/mb257/files/osfstorage/6705ad2bd235e5575d9fd437) |
| Processed data | [Data_Processed/](https://osf.io/4dpze)[Widefield_10x_ROIs_KO_Fish_Pdgfrb](https://osf.io/mb257/files/osfstorage/6705ad2bd235e5575d9fd437) |
| Statistical models | [StatisticalModels_brms/](https://osf.io/zvnxa/)[Widefield_10x_ROIs_KO_Fish_Pdgfrb](https://osf.io/mb257/files/osfstorage/6705ad2bd235e5575d9fd437) |
| Analysis notebook | [Widefield_10x_ROIs_KO_Fish_Pdgfrb.qmd](https://github.com/elalilab/Stroke_PDGFR-B_Reactivity/blob/main/Widefield_10x_Ipsilateral_KO_Fish_Pdgfrb.qmd)  [Widefield_10x_ROIs_KO_Pdgfrb_TDA.ipynb](https://github.com/elalilab/Stroke_PDGFR-B_Reactivity/blob/main/Widefield_10x_ROIs_KO_Pdgfrb_TDA.ipynb) |

We explored PDGFRβ at the mRNA level using fluorescence in situ hybridization (FISH) in PDGFRβ^KLF4-KO^ mice brains (see the [protocol](https://osf.io/n39cu)). We acquired widefield images at 10x magnification in cortical ROIs. We used the IdentifyPrimeryObjects module for CellProfiler to identify labelled cells and measured the morphological properties and intensities of the objects using the MeasureObjectSizeShape and MeasureObjectIntensity modules, respectively. Detected cells are available in the low-resolution component of the OSF.

To analyze the number of PDGFRβ^+^ cells at 14 DPI, we fitted a multilevel negative binomial [model](https://osf.io/zvnxa/files/osfstorage/6705af25e5e223a70fa20c9f) to account for population-level effects of genotype and group-level variability due to individual differences among mice:

$$Counts \sim Genotype + (1 | MouseID), family = negbinomial$$

We visualized the posterior distribution for population and group-level effects using stat_halfeye combined with geom_jitter to show actual measurements.

Next, we evaluated PDGFRβ expression intensity at the cellular level by analyzing the object-level [output](http://data_raw/Widefield_10x_ROIs_KO_Fish_Pdgfrb) from CellProfiler containing area and intensity data. We fitted a mixed-effects [model](https://osf.io/zvnxa/files/osfstorage/6705af26a48948054f5ccf8a) allowing for the interaction between cell area and genotype, using scaled predictors to improve model fit:

$$Intensity \sim Area * Genotype + (1 | MouseID), family = student$$

We visualized the results using conditional effects plots to display the relationship between nuclei area and PDGFRβ intensity by genotype. Both model diagnostics are available in the [QN](https://github.com/elalilab/Stroke_PDGFR-B_Reactivity/blob/main/Widefield_10x_Ipsilateral_KO_Fish_Pdgfrb.qmd). We share the [model summaries](https://osf.io/wz8qa) as .tex tables in the “Tables/tex” component of the OSF repository.

Finally, we performed TDA to evaluate the spatial organization of PDGFRβ^+^ nuclei (see [QN](http://widefield_10x_rois_ko_pdgfrb_tda.ipynb)). First, we generated point clouds from the xy-coordinates of individual nuclei (CellProfiler output) for each animal and section. Then, we computed Vietoris-Rips complexes and persistence diagrams using the GUDHI and Ripser libraries in Python. We analyzed 0-dimensional (connected components) and 1-dimensional (loops/holes) homology features by calculating Betti curves. Finally, we aggregated these curves by genotype (PDGFRβ^Flox^ vs. PDGFRβ^KLF4-KO^) to compare the topological features of cell distribution. We then visualized the results as mean Betti curves with standard deviation for both dimensions **(see Suppl. Figure 9F)**.

**Supplementary Method 19: Analysis of PDGFRβ mRNA transcript spatial expression in the brain of PDGFRβ^KLF4-KO^, KLF4^Flox^, and PDGFRβ^Cre^ mice.**

| **Component** | **Link** |
| --- | --- |
| Figures | Suppl. Figure 9G-H |
| Raw Images | [Widefield_10x_ROIs_Flox-Cre_Fish_Pdgfrb.zip](https://osf.io/mb257/files/osfstorage/6938b1dc99d2f7c14649fe9f) |
| Low-resolution images | [Widefield_10x_ROIs_Flox-Cre_Fish_Pdgfrb.zip](https://osf.io/mb257/files/osfstorage/6938b1dc99d2f7c14649fe9f) |
| Image Processing | [Images_Processing/](https://osf.io/9zdc7/files)[Widefield_10x_ROIs_Flox-Cre_Fish_Pdgfrb](https://osf.io/mb257/files/osfstorage/6938b1dc99d2f7c14649fe9f) |
| Raw Data | [Data_Raw/](https://osf.io/zvnxa/)[Widefield_10x_ROIs_Flox-Cre_Fish_Pdgfrb](https://osf.io/mb257/files/osfstorage/6938b1dc99d2f7c14649fe9f) |
| Processed data | [Data_Processed/](https://osf.io/4dpze)[Widefield_10x_ROIs_Flox-Cre_Fish_Pdgfrb](https://osf.io/mb257/files/osfstorage/6938b1dc99d2f7c14649fe9f) |
| Statistical models | [StatisticalModels_brms/](https://osf.io/zvnxa/)[Widefield_10x_ROIs_Flox-Cre_Fish_Pdgfrb](https://osf.io/mb257/files/osfstorage/6938b1dc99d2f7c14649fe9f) |
| Analysis notebook | [Widefield_10x_ROIs_Flox-Cre_Fish_Pdgfrb.qmd](https://github.com/elalilab/Stroke_PDGFR-B_Reactivity/blob/main/Widefield_10x_ROIs_Flox-Cre_Fish_Pdgfrb.qmd) |

We analysed PDGFRβ mRNA transcript spatial expression in the brain of PDGFRβ^KLF4-KO^, KLF4^Flox^, and PDGFRβ^Cre^ mice as described in Supplementary Method 18 (See [QN](https://github.com/elalilab/Stroke_PDGFR-B_Reactivity/blob/main/Widefield_10x_ROIs_Flox-Cre_Fish_Pdgfrb.qmd)). We used analogous CellProfiler [outputs](https://osf.io/mb257/files/osfstorage/6938b1dc99d2f7c14649fe9f) and conducted the same statistical modeling strategy. We share the model summaries as .tex tables in the “Tables/tex” component of the OSF repository.

## **Supplementary Method 20: Analysis of Collagen IV (ColIV) in PDGFRβ^KLF4-KO^ mice.**

| **Component** | **Link** |
| --- | --- |
| Figures | Figure 7I-L; Suppl. Figure 10A-C |
| Raw Images | [Widefield_5x_Ipsilateral_KO_CD31-ColIV-Iba1.zip](https://zenodo.org/records/10553084/files/Widefield_5x_Ipsilateral_KO_CD31-ColIV-Iba1.zip?download=1)  [Widefield_10x_ROIs_KO_CD31-ColIV-Iba1.zip](https://zenodo.org/records/10553084/files/Widefield_10x_ROIs_KO_CD31-ColIV-Iba1.zip?download=1) |
| Low-resolution images | [Widefield_10x_Ipsilateral_KO_CD31-ColIV.zip](https://osf.io/mb257/files/r83ea)  [Widefield_5x_Ipsilateral_KO_CD31-ColIV.zip](https://osf.io/mb257/files/r83ea) |
| Image Processing | [Images_Processing/Widefield_5x_Ipsilateral_KO_CD31-ColIV](https://osf.io/9zdc7/files/osfstorage) |
| Raw Data | [DataRaw/Widefield_5x_Ipsilateral_KO_CD31-ColIV](https://osf.io/zvnxa/files/osfstorage)  [Data_Raw/](https://osf.io/zvnxa/)[Widefield_](https://osf.io/mb257/files/osfstorage/6938b1dc99d2f7c14649fe9f)[10x_Ipsilateral_KO_CD31-ColIV](https://osf.io/mb257/files/r83ea) |
| Processed data | [Data_Processed/](https://osf.io/4dpze)[Widefield_](https://osf.io/mb257/files/osfstorage/6938b1dc99d2f7c14649fe9f)[10x_Ipsilateral_KO_CD31-ColIV](https://osf.io/mb257/files/r83ea) |
| Statistical models | [StatisticalModels_brms/Widefield_10x_Ipsilateral_KO_CD31-ColIV](https://osf.io/zvnxa/overview)  [StatisticalModels_brms/Widefield_10x_Ipsilateral_KO_CD31-ColIV_Inten](https://osf.io/zvnxa/files/osfstorage) |
| Analysis notebook | [Widefield_10x_ROIs_KO_CD31-ColIV_Coloc.qmd](https://github.com/elalilab/Stroke_PDGFR-B_Reactivity/blob/main/Widefield_10x_ROIs_KO_CD31-ColIV_Coloc.qmd)  [Widefield_10x_Ipsilateral_KO_CD31-ColIV_Inten.qmd](https://github.com/elalilab/Stroke_PDGFR-B_Reactivity/blob/main/Widefield_10x_Ipsilateral_KO_CD31-ColIV_Inten.qmd) |

We acquired widefield images at 5x and 10x magnification of CD31 / ColIV immunolabeled brain sections (see the [protocol](https://osf.io/u9vpb)) from the ipsilateral hemisphere of PDGFRβ^KLF4-KO^ mice and corresponding controls. We analized the immonolabeling intensity of vascular (CD31^+^) basement membrane Collagen-IV (ColIV) in the whole ipsilateral hemisphere using CellProfiler [outputs](https://osf.io/u5q82). First, we fitted a distributional model to estimate both the mean expression and the group-level variability (sigma) by genotype, accounting for the heterogeneity observed in the data:

$$Area \sim Genotype, sigma \sim Genotype, family = student$$

Next, we fitted a robust linear model to analyze the integrated intensity conditioning on genotype:

$$Intensity \sim Genotype, family = student$$

Model diagnostics and source code are available in the [QN](https://github.com/elalilab/Stroke_PDGFR-B_Reactivity/blob/main/Widefield_5x_Ipsilateral_KO_CD31-ColIV_Inten.qmd). We present the results as full posterior distribution contrast between genotypes using spread_draws from tidybayes). The [posterior summary](https://osf.io/e7gwm/files/s4g8c) is shared as a .tex table in the OSF repository. Secondly, we analyzed vascular-associated Collagen-IV area in the cortex using images taken at 10x. We fitted a linear model to estimate the stained area conditioning on genotype:

$$Area \sim Genotype, family = student$$

We visualized the posterior distributions using stat_halfeye and stat_interval. We share the [model summaries](https://osf.io/97vr6) as .tex tables in the Tables/tex component of the OSF repository.

**References**

[Aktas, M. E., Akbas, E., & Fatmaoui, A. E. (2019). Persistence homology of networks: Methods and applications. *Applied Network Science*, *4*(1), Article 1. https://doi.org/10.1007/s41109-019-0179-3](https://www.zotero.org/google-docs/?YNYKud)

[Berg, S., Kutra, D., Kroeger, T., Straehle, C. N., Kausler, B. X., Haubold, C., Schiegg, M., Ales, J., Beier, T., Rudy, M., Eren, K., Cervantes, J. I., Xu, B., Beuttenmueller, F., Wolny, A., Zhang, C., Koethe, U., Hamprecht, F. A., & Kreshuk, A. (2019). ilastik: Interactive machine learning for (bio)image analysis. *Nature Methods*, *16*(12), 1226–1232. https://doi.org/10.1038/s41592-019-0582-9](https://www.zotero.org/google-docs/?YNYKud)

[Chambers, E. W., de Silva, V., Erickson, J., & Ghrist, R. (2010). Vietoris–Rips Complexes of Planar Point Sets. *Discrete & Computational Geometry*, *44*(1), 75–90. https://doi.org/10.1007/s00454-009-9209-8](https://www.zotero.org/google-docs/?YNYKud)

[Chernik, M. R. (2007). What Is Bootstrapping? In *Bootstrap Methods* (pp. 1–25). John Wiley & Sons, Ltd. https://doi.org/10.1002/9780470192573.ch1](https://www.zotero.org/google-docs/?YNYKud)

[Coelho, L. P. (2013). Mahotas: Open source software for scriptable computer vision. *Journal of Open Research Software*, *1*(1). https://doi.org/10.5334/jors.ac](https://www.zotero.org/google-docs/?YNYKud)

[Hahne, F., LeMeur, N., Brinkman, R. R., Ellis, B., Haaland, P., Sarkar, D., Spidlen, J., Strain, E., & Gentleman, R. (2009). flowCore: A Bioconductor package for high throughput flow cytometry. *BMC Bioinformatics*, *10*(1), 106. https://doi.org/10.1186/1471-2105-10-106](https://www.zotero.org/google-docs/?YNYKud)

[Hajij, M., Wang, B., Scheidegger, C., & Rosen, P. (2018). Visual Detection of Structural Changes in Time-Varying Graphs Using Persistent Homology. *2018 IEEE Pacific Visualization Symposium (PacificVis)*, 125–134. https://doi.org/10.1109/PacificVis.2018.00024](https://www.zotero.org/google-docs/?YNYKud)

[Hunter, J. D. (2007). Matplotlib: A 2D Graphics Environment. *Computing in Science & Engineering*, *9*(3), 90–95. Computing in Science & Engineering. https://doi.org/10.1109/MCSE.2007.55](https://www.zotero.org/google-docs/?YNYKud)

[Lê, S., Josse, J., & Husson, F. (2008). FactoMineR: An R Package for Multivariate Analysis. *Journal of Statistical Software*, *25*, 1–18. https://doi.org/10.18637/jss.v025.i01](https://www.zotero.org/google-docs/?YNYKud)

[Lenth, R. V. (2024). *emmeans: Estimated Marginal Means, aka Least-Squares Means* (Version R package version 1.10.1). https://CRAN.R-project.org/package=emmeans](https://www.zotero.org/google-docs/?YNYKud)

[Löfstedt, T., Brynolfsson, P., Asklund, T., Nyholm, T., & Garpebring, A. (2019). Gray-level invariant Haralick texture features. *PLOS ONE*, *14*(2), e0212110. https://doi.org/10.1371/journal.pone.0212110](https://www.zotero.org/google-docs/?YNYKud)

[Makowski, D., Ben-Shachar, M. S., Patil, I., & Lüdecke, D. (2020). *Estimation of Model-Based Predictions, Contrasts and Means.* https://github.com/easystats/modelbased](https://www.zotero.org/google-docs/?YNYKud)

[Manrique-Castano, D., Bhaskar, D., & ElAli, A. (2024). Dissecting glial scar formation by spatial point pattern and topological data analysis. *Scientific Reports*, *14*(1), 19035. https://doi.org/10.1038/s41598-024-69426-z](https://www.zotero.org/google-docs/?YNYKud)

[Monaco, G., Chen, H., Poidinger, M., Chen, J., de Magalhães, J. P., & Larbi, A. (2016). flowAI: Automatic and interactive anomaly discerning tools for flow cytometry data. *Bioinformatics (Oxford, England)*, *32*(16), 2473–2480. https://doi.org/10.1093/bioinformatics/btw191](https://www.zotero.org/google-docs/?YNYKud)

[Otter, N., Porter, M. A., Tillmann, U., Grindrod, P., & Harrington, H. A. (2017). A roadmap for the computation of persistent homology. *EPJ Data Science*, *6*(1), Article 1. https://doi.org/10.1140/epjds/s13688-017-0109-5](https://www.zotero.org/google-docs/?YNYKud)

[Stirling, D. R., Swain-Bowden, M. J., Lucas, A. M., Carpenter, A. E., Cimini, B. A., & Goodman, A. (2021). CellProfiler 4: Improvements in speed, utility and usability. *BMC Bioinformatics*, *22*(1), 433. https://doi.org/10.1186/s12859-021-04344-9](https://www.zotero.org/google-docs/?YNYKud)

[The GUDHI Project. (2023). *GUDHI User and Reference Manual* (3.9.0). GUDHI Editorial Board. https://gudhi.inria.fr/doc/3.9.0/](https://www.zotero.org/google-docs/?YNYKud)

[Tralie, C., Saul, N., & Bar-On, R. (2018). Ripser.py: A Lean Persistent Homology Library for Python. *Journal of Open Source Software*, *3*(29), 925. https://doi.org/10.21105/joss.00925](https://www.zotero.org/google-docs/?YNYKud)

[Van, P., Jiang, W., Gottardo, R., & Finak, G. (2018). ggCyto: Next generation open-source visualization software for cytometry. *Bioinformatics*, *34*(22), 3951–3953. https://doi.org/10.1093/bioinformatics/bty441](https://www.zotero.org/google-docs/?YNYKud)
