# Supplementary material for: Brain PDGFRβ + Cells Exhibit Diverse Reactive Phenotypes After Stroke Without Requiring KLF4
Source: Glia. 2026 Oct 31;75(1):e70232. doi: 10.1002/glia.70232 (PMC13723038; doi:10.1002/glia.70232)
Supplement: Supplementary file 2 [file GLIA-75-0-s003.docx]

**Supplementary Tables**

Supplementary Table 1	2

Supplementary Table 2	3

Supplementary Table 3	4

Supplementary Table 4	5

Supplementary Table 5	6

Supplementary Table 6	7

Supplementary Table 7	8

Supplementary Table 8	9

Supplementary Table 9	11

Supplementary Table 10	12

Supplementary Table 11	13

Supplementary Table 12	14

Supplementary Table 13	15

Supplementary Table 14	16

Supplementary Table 15	17

Supplementary Table 16	18

Supplementary Table 17	19

Supplementary Table 18	20

Supplementary Table 19	21

Supplementary Table 20	23

Supplementary Table 21	24

Supplementary Table 22	25

Supplementary Table 23	26

Supplementary Table 24	27

Supplementary Table 25	28

Supplementary Table 26	29

Supplementary Table 27	30

Supplementary Table 28	31

Supplementary Table 29	32

Supplementary Table 30	33

Supplementary Table 31	34

Supplementary Table 32	35

Supplementary Table 33	36

Supplementary Table 34	37

Supplementary Table 35	38

Supplementary Table 36	39

Supplementary Table 37	40

**Supplementary Table 1: Hemispheric area following MCAo.** **A)** Posterior summary with parameter estimates (Est.) and 95% credible intervals (2.5% - 97.5%) for the non-linear (splines) model *Area ~ s(DPI, K=5), sigma ~ DPI, family = student*. Parameters for sigma are in the scale. **B)** Model derivatives (estimate_smooth) showing linear changes from 0-23 DPI and 23-30 DPI.

**(A)** Splines model

| **Parameter** | **Est.** | **2.5 %** | **97.5 %** |
| --- | --- | --- | --- |
| Intercept | 11.9 | 11.5 | 12.45 |
| sigma_Intercept | 0.2 | -0.25 | 0.63 |
| sigma_DPI | 0.005 | 0.000 | 0.01 |
| sDPI_1 | 0.079 | -1.91 | 1.99 |
| sds_sDPI_1 | 14.7 | 7.66 | 28.7 |
| Num.Obs. | 49 |  |  |
| R2 | 0.76 |  |  |

**(B)** Model derivatives

| **Start** | **End** | **Length** | **Change** | **Slope** | **R2** |
| --- | --- | --- | --- | --- | --- |
| 0.00 | 23.33 | 0.7 | -7.91 | -0.34 | 0.9 |
| 23.33 | 30.00 | 0.2 | 0.17 | 0.03 | 0.9 |

**Supplementary Table 2: PDGFRβ integrated density following MCAo.** **A)** Posterior summary with parameter estimates (Est.) and 95% credible intervals (2.5% - 97.5%) for the non-linear (splines) model *IntDen ~ s(DPI, K=5), sigma ~ DPI, family = student*. Parameters for sigma are in the scale. **B)** Model derivatives (estimate_smooth) showing linear changes from 0-23 DPI and 23-30 DPI.

**(A)** Splines model

| **Parameter** | **Est.** | **2.5 %** | **97.5 %** |
| --- | --- | --- | --- |
| Intercept | 620 | 533 | 702 |
| sigma_Intercept | 5.4 | 5.2 | 5.6 |
| sDPI_1 | 0.003 | -1.9 | 1.9 |
| sigma_sDPI_1 | 11.06 | 1.52 | 24.6 |
| sds_sDPI_1 | 2084 | 920 | 4734 |
| sds_sigma_sDPI_1 | 10.9 | 1.1 | 46.5 |
| s_sigma_sDPI_1[1] | -5.5 | -14.8 | 1.1 |
| s_sigma_sDPI_1[2] | -1.2 | -19.7 | 14.3 |
| s_sigma_sDPI_1[3] | -2.1 | -5.1 | 0.6 |
| Num.Obs. | 49 |  |  |
| R2 | 0.59 |  |  |
| R2 Adj. | 0.57 |  |  |

**(B)** Model derivatives

| **Start** | **End** | **Length** | **Change** | **Slope** | **R2** |
| --- | --- | --- | --- | --- | --- |
| 0 | 23 | 0.66 | 841 | 36.07 | 0.84 |
| 23 | 30 | 0.24 | -29.4 | -4.42 | 0.84 |

**Supplementary Table 3:** **PDGFRβ integrated density following MCAo (conditioning on hemispheric area).** **A)** Posterior summary with parameter estimates (Est.) and 95% credible intervals (2.5% - 97.5%) for the non-linear (splines) model *Pdgfrb_IntDen ~ t2(DPI,Tissue_Area), family = Gaussian*.

| **Parameter** | **Est.** | **2.5 %** | **97.5 %** |
| --- | --- | --- | --- |
| b_Intercept | 620 | 512 | 745 |
| bs_t2DPITissue_Area_1 | -206 | -471 | 118 |
| bs_t2DPITissue_Area_2 | -119 | -460 | 133 |
| bs_t2DPITissue_Area_3 | 85 | -391 | 463 |
| sds_t2DPITissue_Area_1 | 563 | 18 | 1763 |
| sds_t2DPITissue_Area_2 | 621 | 17 | 2382 |
| sds_t2DPITissue_Area_3 | 876 | 34 | 2685 |
| sigma | 268 | 215 | 336 |
| Num.Obs. | 49 |  |  |
| R2 | 0.63 |  |  |
| R2 Adj. | 0.56 |  |  |

**Supplementary Table 4:** **GFAP integrated density following MCAo.** **A)** Posterior summary with parameter estimates (Est.) and 95% credible intervals (2.5% - 97.5%) for the non-linear (splines) model *IntDen ~ s(DPI, K=5), sigma ~ DPI, family = student*. Parameters for sigma are shown in the scale. **B)** Model derivatives (estimate_smooth) showing linear changes from 0-16 DPI and 16-30 DPI.

**(A)** Splines model

| **Parameter** | **Est.** | **2.5 %** | **97.5 %** |
| --- | --- | --- | --- |
| b_Intercept | 490 | 381 | 615 |
| b_sigma_Intercept | 5.2 | 5.08 | 5.5 |
| bs_sDPI_1 | 0.003 | -1.9 | 1.9 |
| bs_sigma_sDPI_1 | 26.4 | 6.1 | 49.6 |
| sds_sDPI_1 | 1698 | 817 | 3576 |
| sds_sigma_sDPI_1 | 22.7 | 4.9 | 85.8 |
| s_sigma_sDPI_1[1] | -14.7 | -25.9 | -4.2 |
| s_sigma_sDPI_1[2] | 5.1 | -19.2 | 33.3 |
| s_sigma_sDPI_1[3] | -2.9 | -8.1 | 1.7 |
| Num.Obs. | 49 |  |  |
| R2 | 0.51 |  |  |
| R2 Adj. | 0.18 |  |  |

**(B)** Model derivatives

| **Start** | **End** | **Length** | **Change** | **Slope** | **R2** |
| --- | --- | --- | --- | --- | --- |
| 0 | 16 | 0.46 | 835.13 | 50.11 | 0.24 |
| 16 | 30 | 0.44 | -480.65 | -36.05 | 0.24 |

**Supplementary Table 5: GFAP integrated density following MCAo (conditioning on hemispheric area).** Posterior summary with parameter estimates (Est.) and 95% credible intervals (2.5% - 97.5%) for the non-linear (splines) model *Gfap_IntDen ~ t2(DPI,Tissue_Area), family = Gaussian*.

| **Parameters** | **Est.** | **2.5 %** | **97.5 %** |
| --- | --- | --- | --- |
| b_Intercept | 509 | 425 | 605 |
| bs_t2DPITissue_Area_1 | 229 | 17 | 451 |
| bs_t2DPITissue_Area_2 | -325 | -532 | -126 |
| bs_t2DPITissue_Area_3 | -369 | -645 | -108 |
| sds_t2DPITissue_Area_1 | 360 | 17 | 1078 |
| sds_t2DPITissue_Area_2 | 350 | 11 | 1288 |
| sds_t2DPITissue_Area_3 | 411 | 14 | 1405 |
| sigma | 242 | 196 | 301 |
| Num.Obs. | 49 |  |  |
| R2 | 0.45 |  |  |
| R2 Adj. | 0.37 |  |  |

**Supplementary Table 6:** **PDGFRβ^high^ likelihood following MCAo.** Posterior summary with parameter estimates (Est.) and 95% credible intervals (2.5% - 97.5%) for the binomial model *Pdgfrb_high | trials (Pdgfrb_Total) ~ s(DPI, k =5), family = binomial*.

| **Parameters** | **Est.** | **2.5 %** | **97.5 %** |
| --- | --- | --- | --- |
| b_Intercept | 1.130 | 1.114 | 1.146 |
| bs_sDPI_1 | 25.6 | 23.9 | 27.3 |
| sds_sDPI_1 | 9.5 | 5.1 | 18.4 |
| Num.Obs. | 49 |  |  |
| R2 | 0.97 |  |  |

**Supplementary Table 7:** **GFAP convex hull following MCAo.** Posterior summary with parameter estimates (Est.) and 95% credible intervals (2.5% - 97.5%) for the linear model *Healthy_Ratio ~ DPI, family = student*.

| **Parameter** | **Est.** | **2.5 %** | **97.5 %** |
| --- | --- | --- | --- |
| b_Intercept | 61.1 | 56.4 | 65.9 |
| b_DPI | -0.100 | -0.38 | 0.17 |
| sigma | 8.20 | 6.33 | 10.47 |
| Num.Obs. | 38 |  |  |
| R2 | 0.02 |  |  |

**Supplementary Table 8: PDGFRβ counts in tesselated GFAP regions.** **A)** Posterior summary with parameter estimates (Est.) and 95% credible intervals (2.5% - 97.5%) for the model *Pdgfrb ~ Region x Gfap, family = hurdle_lognormal()*. **B-D)** Posterior summary for models by region *Pdgfrb ~ DPI x Gfap, family = hurdle_lognormal()*.

**(A)** Model for all regions

| **Paremeters** | **Est.** | **2.5 %** | **97.5 %** |
| --- | --- | --- | --- |
| b_Intercept | 3.7 | 3.5 | 4.0 |
| b_Striatum | 0.4 | 0.1 | 0.8 |
| b_Cortex | 1.06 | 0.66 | 1.45 |
| b_Gfap_High | -0.46 | -0.83 | -0.09 |
| b_Striatum × Gfap_High | 0.45 | -0.06 | 0.97 |
| b_RegionCortex × Gfap_High | -0.64 | -1.19 | -0.06 |
| sigma | 1.20 | 1.10 | 1.31 |
| Num.Obs. | 262 |  |  |
| R2 | 0.43 |  |  |

**(B)** Model for perilesion

| **Parameters** | **Est.** | **2.5 %** | **97.5 %** |
| --- | --- | --- | --- |
| b_Intercept | 3.53 | 3.08 | 3.99 |
| b_DPI7D | 0.29 | -0.35 | 0.93 |
| b_DPI14D | 0.51 | -0.09 | 1.12 |
| b_DPI30D | 0.09 | -0.53 | 0.73 |
| b_Gfap_High | -0.40 | -1.05 | 0.25 |
| b_DPI7D × Gfap_High | 0.48 | -0.41 | 1.37 |
| b_DPI14D × Gfap_High | 0.07 | -0.79 | 0.93 |
| b_DPI30D × Gfap_High | -0.75 | -1.62 | 0.13 |
| sigma | 0.99 | 0.84 | 1.17 |
| Num.Obs. | 88 |  |  |
| R2 | 0.391 |  |  |

**(C)** Model for striatum

| **Parameters** | **Est.** | **2.5 %** | **97.5 %** |
| --- | --- | --- | --- |
| b_Intercept | 3.38 | 2.95 | 3.81 |
| b_DPI7D | 0.88 | 0.30 | 1.44 |
| b_DPI14D | 1.19 | 0.62 | 1.75 |
| b_DPI30D | 1.27 | 0.70 | 1.85 |
| b_Gfap_High | -1.47 | -2.08 | -0.87 |
| b_DPI7D × Gfap_High | 1.47 | 0.67 | 2.29 |
| b_DPI14D × Gfap_High | 2.05 | 1.25 | 2.86 |
| b_DPI30D × Gfap_High | 2.01 | 1.21 | 2.82 |
| sigma | 0.92 | 0.79 | 1.09 |
| Num.Obs. | 88 |  |  |
| R2 | 0.241 |  |  |

**(D)** Model for cortex

| **Parameters** | **Est.** | **2.5 %** | **97.5 %** |
| --- | --- | --- | --- |
| b_Intercept | -12.51 | -49.35 | 0.74 |
| b_DPI7D | 16.45 | 3.20 | 53.36 |
| b_DPI14D | 17.98 | 4.72 | 54.96 |
| b_DPI30D | 18.48 | 5.21 | 55.41 |
| b_Gfap_High | -24.25 | -76.36 | -5.43 |
| b_DPI7D × Gfap_High | 21.41 | 2.51 | 73.70 |
| b_DPI14D × Gfap_High | 22.79 | 3.95 | 74.85 |
| b_DPI30D × Gfap_High | 23.72 | 4.92 | 75.88 |
| shape | 0.76 | 0.54 | 1.04 |
| Num.Obs. | 86 |  |  |
| R2 | 0.575 |  |  |

**Supplementary Table 9: PDGFRβ^high^ likelihood in striatal-only lesions**. Posterior summary with parameter estimates (Est.) and 95% credible intervals (2.5% - 97.5%) for the binomial model *Pdgfrb_high | trials(Pdgfrb_Total) ~ DPI, family = binomial*.

| **Parameters** | **Est.** | **2.5 %** | **97.5 %** |
| --- | --- | --- | --- |
| b_Intercept | -0.65 | -0.73 | -0.57 |
| b_DPI | 0.02 | 0.02 | 0.02 |
| Num.Obs. | 14 |  |  |
| R2 | 0.640 |  |  |

**Supplementary Table 10: Parenchymal PDGFRβ likelihood following MCAo**. **A-C)** Posterior summary with parameter estimates (Est.) and 95% credible intervals (2.5% - 97.5%) for the binomial models *Pdgfrb_Parenchymal | trials (Pdgfrb_Total) ~ DPI, family = binomial* for perilesion, striatum and cortex, respectively.

**(A)** Model for perilesion

| **Parameters** | **Est.** | **2.5 %** | **97.5 %** |
| --- | --- | --- | --- |
| b_Intercept | -2.84 | -2.98 | -2.70 |
| bs_sDPI2_1 | 0.059 | -3.93 | 4.71 |
| sds_sDPI2_1 | 3.22 | 1.13 | 7.70 |
| Num.Obs. | 43 |  |  |
| R2 | 0.207 |  |  |

**(B)** Model for striatum

| **Parameters** | **Est.** | **2.5 %** | **97.5 %** |
| --- | --- | --- | --- |
| b_Intercept | -1.37 | -1.44 | -1.30 |
| bs_sDPI2_1 | 4.19 | 1.08 | 7.64 |
| sds_sDPI2_1 | 2.92 | 0.96 | 7.07 |
| Num.Obs. | 43 |  |  |
| R2 | 0.69 |  |  |

**(C)** Model for cortex

| **Parameters** | **Est.** | **2.5 %** | **97.5 %** |
| --- | --- | --- | --- |
| b_Intercept | -0.93 | -0.97 | -0.89 |
| bs_sDPI2_1 | 17.37 | 15.07 | 19.75 |
| sds_sDPI2_1 | 8.71 | 4.42 | 18.52 |
| Num.Obs. | 40 |  |  |
| R2 | 0.83 |  |  |

**Supplementary Table 11:** **FACS-sorted PDGFRβ^+^ cells derived from contralateral and ipsilateral hemispheres**. **A-B)** Posterior summary with parameter estimates (Est.) and 95% credible intervals (2.5% - 97.5%) for the linear models *Cells ~ Hemisphere, family = student* estimating the percentage of total PDGFRβ+ cells **(A)** and dead PDGFRβ+ cells **(B)**.

**(A)** Model for the number of PDGFRβ^+^ cells

| **Parameter** | **Est.** | **2.5 %** | **97.5 %** |
| --- | --- | --- | --- |
| b_Intercept | 0.062 | 0.034 | 0.091 |
| b_HemisphereIpsi | 0.074 | 0.033 | 0.114 |
| sigma | 0.030 | 0.017 | 0.054 |
| Num.Obs. | 10 |  |  |
| R2 | 0.72 |  |  |

**(B)** Model for the number of PDGFRβ^+^ dead cells

| **Parameter** | **Est.** | **2.5 %** | **97.5 %** |
| --- | --- | --- | --- |
| b_Intercept | 0.106 | 0.004 | 0.209 |
| b_HemisphereIpsi | 0.137 | -0.006 | 0.299 |
| sigma | 0.108 | 0.044 | 0.208 |
| Num.Obs. | 10 |  |  |
| R2 | 0.33 |  |  |

**Supplementary Table 12:** **Parenchymal PDGFRβ likelihood in striatal-restricted lesions**. Posterior summary with parameter estimates (Est.) and 95% credible intervals (2.5% - 97.5%) for the binomial model *Pdgfrb_Parenchymal | trials (Pdgfrb_Total) ~ DPI, family = binomial* in striatal-only lesions.

| **Parameters** | **Est.** | **2.5 %** | **97.5 %** |
| --- | --- | --- | --- |
| b_Intercept | -1.58 | -1.79 | -1.38 |
| b_DPI30D | 0.38 | 0.15 | 0.60 |
| b_Cortex | 0.52 | 0.30 | 0.74 |
| b_DPI30D × Cortex | -0.68 | -0.93 | -0.43 |
| Num.Obs. | 38 |  |  |
| R2 | 0.653 |  |  |

**Supplementary Table 13:** **Number of PDGFRβ^+^ cells in striatal-only and cortico-striatal lesions**. Posterior summary with parameter estimates (Est.) and 95% credible intervals (2.5% - 97.5%) for the model *Pdgfrb_Total ~ DPI x Lesion, sigma ~ DPI x Lesion, family = student* in striatal-only lesions.

| **Parameters** | **Est.** | **2.5 %** | **97.5 %** |
| --- | --- | --- | --- |
| b_Intercept | 159 | 25 | 269 |
| b_sigma_Intercept | 4.40 | 3.21 | 5.66 |
| b_DPI30D | 64.4 | -61.2 | 211.6 |
| b_Cortex | 59.7 | -82.4 | 229.5 |
| b_DPI30D × Cortex | -20.3 | -228.2 | 180.2 |
| b_sigma_DPI30D | -0.07 | -1.46 | 1.11 |
| b_sigma_Cortex | 0.37 | -1.16 | 1.51 |
| b_sigma_DPI30D × Cortex | 0.50 | -0.90 | 2.17 |
| Num.Obs. | 38 |  |  |
| R2 | 0.065 |  |  |

**Supplementary Table 14: Number of parenchymal PDGFRβ^+^ cells in sham and MCAo mice**. Posterior summary with parameter estimates (Est.) and 95% credible intervals (2.5% - 97.5%) for the model *Parenchymal ~ DPI x Condition, family = student* in sham and MCAo mice.

| **Parameters** | **Est.** | **2.5 %** | **97.5 %** |
| --- | --- | --- | --- |
| b_Intercept | 1.006 | 0.44 | 1.52 |
| b_DPI7D | -0.001 | -0.75 | 0.73 |
| b_DPI14D | 0.29 | -0.40 | 0.98 |
| b_DPI30D | 0.04 | -0.81 | 0.85 |
| b_MCAo | 0.73 | 0.16 | 1.34 |
| b_DPI7D × MCAo | 0.11 | -0.68 | 0.91 |
| b_DPI14D × MCAo | -1.02 | -1.83 | -0.20 |
| b_DPI30D × MCAo | 0.10 | -0.74 | 1.02 |
| Num.Obs. | 60 |  |  |
| R2 | 0.154 |  |  |

**Supplementary Table 15: PDGFRβ^+^ cells proliferation in the ischemic hemisphere following MCAo.** **A)** Posterior summary with parameter estimates (Est.) and 95% credible intervals (2.5% - 97.5%) for the non-linear (splines) model *Percentage_Pdgfrb ~ s(DPI_Cont, k = 5), family = lognormal*. **B)** Model derivatives (estimate_smooth) showing linear changes from 0-3 DPI and 3-30 DPI.

**(A)** Splines model

| **Parameters** | **Est.** | **2.5 %** | **97.5 %** |
| --- | --- | --- | --- |
| b_Intercept | 0.74 | 0.59 | 0.88 |
| bs_sDPI_Cont_1 | 65.7 | 48.6 | 80.9 |
| sds_sDPI_Cont_1 | 33.7 | 16.6 | 67.4 |
| sigma | 0.52 | 0.42 | 0.66 |
| Num.Obs. | 50 |  |  |
| R2 | 0.528 |  |  |

**(B)** Model derivatives

| **Start** | **End** | **Length** | **Change** | **Slope** | **R2** |
| --- | --- | --- | --- | --- | --- |
| 0 | 3 | 0.08 | 6.87 | 2.06 | 0.24 |
| 3 | 30 | 0.82 | -6.10 | -0.23 | 0.24 |

**Supplementary Table 16:** **PDGFRβ^+^ cells proliferation in defined ROIs following MCAo.** **A)** Posterior summary with parameter estimates (Est.) and 95% credible intervals (2.5% - 97.5%) for the model *Cells ~ DPI x Region, family = negbinomial* showing the total amount of colocalized cells. **B)** The same estimates for the model *Vascular | trials (Total) ~ DPI x Region, family = binomial* showing the proportion of vascular PDGFRβ^+^ cells relative to the total number of PDGFRβ^+^ cells.

**(A)** Model for total PDGFRβ/Ki67^+^ cells

| **Parameters** | **Est.** | **2.5 %** | **97.5 %** |
| --- | --- | --- | --- |
| b_Intercept | -1.55 | -3.46 | -0.01 |
| b_DPI7D | 0.37 | -1.56 | 2.51 |
| b_RegionStr | 2.22 | 0.43 | 4.31 |
| b_RegionCtx | 2.75 | 0.99 | 4.82 |
| b_DPI7D × RegionStr | -0.95 | -3.45 | 1.41 |
| b_DPI7D × RegionCtx | 0.20 | -2.19 | 2.48 |
| shape | 1.01 | 0.45 | 2.04 |
| Num.Obs. | 59 |  |  |
| R2 | 0.391 |  |  |

**(B)** Model for vascular PDGFRβ/Ki67^+^ cells

| **Parameters** | **Est.** | **2.5 %** | **97.5 %** |
| --- | --- | --- | --- |
| b_Intercept | 95.8 | 3.3 | 437.3 |
| b_DPI7D | -57.3 | -346.1 | 72.3 |
| b_RegionStr | -92.8 | -434.7 | -0.14 |
| b_RegionCtx | -94.4 | -436.3 | -1.7 |
| b_DPI7D × RegionStr | 56.2 | -73.8 | 343.3 |
| b_DPI7D × RegionCtx | 56.06 | -73.68 | 345 |
| Num.Obs. | 31 |  |  |
| R2 | 0.813 |  |  |

**Supplementary Table 17:** **Haralick features of PDGFRβ^+^ cells**. Posterior summary with parameter estimates (Est.) and 95% credible intervals (2.5% - 97.5%) for the logistic model *Region ~ entropy + contrast + IDM, family = categorical*.

| **Parameters** | **Est.** | **2.5 %** | **97.5 %** |
| --- | --- | --- | --- |
| b_muStriatum_Intercept | 0.49 | -0.011 | 1.04 |
| b_muCortex_Intercept | 0.25 | -0.29 | 0.82 |
| b_muStriatum_entropy | 2.54 | 0.54 | 4.68 |
| b_muStriatum_contrast | -0.79 | -1.92 | 0.27 |
| b_muStriatum_IDM | 1.43 | -0.13 | 3.09 |
| b_muCortex_entropy | 5.26 | 3.21 | 7.55 |
| b_muCortex_contrast | -1.12 | -2.22 | -0.03 |
| b_muCortex_IDM | 2.92 | 1.13 | 4.83 |
| Num.Obs. | 146 |  |  |

**Supplementary Table 18: PDGFRβ stained area in defined ROIs**. Posterior summary with parameter estimates (Est.) and 95% credible intervals (2.5% - 97.5%) for the binomial model *Area ~ DPI_cont x Region, family = student*.

| **Parameters** | **Est.** | **2.5 %** | **97.5 %** |
| --- | --- | --- | --- |
| b_Intercept | 143 | 123 | 164 |
| b_DPI | 10.8 | 9.6 | 12.0 |
| b_Striatum | -0.3 | -36.6 | 38.9 |
| b_Cortex | -9.3 | -40.3 | 22.4 |
| b_DPI × Striatum | 14.6 | 12.6 | 16.6 |
| b_DPI × Cortex | 4.5 | 2.4 | 6.4 |
| sigma | 58.1 | 43.4 | 76.8 |
| Num.Obs. | 143 |  |  |
| R2 | 0.698 |  |  |

**Supplementary Table 19: Topological assessment of PDGFRβ^+^ cells following injury:** Wasserstein (**A-B)** and Bottleneck **(C-D)** distances for 0- and 1-dimension homology. We boostraped the estimates (1000 replications) to show the median Betti curve distance with 95% credible intervals (2.5% - 97.5%).

**(A)** Wasserstein distance - 0D homology

| **Day1** | **Day2** | **Region** | **Median** | **2.5 %** | **97.5 %** |
| --- | --- | --- | --- | --- | --- |
| 0D | 3D | Ctx | 211 | 150 | 323 |
| 3D | 7D | Ctx | 603 | 507 | 669 |
| 7D | 14D | Ctx | 208 | 146 | 280 |
| 14D | 30D | Ctx | 156 | 119 | 225 |
| 0D | 3D | Str | 385 | 168 | 548 |
| 3D | 7D | Str | 348 | 222 | 784 |
| 7D | 14D | Str | 227 | 184 | 315 |
| 14D | 30D | Str | 243 | 177 | 318 |
| 3D | 7D | Peri | 230 | 172 | 308 |
| 7D | 14D | Peri | 182 | 150 | 229 |
| 14D | 30D | Peri | 239 | 189 | 304 |

**(B)** Wasserstein distance - 1D homology

| **Day1** | **Day2** | **Region** | **Median** | **2.5 %** | **97.5 %** |
| --- | --- | --- | --- | --- | --- |
| 0D | 3D | Ctx | 126 | 111 | 168 |
| 3D | 7D | Ctx | 216 | 194 | 232 |
| 7D | 14D | Ctx | 135 | 111 | 166 |
| 14D | 30D | Ctx | 122 | 94 | 153 |
| 0D | 3D | Str | 134 | 115 | 162 |
| 3D | 7D | Str | 158 | 129 | 194 |
| 7D | 14D | Str | 146 | 137 | 171 |
| 14D | 30D | Str | 166 | 143 | 183 |
| 3D | 7D | Peri | 127 | 111 | 159 |
| 7D | 14D | Peri | 121 | 106 | 132 |
| 14D | 30D | Peri | 138 | 122 | 158 |

**(C)** Bottleneck distance - 0D homology

| **Day1** | **Day2** | **Region** | **Median** | **2.5 %** | **97.5 %** |
| --- | --- | --- | --- | --- | --- |
| 0D | 3D | Str | 127 | 45 | 320 |
| 3D | 7D | Str | 112 | 70 | 438 |
| 7D | 14D | Str | 68 | 48 | 85 |
| 14D | 30D | Str | 62 | 50 | 78 |
| 0D | 3D | Ctx | 56 | 25 | 85 |
| 3D | 7D | Ctx | 116 | 87 | 151 |
| 7D | 14D | Ctx | 35 | 26 | 74 |
| 14D | 30D | Ctx | 27 | 20 | 36 |
| 3D | 7D | Peri | 60 | 48 | 80 |
| 7D | 14D | Peri | 44 | 34 | 55 |
| 14D | 30D | Peri | 58 | 44 | 71 |

**(D)** Bottleneck distance - 1D homology

| **Day1** | **Day2** | **Region** | **Median** | **2.5 %** | **97.5 %** |
| --- | --- | --- | --- | --- | --- |
| 0D | 3D | Str | 75 | 67 | 127 |
| 3D | 7D | Str | 86 | 71 | 127 |
| 7D | 14D | Str | 71 | 65 | 82 |
| 14D | 30D | Str | 82 | 66 | 102 |
| 0D | 3D | Ctx | 64 | 46 | 76 |
| 3D | 7D | Ctx | 74 | 66 | 87 |
| 7D | 14D | Ctx | 59 | 47 | 66 |
| 14D | 30D | Ctx | 48 | 38 | 69 |
| 3D | 7D | Peri | 64 | 47 | 94 |
| 7D | 14D | Peri | 60 | 49 | 70 |
| 14D | 30D | Peri | 64 | 57 | 75 |

**Supplementary Table 20:** **Spatial intensity of KLF4^+^ nuclei following injury.** Posterior summary with parameter estimates (Est.) and 95% credible intervals (2.5% - 97.5%) for the model *Intensity ~ 0 + DPI, sigma ~ 0 + DPI, family = student*. Note that the sigma coefficients are in the log scale.

| **Parameters** | **Est.** | **2.5 %** | **97.5 %** |
| --- | --- | --- | --- |
| b_DPI_0D | 5214 | 4756 | 5667 |
| b_DPI_3D | 3281 | 2966 | 3658 |
| b_DPI_7D | 4177 | 3652 | 4729 |
| b_DPI_14D | 5787 | 4652 | 6835 |
| b_DPI_30D | 4928 | 4427 | 5439 |
| b_sigma_DPI_0D | 6.41 | 5.81 | 7.06 |
| b_sigma_DPI_3D | 6.09 | 5.38 | 6.77 |
| b_sigma_DPI_7D | 6.74 | 6.28 | 7.27 |
| b_sigma_DPI_14D | 7.63 | 7.19 | 8.13 |
| b_sigma_DPI_30D | 6.73 | 6.27 | 7.23 |
| Num.Obs. | 53 |  |  |
| R2 | 0.35 |  |  |

**Supplementary Table 21:** **Staining intensity of KLF4^+^ nuclei following injury.** Population-level posterior summary with parameter estimates (Est.) and 95% credible intervals (2.5% - 97.5%) for the multilevel model *Intensity ~ 0 + DPI + (1 | MouseID), family = hurdle_lognormal*. Note that the group-level (mouse-level) coefficients are shown in the respective QN.

| **Parameter** | **Est.** | **2.5 %** | **97.5%** |
| --- | --- | --- | --- |
| 0 DPI | -4.79 | -4.94 | -4.64 |
| 3 DPI | -4.8 | -4.94 | -4.66 |
| 7 DPI | -4.63 | -4.76 | -4.51 |
| 14 DPI | -4.52 | -4.65 | -4.4 |
| 30 DPI | -4.64 | -4.76 | -4.51 |

**Supplementary Table 22:** **Staining intensity of KLF4^+^ nuclei relative to the x-coordinates.** Population-level posterior summary with parameter estimates (Est.) and 95% credible intervals (2.5% - 97.5%) for the multilevel model *Intensity ~ 0 + DPI x Scaled_CenterX + (1 | MouseID), family = lognormal*. Note that the group-level (mouse-level) coefficients are shown in the respective QN.

| **Parameter** | **Est.** | **2.5 %** | **97.5 %** |
| --- | --- | --- | --- |
| 0 DPI | -4.8 | -4.95 | -4.65 |
| 3 DPI | -4.84 | -4.99 | -4.7 |
| 7 DPI | -4.63 | -4.75 | -4.5 |
| 14 DPI | -4.42 | -4.54 | -4.3 |
| 30 DPI | -4.59 | -4.71 | -4.46 |
| Scaled_x | 0.06 | -0.01 | 0.14 |
| 3 DPI:Scaled_x | 0.06 | -0.05 | 0.16 |
| 7 DPI:Scaled_x | -0.1 | -0.21 | 0.01 |
| 14 DPI:Scaled_x | -0.54 | -0.67 | -0.41 |
| 30 DPI:Scaled_x | -0.31 | -0.47 | -0.15 |

**Supplementary Table 23:** **KLF4/PDGFRβ co-localization in defined ROIs.** Posterior summary with parameter estimates (Est.) and 95% credible intervals (2.5% - 97.5%) for the model *Coloc | trials(Klf4) ~ DPI x Region, family = binomial*.

| **Parameter** | **Est.** | **2.5 %** | **97.5 %** |
| --- | --- | --- | --- |
| b_Intercept | -2.27 | -2.41 | -2.13 |
| b_14D | 0.06 | 0.01 | 0.16 |
| b_30D | 0.12 | 0.02 | 0.28 |
| b_Perilesion | 0.07 | 0.01 | 0.17 |
| b_Injury | 0.52 | 0.37 | 0.66 |
| b_14D × Perilesion | 0.11 | 0.01 | 0.27 |
| b_30D × Perilesion | 0.07 | 0.01 | 0.20 |
| b_14D × Injury | 0.05 | 0.008 | 0.14 |
| b_30D × Injury | 0.34 | 0.15 | 0.53 |
| Num.Obs. | 64 |  |  |
| R2 | 0.595 |  |  |

**Supplementary Table 24: Spatial intensity of KLF4^+^ nuclei in PDGFRβ^KLF4-KO^ mice following injury.** Posterior summary with parameter estimates (Est.) and 95% credible intervals (2.5% - 97.5%) for the model *Intensity ~ Genotype, sigma ~ Genotype, family = student*.

| **Parameter** | **Est.** | **2.5 %** | **97.5 %** |
| --- | --- | --- | --- |
| b_Intercept | 9478 | 8435 | 10475 |
| b_sigma_Intercept | 7.2 | 6.7 | 7.9 |
| b_GenotypeKO | 258 | -805 | 1392 |
| b_sigma_GenotypeKO | -0.5 | -1.3 | 0.2 |
| Num.Obs. | 19 |  |  |
| R2 | 0.03 |  |  |

**Supplementary Table 25:** **Hemispheric ratio in PDGFRβ^KLF4-KO^ mice.** Posterior summary with parameter estimates (Est.) and 95% credible intervals (2.5% - 97.5%) for the model *Shrinkage ~ Genotype, family = student*.

| **Parameters** | **Est.** | **2.5 %** | **97.5 %** |
| --- | --- | --- | --- |
| b_Intercept | 0.76 | 0.71 | 0.81 |
| b_GenotypeKO | 0.02 | -0.04 | 0.09 |
| sigma | 0.06 | 0.04 | 0.10 |
| Num.Obs. | 18 |  |  |
| R2 | 0.04 |  |  |

**Supplementary Table 26: GFAP integrated density in PDGFRβ^KLF4-KO^ mice.** Posterior summary with parameter estimates (Est.) and 95% credible intervals (2.5% - 97.5%) for the model *Gfap ~ Genotype, family = student*.

| **Parameters** | **Est.** | **2.5 %** | **97.5 %** |
| --- | --- | --- | --- |
| b_Intercept | 763 | 681 | 844 |
| b_GenotypeKO | 122 | 12 | 233 |
| sigma | 107 | 73 | 158 |
| Num.Obs. | 18 |  |  |
| R2 | 0.26 |  |  |

**Supplementary Table 27: PDGFRβ integrated density in PDGFRβ^KLF4-KO^ mice.** Posterior summary with parameter estimates (Est.) and 95% credible intervals (2.5% - 97.5%) for the model *Pdgfrb ~ Genotype, family = student*.

| **Parameters** | **Est.** | **2.5 %** | **97.5 %** |
| --- | --- | --- | --- |
| b_Intercept | 539 | 473 | 601 |
| b_GenotypeKO | -148 | -239 | -57 |
| sigma | 89 | 60 | 130 |
| Num.Obs. | 18 |  |  |
| R2 | 0.43 |  |  |

**Supplementary Table 28:** **PDGFRβ integrated density in PDGFRβ^KLF4-KO^ mice with additional predictors.** Posterior summary with parameter estimates (Est.) and 95% credible intervals (2.5% - 97.5%) for the models a) *Pdgfrb ~ Genotype x Shrinkage, family = student* and b) *Pdgfrb ~ Genotype x Ipsilateral_Scaled, family student*.

**(A)** Model conditioning on brain shrinkage

| **Parameters** | **Est.** | **2.5 %** | **97.5 %** |
| --- | --- | --- | --- |
| b_Intercept | 447 | -250 | 1134 |
| b_GenotypeKO | -766 | -2060 | 567 |
| b_Shrinkage | 117 | -767 | 998 |
| b_GenotypeKO × Shrinkage | 787 | -912 | 2418 |
| sigma | 89 | 58 | 135 |
| Num.Obs. | 18 |  |  |
| R2 | 0.49 |  |  |

**(B)** Model conditioning on ipsilateral area

| **Parameter** | **Est.** | **2.5 %** | **97.5 %** |
| --- | --- | --- | --- |
| b_Intercept | 537 | 470 | 604 |
| b_GenotypeKO | -145 | -239 | -52 |
| b_Ipsilateral_Scaled | 29 | -35 | 92 |
| b_GenotypeKO × Ipsilateral_Scaled | -19 | -113 | 77 |
| sigma | 92 | 60 | 137 |
| Num.Obs. | 18 |  |  |
| R2 | 0.47 |  |  |

**Supplementary Table 29:** **PDGFRβ and GFAP integrated density in PDGFRβ^KLF4-KO^ mice (intraperitoneal injection prior to ischemia).** Posterior summary with parameter estimates (Est.) and 95% credible intervals (2.5% - 97.5%) for the models a) *Pdgfrb ~ Genotype, family = student* and b) *Gfap ~ Genotype, family student*.

**(A)** Model for PDGFRβ IntDen

| **Parameters** | **Est.** | **2.5 %** | **97.5 %** |
| --- | --- | --- | --- |
| b_Intercept | 72.3 | 27.8 | 122.6 |
| b_GenotypeKO | 18.3 | -41.0 | 74.4 |
| sigma | 75.1 | 48.0 | 103.9 |
| Num.Obs. | 34 |  |  |
| R2 | 0.019 |  |  |

**(B)** Model for GFAP IntDen

| **Parameters** | **Est.** | **2.5 %** | **97.5 %** |
| --- | --- | --- | --- |
| b_Intercept | 446.6 | 366.9 | 526.0 |
| b_GenotypeKO | -54.0 | -162.4 | 58.1 |
| sigma | 144.3 | 94.1 | 196.9 |
| Num.Obs. | 34 |  |  |
| R2 | 0.031 |  |  |

**Supplementary Table 30:** **Number of PDGFRβ^+^ nuclei in PDGFRβ^KLF4-KO^ mice.** Posterior summary with parameter estimates (Est.) and 95% credible intervals (2.5% - 97.5%) for the multilevel model a) *Counts ~ Genotype + (1 | MouseID), family = negbinomial*. The values are presented in the log scale.

| **Parameters** | **Est.** | **2.5 %** | **97.5 %** |
| --- | --- | --- | --- |
| b_Intercept | 4.82 | 4.61 | 5.04 |
| b_GenotypeKO | -0.10 | -0.38 | 0.16 |
| sd_MouseID_Intercept | 0.19 | 0.02 | 0.37 |
| shape | 12.2 | 6.5 | 20.5 |
| Num.Obs. | 47 |  |  |
| R2 | 0.30 |  |  |

**Supplementary Table 31: PDGFRβ^+^ labeling intensity in PDGFRβ^KLF4-KO^ mice.** Posterior summary with parameter estimates (Est.) and 95% credible intervals (2.5% - 97.5%) for the multilevel model a) *Intensity ~ Area x Genotype + (1 | MouseID), family = student*.

| **Parameters** | **Est.** | **2.5 %** | **97.5 %** |
| --- | --- | --- | --- |
| b_Intercept | 0.29 | 0.06 | 0.54 |
| b_Area | 0.85 | 0.83 | 0.87 |
| b_GenotypeKO | -0.44 | -0.83 | -0.04 |
| b_Area:GenotypeKO | -0.32 | -0.34 | -0.29 |
| sd_MouseID_Intercept | 0.38 | 0.260 | 0.57 |
| sigma | 0.19 | 0.18 | 0.20 |
| Num.Obs. | 5588 |  |  |
| R2 | 0.81 |  |  |

**Supplementary Table 32:** **Number of PDGFRβ^+^ nuclei in PDGFRβ^Cre^ and KLF4^Flox^ mice.** Posterior summary with parameter estimates (Est.) and 95% credible intervals (2.5% - 97.5%) for the multilevel model *Cells ~ Genotype + (1 | MouseID), family = student*.

| **Parameters** | **Est.** | **2.5 %** | **97.5 %** |
| --- | --- | --- | --- |
| b_Intercept | 167 | 146 | 188 |
| b_GenotypeCre | -51 | -78 | -24 |
| b_GenotypeFlox | -2.5 | -33 | 27 |
| sd_MouseID_Intercept | 10 | 0.4 | 25 |
| sigma | 10 | 1.1 | 26 |
| Num.Obs. | 10 |  |  |
| R2 | 0.883 |  |  |
| R2 Marg. | 0.820 |  |  |

**Supplementary Table 33:** **PDGFRβ^+^ labeling intensity in PDGFRβ^Cre^ and KLF4^Flox^ mice.** Posterior summary with parameter estimates (Est.) and 95% credible intervals (2.5% - 97.5%) for the multilevel model *Intensity ~ Area x Genotype + (1 | MouseID), family = student*.

| **Parameters** | **Est.** | **2.5 %** | **97.5 %** |
| --- | --- | --- | --- |
| b_Intercept | 0.2 | 0.09 | 0.4 |
| b_Area | 1.00 | 0.98 | 1.01 |
| b_GenotypeCre | -0.61 | -0.91 | -0.32 |
| b_GenotypeFlox | -0.20 | -0.54 | 0.11 |
| b_Area_GenotypeCre | -0.34 | -0.37 | -0.31 |
| b_Area_GenotypeFlox | -0.14 | -0.16 | -0.12 |
| sd_MouseID_Intercept | 0.19 | 0.10 | 0.36 |
| sigma | 0.12 | 0.11 | 0.13 |
| Num.Obs. | 1460 |  |  |
| R2 | 0.97 |  |  |
| R2 Marg. | 0.952 |  |  |
| ICC | 0.2 |  |  |

**Supplementary Table 34:** **Collagen-IV (ColIV) labeling intensity in PDGFRβ^KLF4-KO^ mice.** Posterior summary with parameter estimates (Est.) and 95% credible intervals (2.5% - 97.5%) for the model *Intensity ~ Genotype, family = student*.

| **Parameters** | **Est.** | **2.5 %** | **97.5 %** |
| --- | --- | --- | --- |
| b_Intercept | 283 | 193 | 374 |
| b_GenotypeKO | 9.9 | -111 | 136 |
| sigma | 120 | 79 | 176 |
| Num.Obs. | 18 |  |  |
| R2 | 0.025 |  |  |

**Supplementary Table 35: CD31 area in PDGFRβ^KLF4-KO^ mice.** Posterior summary with parameter estimates (Est.) and 95% credible intervals (2.5% - 97.5%) for the model *Area ~ Genotype, sigma ~ Genotype, family = student*.

| **Parameters** | **Est.** | **2.5 %** | **97.5 %** |
| --- | --- | --- | --- |
| b_Intercept | 3677 | 3399 | 3954 |
| b_sigma_Intercept | 5.8 | 5.2 | 6.5 |
| b_GenotypeKO | 272 | -472 | 999 |
| b_sigma_GenotypeKO | 1.05 | 0.23 | 1.86 |
| Num.Obs. | 18 |  |  |
| R2 | 0.044 |  |  |

**Supplementary Table 36:** **ColIV area in PDGFRβ^KLF4-KO^ mice.** Posterior summary with parameter estimates (Est.) and 95% credible intervals (2.5% - 97.5%) for the model *Area ~ Genotype, family = student*.

| **Parameters** | **Est.** | **2.5 %** | **97.5 %** |
| --- | --- | --- | --- |
| b_Intercept | 2843 | 2010 | 3688 |
| b_GenotypeKO | 699 | -466 | 1825 |
| sigma | 1155 | 788 | 1687 |
| Num.Obs. | 18 |  |  |
| R2 | 0.093 |  |  |

**Supplementary Table 37: ColIV area in PDGFRβ^KLF4-KO^ mice using Picrosirius staining.** Posterior summary with parameter estimates (Est.) and 95% credible intervals (2.5% - 97.5%) for the model *Area ~ Genotype, family = student*.

| **Parameters** | **Est.** | **2.5 %** | **97.5 %** |
| --- | --- | --- | --- |
| b_Intercept | 3757 | 2168 | 5352 |
| b_RegionCtx | 670 | 24 | 1904 |
| b_GenotypeKO | 442 | 12 | 1409 |
| b_RegionCtx_GenotypeKO | 511 | 14 | 1573 |
| sigma | 3974 | 3062 | 5150 |
| Num.Obs. | 36 |  |  |
| R2 | 0.024 |  |  |
